# Integrating multiple precision livestock technologies to advance rangeland grazing management

**DOI:** 10.3389/fvets.2025.1625448

**Published:** 2025-08-22

**Authors:** Lillian J. McFadden, Hector M. Menendez, Krista Ann Ehlert, Jameson R. Brennan, Ira L. Parsons, Ken Olson

**Affiliations:** ^1^Department of Animal Science, West River Research and Extension Center, South Dakota State University, Rapid City, SD, United States; ^2^Department of Natural Resource Management, West River Research and Extension Center, South Dakota State University, Rapid City, SD, United States

**Keywords:** precision livestock technology, data integration, nutrition models, open source code, rangelands, dry matter intake

## Abstract

Dry matter intake (DMI) of grazing animals varies depending on environmental factors and the physiological stage of production. The amount of CH_4_ eructated (a greenhouse gas, GHG) by ruminants is correlated with DMI and is affected by feedstuff type, being generally greater for forage diets compared to concentrates. Currently, there are limited data on the relationship between DMI and GHG in extensive rangeland systems, as it is challenging to obtain. Leveraging precision livestock technologies (PLT), data science, and mathematical nutrition models to predict DMI from enteric emission measurements of grazing cattle is likely a viable method, given the increase in available PLT for extensive systems. Therefore, our objectives were to: (1) measure CH_4_, CO_2_, and O_2_ emissions, DMI, and the weight of dry beef cows; (2) create a data pipeline to integrate three PLT data streams in Program R; and (3) use these data to develop a mathematical model capable of predicting grazing DMI. The predictive equation was developed using data from two feeding trials conducted using technology to measure enteric emissions, daily DMI, and front-end body weights. This study was conducted in western South Dakota with non-lactating Angus beef cows (*n* = 7) that received either moderate (15% crude protein, CP) or low (6% CP) quality grass hay using a 14-day adaptation period followed by a 14-day data collection period. Average CH_4_ (g/day), CO_2_ (g/day), and O_2_ (g/day) were 209 ± 60, 6,738 ± 1,662, and 5,122 ± 1,412 for the moderate group and 271 ± 65, 8,060 ± 1,246, and 5,774 ± 748 for the low-quality treatments, respectively. Initial models using emissions, O_2_ consumption, and body weight were not adequate for predicting individual DMI, with R^2^ values ranging from 0.01 to 0.28. Using smoothed herd-level data, the CH_4_ model produced the best results for predicting DMI (R^2^ = 0.77). This study presents a novel methodological approach to leverage data from multiple PLTs simultaneously, with the potential to advance DMI estimates for grazing cattle in rangelands.

## 1 Introduction

Dry matter intake (DMI; g/d) is a crucial measurement for various purposes, including determining nutrient requirements, evaluating feed efficiency, and calculating stocking rates in rangelands. Dry matter intake differs in beef cattle depending on the environment and the physiological stage of production (1). In rangeland grazing systems, a variety of beef cattle classes graze, which makes management difficult because strategies may differ by animal class (e.g., heifers and cows) as well as physiological status (e.g., growing, lactating). Balancing these factors with seasonal changes in crude protein (CP) and precipitation is important in determining forage intake, which ultimately impacts stocking rates. It is important to evaluate feed efficiency for producers to extend pasture availability, reduce supplement costs, and guide genetic selection within herds (2). Within grazing systems, the harvest efficiency metric is used to quantify the forage that is ingested by the grazing animal from the total forage biomass available in a pasture area and is reported as a percentage or decimal fraction (3). Smart et al. (4) employed three different intake equations for harvest efficiency and observed significant differences in harvest efficiency across stocking rates (*P* = 0.0001). With a goal of 25% harvest efficiency, the moderately stocked pastures were closest to this target, while heavily stocked pastures were 13%−16% higher, and lightly stocked pastures were 6%−10% lower (4). Although harvest efficiency provides a general view of the grazing performance of a herd as a whole, it makes it difficult to arrive at an individual DMI. Several methods have been developed to measure the DMI of grazing animals in pasture directly; however, these methods are complicated, time-consuming, and laborious, including direct observation, feed depletion, hand-plucking, total fecal collection, and internal/external markers (5, 6).

### 1.1 Dry matter intake and methane production

Research on beef cattle has shown that the consumption of non-cell wall components contributes to lower CH_4_ production compared to forages that have more cell wall components (7). This is because they are separated into soluble sugars, which create more CH_4_ than the starchy materials. Furthermore, differences in CH_4_ production are caused by additional fiber substrates available for microbial fermentation (i.e., low-quality forage), which results in methanogenesis. Most forage feedstuffs (e.g., hay or pasture forage) contain more fiber because of the structural components of cellulose, hemicellulose, and lignin. Thus, as fiber content increases in the animal diet, enteric CH_4_ generally increases compared to diets with larger proportions of less fibrous feedstuffs such as concentrates [e.g., ground corn (*Zea mays*)]. Methane has also been shown to be positively correlated with body weight [BW; *P* < 0.001; (8)]. This is because smaller animals ingest less feed; therefore, they proportionally emit less CH_4_ (9).

Dry matter intake was reported to be the most critical factor to predict CH_4_ production in a study conducted using an intercontinental database to create a prediction model for enteric CH_4_ production of beef cattle using data from Europe, North America, Brazil, Australia, and South Korea, especially with the groups of all data combined, high-forage diets, and lower-forage diets (10). As DMI increases, there is more material in the digesta that needs to be broken down for absorption, degradation, or passage, resulting in an increased microbial degradation time. Rumen microbes produce various proportions of volatile fatty acids (propionate, butyrate, and acetate) relative to fiber or concentrate levels—acetate production results in larger amounts of methanogenesis compared to butyrate and propionate. As cattle consume more forage compared to concentrates, the increased mean retention time (due to higher fiber being less digestible) allows for greater production of acetate and, thus, increased methanogenesis. This well-established relationship has enabled the exploration of methane production based on the DMI and nutrient composition of the feedstuffs. Many modeling studies have used regression, empirical, or mechanistic approaches based on this relationship (11–14). Consequently, the increased ability to collect enteric emission data from grazing cattle provides an opportunity to explore the correlation with DMI using mathematical models. Although field-based studies have laid the groundwork for estimating DMI, they are more tedious and time-consuming than models.

Nutrition models help researchers reduce costs and time when predicting DMI (15, 16). For example, empirical equations, such as using neutral detergent fiber (NDF) to predict DMI, are tools that may give DMI modeling reliability under similar environmental conditions (17). As the quality and quantity of animal data continue to improve, the reliability of DMI models is likely to increase. Animal data may include individual real-time measurements, such as weighing, feed intake, enteric emission monitoring, temperature, body condition, respiration, tracking of movement activity, and behavior. A major proponent of enhanced data collection for grazing livestock is the growing application of precision livestock technology (PLT) (19, 20), which includes the ability to collect CH_4_ data from grazing livestock to enhance DMI models.

### 1.2 The role of precision livestock technologies

Within extensive rangeland systems utilizing PLT, we can fill the gaps in research that have been infeasible in the past through the collection of high-resolution data that was previously unattainable (19). One crucial technology affecting rangeland research capabilities is the GreenFeed^TM^ pasture system (C-Lock Inc., Rapid City, SD) because it can be deployed on extensive rangelands (21). The GreenFeed^TM^ pasture system is a portable machine that can measure enteric gas flux, specifically CH_4_, CO_2_, and O_2_ consumption. When compared with the SF_6_ and chamber methods for enteric emission measurements, the GreenFeed^TM^ system has been shown to reliably and accurately measure CH_4_, CO_2_, and O_2_ from beef and dairy cattle in a pasture setting (21–24). Therefore, it may be possible to use PLT measurement tools, such as the GreenFeed^TM^, to estimate DMI in real time because CH_4_ and DMI are highly correlated, and the GreenFeed^TM^ provides a less labor-intensive (compared to SF_6_ on-animal devices), reliable, and long-term method to measure enteric emissions of grazing cattle, which was not possible until recently (25). However, leveraging PLT data in a transparent and repeatable manner to create such models is difficult and requires robust data integration pipelines and documentation. The objectives of this study were to: (1) measure CH_4_, CO_2_, O_2_, emissions, DMI, and BW of dry beef cows; (2) create a fully documented data pipeline to integrate three unique PLT data streams in Program R seamlessly; and (3) use these data to develop a mathematical model capable of predicting grazing DMI.

## 2 Methods

### 2.1 Institutional animal care and use approval

The SDSU Institutional Animal Care and Use Committee (IACUC) approved all procedures involving animals (approval number #A3958-01).

### 2.2 Study area

This study was conducted at the SDSU Cottonwood Field Station (CFS), located in western South Dakota (43.9604, −101.8579). The CFS is situated in a mixed grass prairie ecosystem and is composed primarily of native C3 mid-grasses, including green needlegrass (*Nassella viridula* [Trin.]), needle-and-thread (*Hesperostipa comata* [Trin. & Rupr.]), and western wheatgrass (*Pascopyrum smithii* [Rydb.]), intermixed with native C4 short grasses (blue gramma [*Bouteloua gracilis* Willd. Ex Kunth] and buffalograss [*Bouteloua dactyloides* (Nutt.)]). Recent introductions of non-native grasses, including kentucky bluegrass (*Poa pratensis* [Boivin & Love]) and japanese brome (*Bromus japonicus* [Thunb.]), are also prevalent at the site. The soil in the study area is predominantly Kyle clay and Pierre clay (51). The topography was gently sloping, with rolling hills and relatively flat-topped ridges, ranging from a peak elevation of 784 m to 710 m. The long-term (1991–2020) average annual precipitation at CFS is 452 mm (26).

### 2.3 Experimental treatments

**Figure 1:**
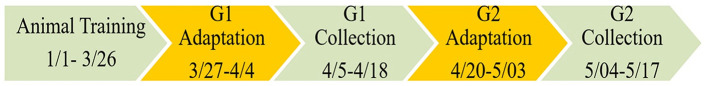
Diagram of animal training, adaptation, and collection phase dates for moderate (G1) and low (G2) diet treatments conducted in the winter of 2022 (January to May).

Dry beef Angus cows (*n* = 7, mean BW = 622 ± 11.79) were kept in a dry lot. Non-lactating/non-pregnant cows with constant nutritional demands were used to reduce the variation in energy and protein requirements for this experiment. The cows were fed two different forage-based diets *ad libitum* to mimic a rangeland setting. The two feed treatments were grass hay 1 (G1, whole-stem mixed grass hay with alfalfa) and grass hay 2 (G2, chopped mixed grass hay), which represented moderate and low forage nutrient compositions, respectively. The hay was acquired during a drought year; thus, the source of specific hay species or composition was limited. Each treatment consisted of a 2-week adaptation phase and a 2-week collection phase (Figure 1).

Daily samples were collected for each feeding period, dried, and weighed to determine dry matter percentage (*n* = 22). At the end of every 2 weeks, the forage was mixed into composite samples (*n* = 4), which were sent to Servitech Labs (Hastings, NE, USA) for testing in triplicate (*n* = 12). Forage nutrient analysis of each grass hay in each phase was conducted to determine the dry matter (DM%), crude protein (CP%), and acid detergent fiber (ADF%) content. These measurements were used to estimate the total digestible nutrients (TDN%). During the collection periods for G1 and G2, we collected individual enteric emissions (g/hd/d), individual daily DMI (kg/hd/d), and individual daily cow weights (kg/hd/d).

### 2.4 Precision technologies

**Figure 2:**
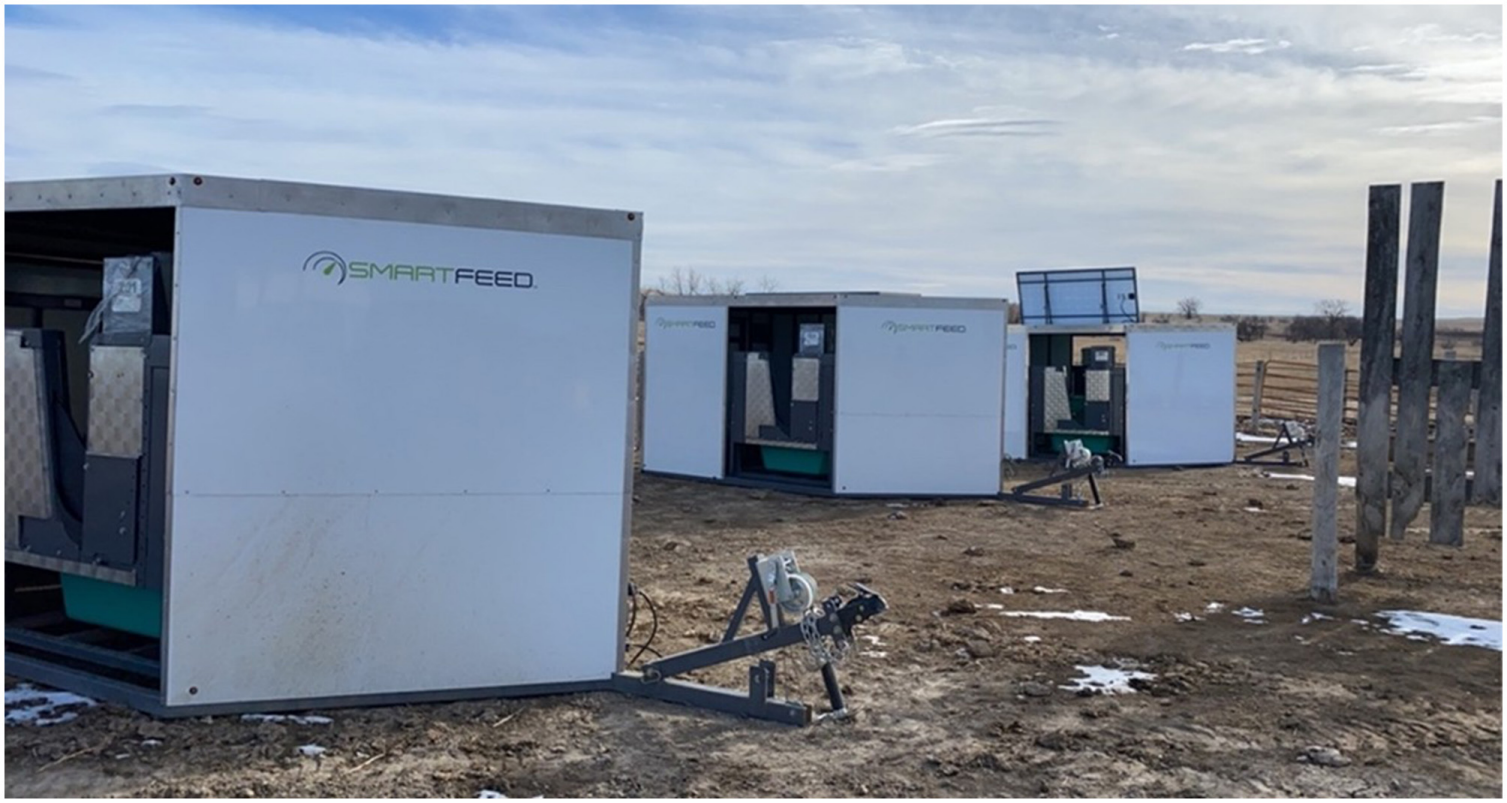
Set up of three mobile SmartFeeder^TM^ units at the South Dakota State University Cottonwood Field Station dry lot (Cottonwood, SD). Each feeder contains two precision feeding bunks.

To collect these measurements, we used three precision measurement technologies: the SmartFeeder^TM^, GreenFeed^TM^, and SmartScale^TM^ (C-Lock Inc., Rapid City, SD, USA). All devices followed suggested experimental protocols (i.e., weight and gas calibrations) to ensure data quality throughout the experiment (27). The SmartFeeder^TM^ was used to collect daily individual intake by measuring disappearance (Figure 2). To achieve this, the device calculated the total feed in the bin (kg) minus the disappearance (kg) for each feeding event (28), resulting in intake values on an as-fed basis per cow (kg/h/d). Later intake was converted to DMI using the percent dry matter values from the processed forage.

**Figure 3:**
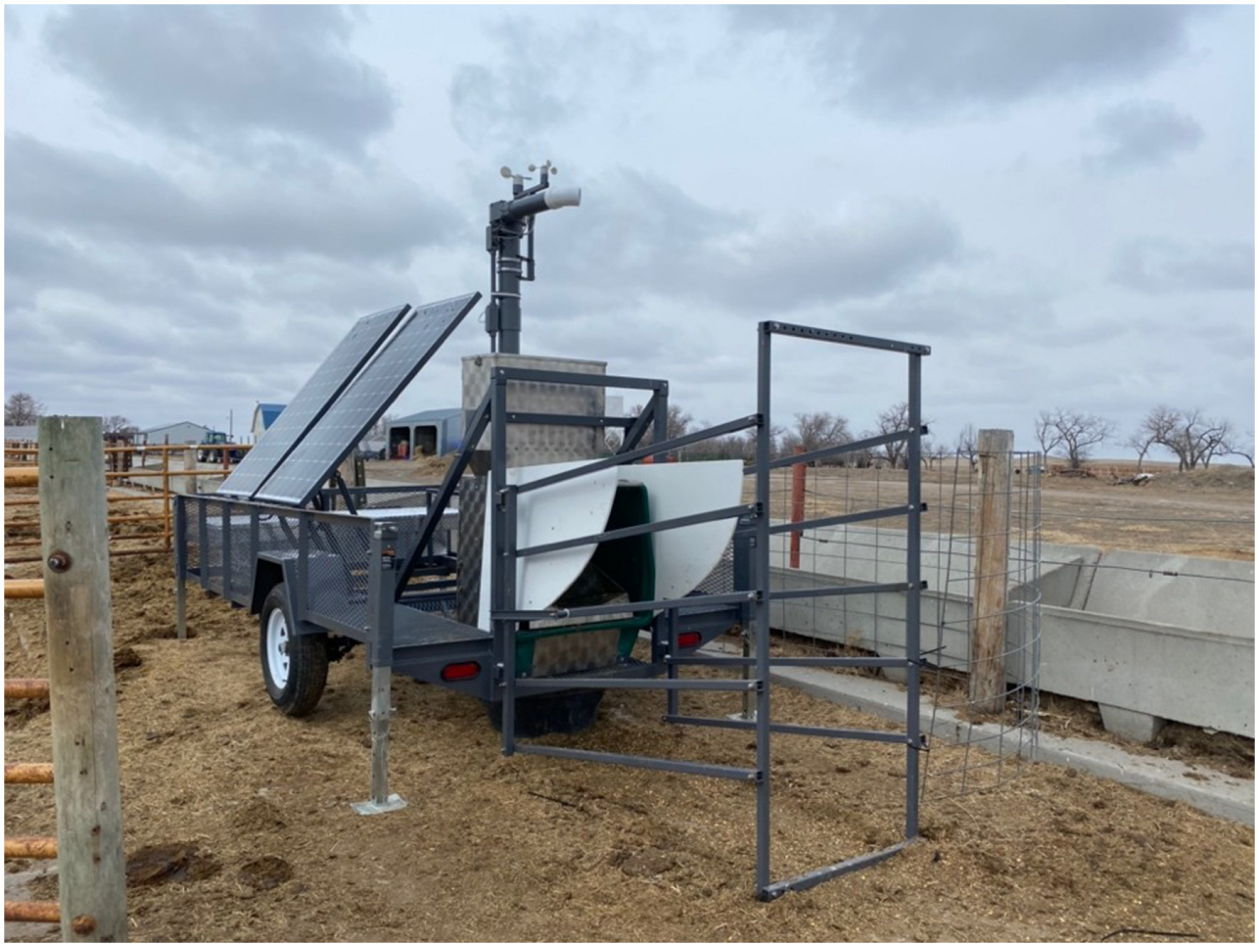
GreenFeed^TM^ Unit 297 deployed at the South Dakota State University Cottonwood Field Station drylot (Cottonwood, SD).

The GreenFeed^TM^ (Figure 3) was used to measure CH_4_ and CO_2_ emissions and O_2_ consumption (g/hd/d) from the cows on an individual basis in real time. The GreenFeed^TM^ uses radio frequency identification (RFID) tags that are unique to each animal. The cattle were baited into the headbox of the GreenFeed^TM^ using an alfalfa (*Medicago sativa*) pellet (CP = 15%, ADF = 38%, and NDF = 48%). Alfalfa pellets were selected for alignment with the forage-based treatments. The GreenFeed^TM^ fed cattle at a rate of ~35 g every 30 s with eight drops for each feeding period (Equations 1–6). Each cow could receive a maximum of five feeding periods per day, which, if consumed, would result in a 29.5% maximum potential contribution of CP to the basal diet in the current study (i.e., 0.81 kg instead of only 0.627 kg).


(1) $$\begin{array}{l} Maximum\;Pellets\;Fed\;=Feeding\;Periods\;\times Drops\;Per\;Period\\ \;\times Drop\;Mass \end{array}$$

(2) $$\begin{array}{l} Maximum\;Pellets\;Fed\;DM\;Basis=Maximum\;Pellets\;Fed\\ \;\times \left(1-\;Pellet\;Moisture\;\%/100\right) \end{array}$$

(3) $$\begin{array}{l} Pellet\;CP=\;Maximum\;Pellets\;Fed\;DM\;Basis\\ \;\times \;\left(Pellet\;\%\;CP/100\right) \end{array}$$

(4) $$\begin{array}{l} Basal\;Diet=BW\;\times \left(1.8\%/100\right) \end{array}$$

(5) $$\begin{array}{l} Basal\;Diet\;CP=Basal\;Diet\;\times \left(Basal\;Diet\;CP\;\%/100\right) \end{array}$$

(6) $$\begin{array}{l} Pellet\;CP\;Percent\;of\;Basal\;Diet=Pellet\;CP\;÷Basal\;Diet\;CP \end{array}$$

Where Maximum Pellet Fed is 1.4 kg/d, Feeding Periods = 5/d, Drops Per Period = 8, Drop Mass = 35 g, Maximum Pellet Fed on DM basis is 1.232 kg/d, and pellet moisture is 12% [crude protein (CP)]. Pellet CP = 0.1848 kg; Pellet % CP = 15. Basal Diet = 11.196 kg, BW = 622 kg, 1.8 is the percent DMI per BW factor, Basal Diet CP = 0.627 kg/d; Basal Diet CP% = 5.6, Pellet CP Percent of Basal Diet is 29.47% (see Supplementary Section 19 for complete details).

When an animal is consuming pellets distributed by the GreenFeed^TM^ system (≥2 min required), the system measures the airflow rate, background CH_4_, and CO_2_ concentration. Thus, it can measure gas (CH_4_ and CO_2_) fluxes from the animal. The non-dispersive infrared analyzer and head proximity sensor then filter out samples where the head is not in an optimal position to provide satisfactory measurements (21). The GF unit used in the current study also measured the O_2_ consumption.

**Figure 4:**
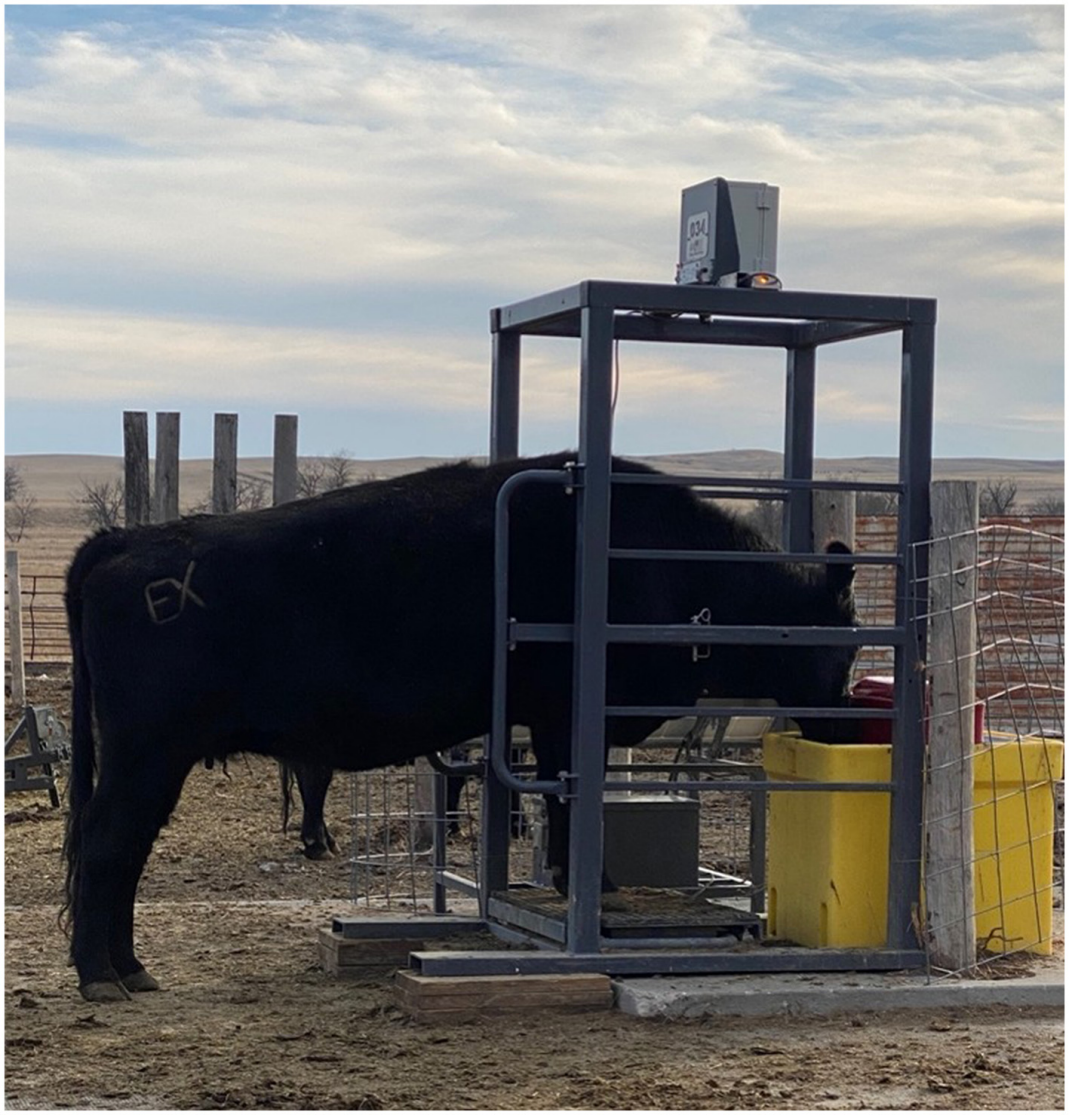
Example of a cow using the SmartScale^TM^ attached to the Ritchie Livestock Waterer in the drylot at the South Dakota State University Cottonwood Field Station (Cottonwood, SD).

The Smartscale^TM^ (Figure 4) was used to measure individual front-end weights that were then converted to full BW (29), using independent full BW that were taken using a conventional scale collected at the beginning and end of each phase (*n* = 35). The chute weights were collected using a hydraulic squeeze chute (Silencer™, Stapleton, NE) on load cells and bars (Tru-Test, Mineral Wells, TX, USA).

### 2.5 Data pipeline

For all three PLTs, RFID tags were used so that the collected data could be paired with individual animals. We later combined all three datasets into a single data pipeline (see Supplementary code and data). All data and results presented in this study are reproducible using the Supplementary materials and the corresponding sections referenced (1–19 in the R-Markdown section). Data were sent to the cloud remotely and downloaded either through a direct download or application programming interface [API; (30)] from the C-Lock, Inc. web interface and entered into Program R. Data collection from multiple PLTs resulted in a large amount of data that needed to be cleaned and processed into a usable format, and an interquartile range was used to remove DMI and BW outliers (Supplementary Sections 1–13).

To process the data and conduct a statistical analysis, we integrated these three precision data streams into the R programming language for Statistical Computing [RStudio version 4.3.1; (31)]. Using R Studio, we ran descriptive statistics, removed outliers, and checked for normality, homoscedasticity, and independence (Supplementary Section 15). A mixed-model analysis of variance (ANOVA) (lme4 package, *P* < 0.05) (32) was conducted for each gas (CH_4_, CO_2_, and O_2_) and DMI to determine the differences between the two treatments (G1 and G2; *P* < 0.05). We used a mixed model ANOVA instead of a one-way ANOVA because of the lack of independence between variables; treatment was the main effect, and animal was the random effect (Supplementary Sections 14, 15).

### 2.6 Comparing contemporary intake models

We compared the observed DMI dataset with two DMI equations for beef cattle requirements, the first being National Academies of Sciences Medicine et al. (15), which uses metabolic BW and net energy for maintenance, as shown in Equations 7, 8 (Supplementary Section 16):


(7) $$\begin{array}{l} \text{NEm Intake}=\text{B}{\text{W}}^{0.75}\times \left(0.04997\times {\text{NEm}}^{2}+\;0.04631\right) \end{array}$$

where NE_m_ Intake is the net energy for maintenance intake (Mcal/d), BW^0.75^ is the metabolic BW (kg), and NE_m_ is the net energy for maintenance (Mcal) required by the animal.


(8) $$\text{DMI = }\frac{\text{Total }{\text{NE}}_{\text{m}}\text{ Intake }}{\text{Dietary }{\text{NE}}_{\text{m}}\text{ Concentration }}$$

Where DMI is the dry matter intake (kg), Total NE_m_ intake is the amount of energy consumed by the animal, and the Dietary NE_m_ Concentration is the concentration of energy in the feed/forage consumed (Mcal/day) and was calculated using the total digestible nutrients of each forage type [G1 and G2; Menendez and Tedeschi (18)].

The second equation is used primarily for calculating rangeland stocking rates (hd/ha/time) and is based on the DMI as a percentage of BW (Equation 9):


(9) $$\begin{array}{l} \text{DMI }=\text{ BW X }\left(1.8\text{\%}/100\right) \end{array}$$

Where DMI is dry matter intake (kg/d) and BW is the cow's body weight (kg) multiplied by 1.8%. This percentage was used because a non-lactating cow is generally assumed to consume 1.8% of their BW (33).

We then evaluated the predictions of the NASEM and percent BW DMI equations against the observed DMI using a Model Evaluation System [MES; (34)] to assess precision (R^2^) and accuracy (mean bias; MB). The coefficient of determination (R^2^) measures the proportion of variance that connects the observed and predicted values, with values closer to 1 indicating greater precision (35, 36). The MB specifies the differentiation of means between the observed and predicted values (values closer to 0 being better) (37).

### 2.7 Predictive DMI model development

After processing the data, we utilized a linear regression and multiple linear regression approach to predict the DMI from enteric emissions and BW in Program R (Supplementary Section 17). First, we regressed the DMI using CH_4_, CO_2_, O_2_, and BW for each treatment (G1, G2). We then regressed the same individual covariates against DMI using all data (i.e., a combination of treatments G1 and G2). For multiple linear regression, DMI was regressed against a combination of all covariates, both by treatment and the combination of treatment data. The Corrected Akaike Information Criterion (AICc) was used to select the best models, in addition to filtering using the variance inflation factor (VIF) to remove variables that caused multicollinearity (Supplementary Section 17).

Due to the small sample size and gaps in the data, all individual data points (the entire study) were combined daily into a herd average. This increased the available data; however, the data were still limited and inconsistent across time, so a smoothing function was used on the observed dry matter and gases to allow previous observations to impact future observations. We used exponential smoothing in Program R (Supplementary Section 19). The data were smoothed based on the last seven days of the data. This reduced the amount of noise around the data (extreme highs and lows). Using the entire dataset allows for better smoothing over time (because more observations can be used to smooth the data). The smoothed data was then subset back into the G1 and G2 data sets. These herd-level smoothed data were then used to reanalyze the same DMI models mentioned previously (Supplementary Section 17) for the G1 and G2 datasets at the herd level.

## 3 Results

**Figure 5:**
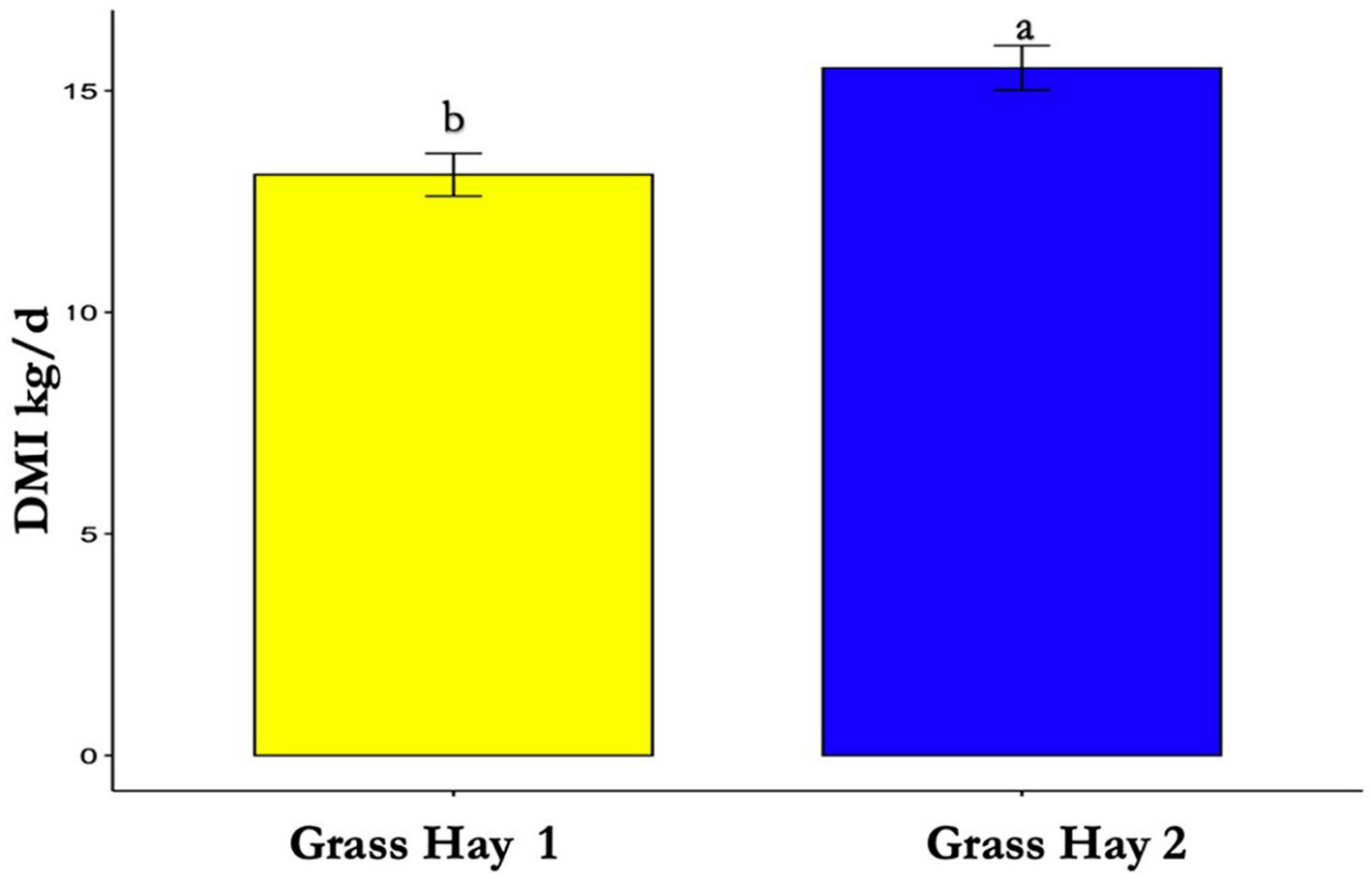
Differences in average dry matter intake (DMI) between treatments (*P* < 0.05). Where differences in letters, “a” and “b”, designate a statistical difference in means.

**Figure 6:**
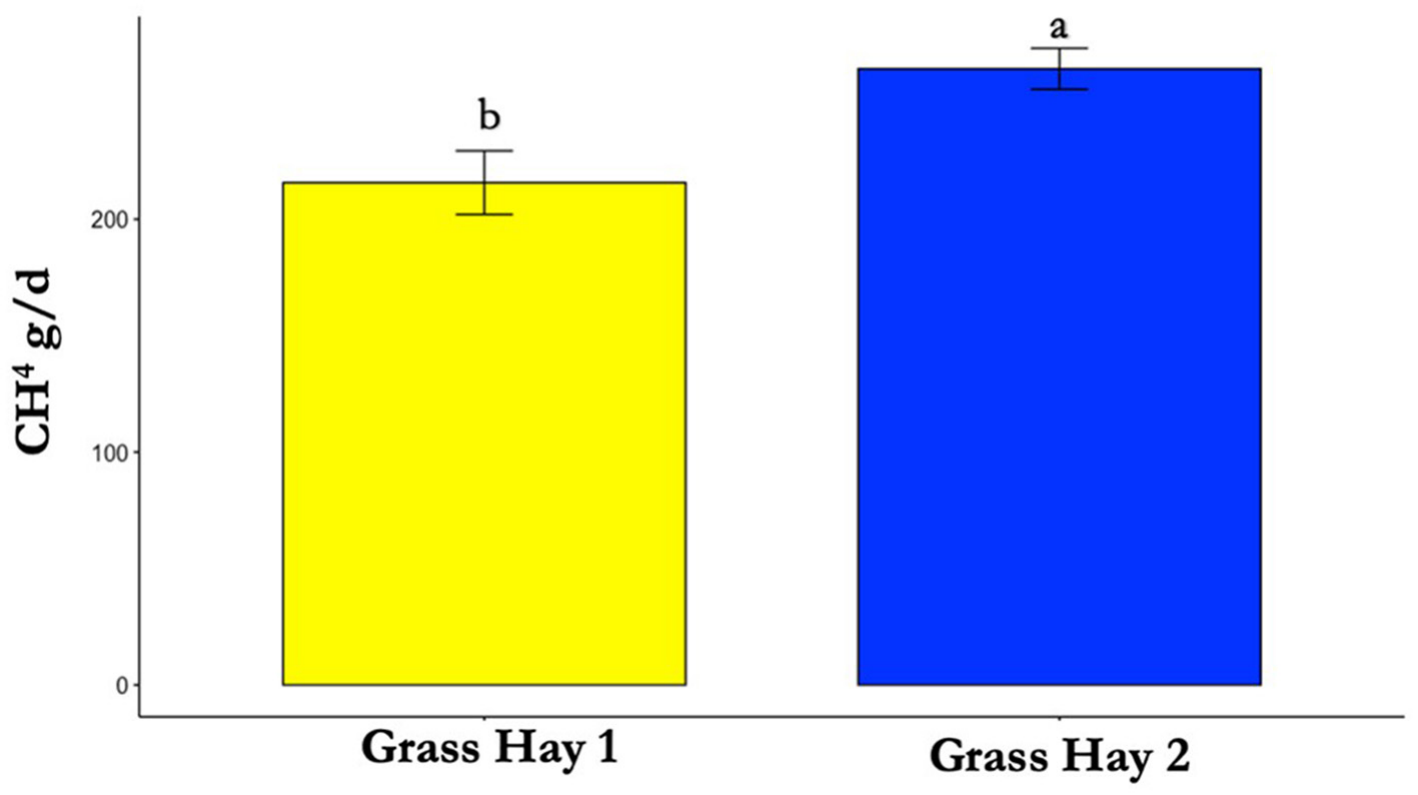
Differences in average methane (CH_4_) production between treatments (*P* < 0.05). Where differences in letters, “a” and “b”, designate a statistical difference in means.

**Figure 7:**
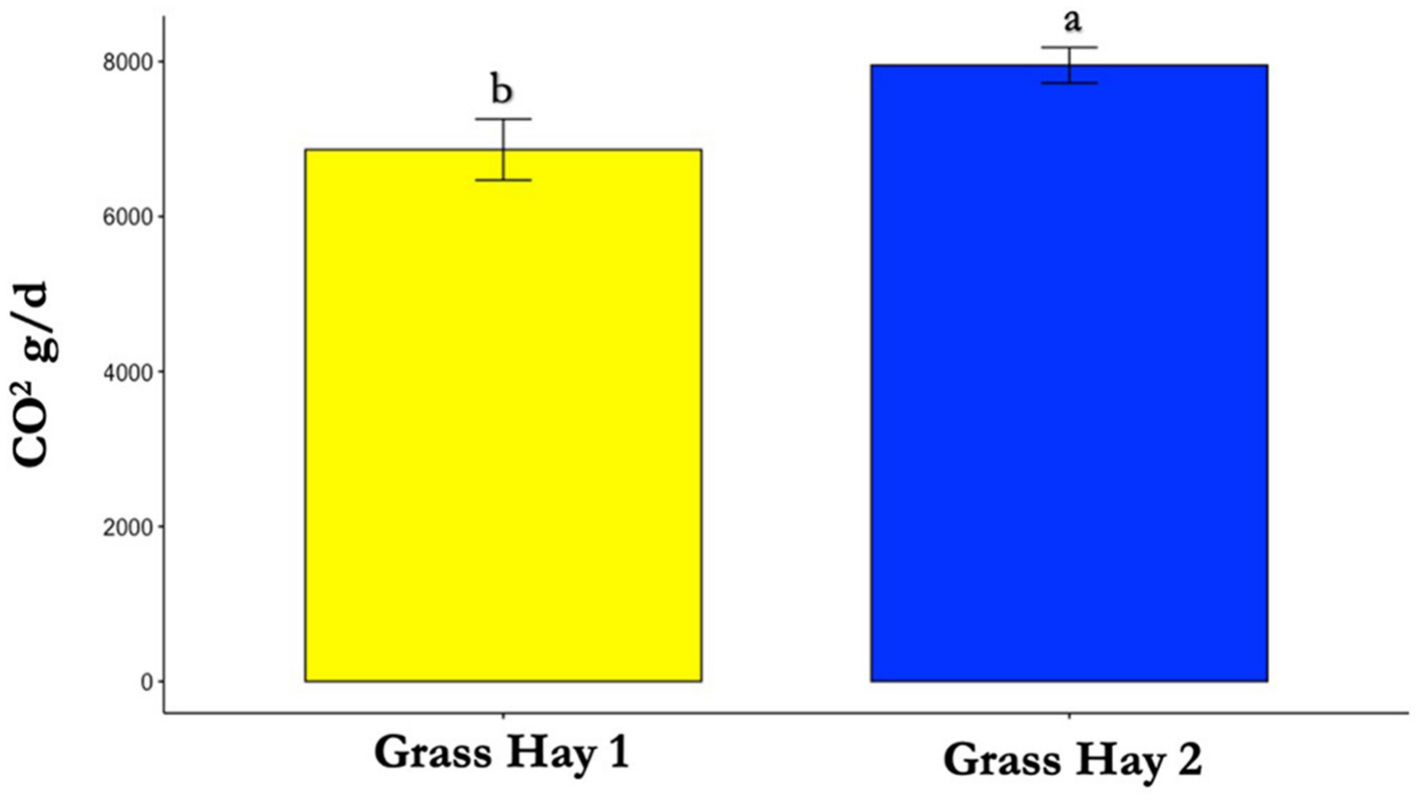
Differences in average carbon dioxide (CO_2_) production between treatments (*P* < 0.05). Where differences in letters, “a” and “b”, designate a statistical difference in means.

**Figure 8:**
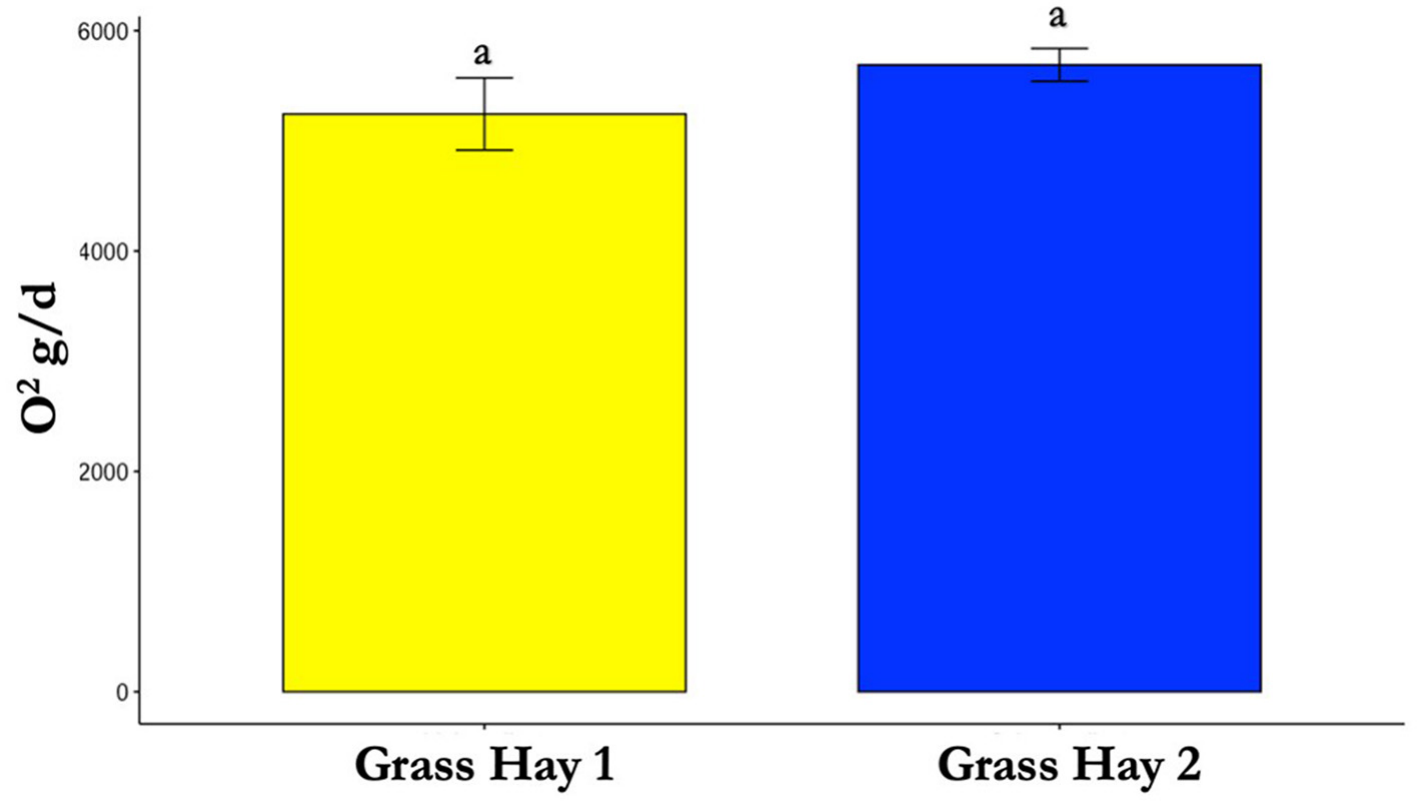
Differences in average oxygen (O_2_) consumption between treatments (P > 0.05). Where differences in letters, “a” and “b”, designate a statistical difference in means.

The results of the nutrient analysis indicated that the nutrient composition of G1 forage had higher CP and TDN and lower ADF compared to the G2 diet. The CP and TDN for G1 were higher by 59.71% and 14.08%, respectively. However, the ADF of G2 was 12.64% higher (Table 1). Dry matter intake and emissions data showed that G2 had a higher average DMI, CH_4_, CO_2_, and O_2_ by 15%, 23%, 16%, and 11%, respectively (*P* < 0.05; Table 2; Figures 5–8; Supplementary Section 13). The ranges for each gas per treatment showed that G2 had a greater range for CH_4_, whereas G1 had a greater range for CO_2_ and O_2_ (Table 2). Methane and CO_2_ levels were significantly different (*P* < 0.05) between treatments, but O_2_ consumption was not significantly different (*P* > 0.05; Supplementary Section 14).

**Table 1:** Nutrient analysis results from the moderate (G1) and low (G2) forage treatments.

**Feed treatment**	**%DM**	**%CP**	**%ADF**	**%TDN**
G1	93.3	13.9	38.7	55.4
G2	93.4	5.6	44.3	47.6

The feed was tested for dry matter (%DM), crude protein (%CP), and acid detergent fiber (%ADF), which were then used to calculate total digestible nutrients (%TDN).

**Table 2:** Dry matter intake (DMI), methane (CH_4_), carbon dioxide (CO_2_), and oxygen (O_2_) enteric emissions (g) averages (AVG and standard deviation) and ranges (RG) for each treatment (moderate-quality grass hay 1 = G1 and low-quality grass hay 2 = G2) and the combined treatment data.

**DMI (kg) and gases (g)**	***n***	**G1**	***n***	**G2**	**Combined**
AVG DMI	18	12 ± 1.8	26	14 ± 2.2	13 ± 1.9
AVG CH_4_	18	209 ± 60	26	271 ± 65	240 ± 63
RG of CH_4_	-	105–322	-	126–443	105–443
AVG CO_2_	18	6,738 ± 1,662	26	8,060 ± 1,246	7,399 ± 1,453
RG of CO_2_	-	3,952–9,822	-	5,923–10,227	3,952–10,227
AVG O_2_	18	5,122 ± 1,412	26	5,774 ± 748	5,448 ± 1,080
RG of O_2_	-	2,149–7,532	-	4,348–7,046	2,149–7,046

When we evaluated observed DMI data compared to estimated DMI using the NE_m_ Intake and %BW equations, we found that both NASEM and %BW had similar levels of precision until data were combined across treatments, which resulted in the %BW estimate being more precise. In general, all DMI equations underpredicted the DMI, except for NE_m_ Intake in G1 (Table 3; Supplementary Section 16).

**Table 3:** Predicted dry matter intake (DMI) using NASEM (15) and 1.8% body weight (BW) equations was regressed against the observed DMI for moderate-quality grass hay (G1), low-quality grass hay (G2), and combined treatments.

**Model to predict DMI**	**R^2^**	**MB %**
G1: NASEM	0.44	0.93
G1: BW	0.44	−1.82
G2: NASEM	0.75	−1.72
G2: BW	0.74	−2.78
Combined: NASEM	0.33	−0.64
Combined: BW	0.72	−2.38

Using the levels of precision (R^2^) and accuracy [mean bias (MB%)], see Supplementary Section 18.

Variance inflation factor (VIF) filtering indicated that multicollinearity existed when attempting multiple linear regression, and therefore, these models were not selected (VIF threshold ≤ 5). Using the AICc-selected linear regression models for each treatment and the combined results, the most precise model was found to be the DMI by CH_4_ for the G1 treatment, compared to all other covariates and treatment combinations (R^2^ = 0.28). For the G2 treatment, the highest R^2^ value was 0.014 for DMI by CO_2_, indicating no significant correlation (*P* > 0.05). Using the combined treatment data (G1 and G2), the highest R^2^ value was 0.19 for DMI CH_4_. The linear regression results were deemed unsatisfactory for predicting DMI (Supplementary Section 17). Re-evaluation of the ability of the best models to predict DMI (see above) using herd-level smoothed data resulted in an adjusted R^2^ of < one for G1 and G2 and a mean bias near zero. However, the combined G1 and G2 model (DMI~CH_4_) had an adjusted R^2^ of 0.77 and a mean bias near zero (Table 4; Supplementary Section 18C).

**Table 4:** Smoothed herd average models were deployed to predict dry matter intake (DMI) using moderate-quality grass hay (G1), low-quality grass hay (G2), and treatments combined (G1 and G2) compared to the observed smoothed herd-level DMI (Supplementary Section 18C).

**DMI models**	**Adjusted R^2^**	**Mean bias**
G1: CH_4_	0.070	0
G2: CO_2_	−0.002	0
Combined: CH_4_	0.770	0

### 3.1 Discussion

Conducting an experiment using PLTs requires the ability to clean and organize data into a consistent format, which is a significant barrier to the effective implementation of precision technology (19). This data barrier is substantially increased when multiple PLTs are utilized. In the current study, a data pipeline was successfully developed to integrate data from the SmartFeeder^TM^, GreenFeed^TM^, and SmartScale^TM^ technologies. One key challenge in the current study was identifying a unique attribute to organize the multiple datasets. We overcame this issue by creating a separate “ID” column to merge by date and RFID (“AnimalTag”). Once large amounts of data are collected and organized into a single data frame, there may be missing data from certain times/dates. Missing of data occurs when animals do not use the technology or when communication or hardware/software errors occur. Thus, when using PLTs, users should plan on having a larger sample size than required and understand the PLT's strengths and weaknesses to minimize missing data (38). Open-source data pipelines for specific single or multiple PLTs help expedite future researchers' ability to clean and combine data when using similar technologies (29). This is critical because programs such as Excel™ are insufficient to handle the volume of data generated by PLTs. Another major limitation of precision data is their utility in mathematical models, as models require consistent datasets. Unless automatic interpolation is incorporated, they cannot produce reliable results (38, 39). A critical next step for precision livestock research is to further enhance the pipeline developed in the current study by incorporating open-source code examples and tutorials, which will accelerate PLT research and broader applications as PLT technology use increases [see Supplementary code and data; (29)].

### 3.2 Technology challenges

Deploying precision technology provides a new means of experimentation, data collection, and model development. As expected, several challenges were encountered for each technology. The SmartFeeder^TM^ was not created to be used with chopped hay, requiring us to clean out the headgate to prevent it from getting jammed. Wind also played a factor because it can blow forage out of the tub; however, it is accounted for on the C-Lock web interface as being categorized as an “Unknown” disappearance. Finally, the correct tub height is a crucial factor. If the tubs are too tall, cows will not be able to utilize the full amount of feed provided and will have to be fed more frequently. If they are too low, then the cows can be more selective and pull out feed or push feed over the lip of the tub, potentially biasing the DMI data. Further, training cattle to use the GreenFeed^TM^ is difficult, and they need adequate time to adjust to this machinery. Baiting animals with a more palatable feed is a good way to get them close enough to interact with the GreenFeed^TM^. As previously mentioned, not all cows will use the GreenFeed^TM^, so having an initially large sample size is essential for achieving adequate samples after culling non-adopters, something not accounted for in a statistical power test. The SmartScale^TM^ had no significant limitations.

In the current study, the individual DMI data for G1 and G2 treatments were found to be reliable, ranging between 10.22 and 20.52 kg for cows weighing 509–783 kg. On average, cattle in our study consumed 2.3% BW, consistent with another study that reported similar DMI ranges (2.2%−2.9% BW) for dry beef Angus cows (535–564 kg BW) (40). As more individual cow DMI data become available, there may be the potential to assess DMI more closely in relation to weight, body condition score, production stage, genetics, and individual efficiency.

In terms of enteric emissions, we found comparable emission levels with those reported in other studies on beef cows. The average CH_4_ was 240 g/d and ranged from 105 to 443 g/d for G1 and G2 (Table 2). Whereas McGinn et al. (23) reported an average of 309 g/d, and Guyader et al. (41) reported a range of 143–372 g/d (23, 41). Our average was 7,399 g/d for CO_2_ emissions and ranged from 3,952 to 10,227 g/d (Table 2), which is comparable to the reported average CO_2_ of 8,223 g/d by McGinn et al. (23). Oxygen emissions from G1 and G2 in the current study averaged 5,448 g/d and ranged from 2,149 to 7,046 g/d (Table 2). Previous numbers have been reported, but these O_2_ averages were 7,922 (g/d), 32% higher than those we collected (42). Comparable enteric emission results are significant because they indicate the GreenFeed^TM^ measurements in the current study were appropriate for developing an enteric-based DMI model.

The current study was limited by a low and inconsistent GreenFeed^TM^ sample size, which likely reduced correlations between observed DMI and enteric emissions measurements. The GreenFeed^TM^ emission monitoring system was used to determine the repeatability of CH_4_ and CO_2_ emissions using 28 beef heifers in a dry lot pen for 59 days (21). Overall, they found over a 7- or 14-day sampling period that the GreenFeed^TM^ system-produced measurements with low variability in gas emissions and yield (gas/standardized DMI). Additionally, high repeatability and correlation with gases and feed intake were determined (CH_4_ = 0.50, CO_2_ = 0.62); however, 1- or 3-day samples resulted in larger variability in emissions and lower correlation with DMI (21). Thus, animals that do not visit the GreenFeed^TM^ can often produce large errors within the averages of samples. Manafiazar et al. (21) noted that there is potential for the GreenFeed^TM^ system to represent animal CH_4_ and CO_2_ emissions and yield with a 7 to 14-day sampling period and 20 or more samples per animal. Although this helps to determine general herd-level CH_4_ estimates, it fails to account for individual enteric emissions and diurnal variations in CH_4_ emissions.

For example, using 14-day derived values for beef cows will result in overestimation or underestimation when multiplied by the number of animals in the herd. This is because individual changes in enteric emission production rates (e.g., g CH_4_ per hour) change relative to rumen fill and nutrient composition of the digesta (16). Further, our study demonstrated the need to assess sub-daily and seasonal variation of forage quality as the quantity and quality of GreenFeed^TM^ data improves for individual animals in rangeland settings.

### 3.3 Evaluated differences in treatments

We identified significant differences in DMI, CH_4_, and CO_2_ between G1 and G2. However, we expected that cows in the G1 treatment would have consumed more than in the G2 treatment due to the higher CP and TDN levels. It is possible that the hay processing methods impacted daily consumption rates. For example, the moderate treatment (G1) was flaked hay pulled from large square bales, whereas G2 was chopped hay; cattle preferred the latter. Hay availability and processing were limited due to the persistent drought in 2021 before the study, hindering our ability to secure different hay qualities that were processed similarly. Thus, an opportunity to improve this study in the future is to have consistent hay sources and processing, as well as a broader range of nutrients. For example, securing hay from the same field at different dates would provide the desired treatments and reflect the CP variation of forages throughout the growing season, allowing the study to capture a potentially wider response in enteric emissions. However, the differences in enteric emissions we found were consistent with the known relationship between high fiber (G2) and increased emissions compared to lower fiber (G1) content (7). A critical next step is likely to account for the individual GreenFeed pellet CP contribution impacts to provide an adjustment factor, if needed (i.e., if there is an indication of differences in CH_4_ from pellet consumption rates). However, Raynor et al. (43) reported some effects of diet on CH_4_ production in intensive grazing systems between local and naïve steers.

### 3.4 Modeling

Many models have been developed for DMI for beef cattle (5, 6, 44). However, it is critical to keep the development of new models as simple as possible (16, 35, 45). Given the size of our dataset and the models' purpose, our use of regression modeling was the appropriate first step compared to more advanced artificial intelligence (AI) modeling approaches (38).

### 3.5 DMI regression models

Linear regression was not satisfactory for estimating the DMI using the available dataset. Although it was unsatisfactory for our study, previous research has shown a strong correlation between DMI and enteric emissions. Satisfactory levels have been reported as 0.63 R^2^ (46). Furthermore, another study reported that predictive performance can often be a neglected aspect when assuming that machine learning (ML) algorithms are the only or supreme modeling technique (47).

The most precise modeling results were achieved when we repeated our regressions using our original raw data on a herd average and applied 7-day smoothing instead of an individual DMI. Though manipulation of the data should be done with caution to avoid false levels of model confidence and adequacy of predictions. However, the smoothing function deployed in the current study may be a viable alternative when there are data gaps, allowing for the accurate interpolation of data across time (in the current study, all mean bias values were close to zero in the models). The accurate but not necessarily precise data can inform precision feeding models that require continuous data, rather than having a zero value (e.g., livestock require an allocation of feed regardless of the level of precision of data).

### 3.6 Incorporating body weight

Body weight can give producers a rough estimate of how much their cattle may be consuming on rangeland, but as our study showed, the %BW DMI equation is moderately precise in estimating the DMI. This lack of precision can be due to the exclusion of key factors, such as rumen fill, forage quality, or metabolic requirements, when BW alone is used (1, 48). Other studies have used the forage net energy equation, which incorporates individual shrunken BW and standing forage NE_m_ concentrations (49). In a study conducted by Undi et al. (49), standing forage was estimated using hand-plucked samples to mimic the forage that animals would consume. They also used the Minson equations, which employ BW and ADG of individual animals (49, 50). It was determined that the forage net energy equation had a DMI range of 0.6%−4.7% BW, and the Minson equation predicted an intake of 0.9%−2.2% BW with an average of 1.7% and 2.3% BW, respectively. Although the DMI values differ, the Minson equation, which uses BW, is the least variable when compared to other DMI predictions used in the study (49). Overall, BW equations are uncomplicated, but they do not consider the outstanding factors that may affect intake (15).

## 4 Conclusion

The current study results set a baseline for rangeland cattle enteric emissions and oxygen consumption and highlight the need for further research into other animal classes regarding enteric emissions and DMI. Additional data for the different phases are critical because the dry phase is relatively short (< 3 months) compared to the pregnant or lactating phases, which combined represent >15 months. Therefore, future studies may incorporate different animal classes that provide varying degrees of emissions and DMI. We were successful in developing a methodological approach (data pipeline) to more adequately address research that integrates multiple PLTs simultaneously and leverages data for mathematical nutrition modeling for ruminants on forage-based systems. In the future, the development of a data pipeline for integrating multiple PLTs is likely to advance investigations into DMI prediction and other studies of interest that can be conducted using a repeatable process. With an improved understanding of the impact of DMI on GHG emissions from beef cattle, we can facilitate further discussions on ways to mitigate GHG emissions in the cow-calf sector. For example, precision-derived DMI estimates using PLT have the potential to reduce overgrazing of rangeland by 458,812 ha in South Dakota alone (20). Thus, using PLT systems to improve DMI estimates and, consequently, stocking rates is an important tool for enhancing the efficiency, productivity, and competitiveness of the U.S. beef cow-calf production sector.

## Data Availability

The original contributions presented in the study are included in the article/Supplementary material, further inquiries can be directed to the corresponding author.
