# Supplementary material for: Integrating multiple precision livestock technologies to advance rangeland grazing management
Source: Front Vet Sci. 2025 Aug 22;12:1625448. doi: 10.3389/fvets.2025.1625448 (PMC12411203; doi:10.3389/fvets.2025.1625448)
Supplement: Supplementary file 2 [file Data_Sheet_2.PDF]

Analysis of DMI and Enteric Emissions

Lillian McFadden and Drs. Hector Menendez, Jameson Brennan, and Ira Parsons

2025-07-18

`{r} setup, include=FALSE} knitr::opts_chunk$set(echo = TRUE)`

```
##                _
## platform       x86_64-w64-mingw32
## arch           x86_64
## os             mingw32
## crt            ucrt
## system         x86_64, mingw32
## status
## major          4
## minor          3.1
## year           2023
## month          06
## day            16
## svn rev        84548
## language       R
## version.string R version 4.3.1 (2023-06-16 ucrt)
## nickname       Beagle Scouts
```

1. Load Required Packages

```
# Install required packages if not already installed
packages <- c("readxl", "ggplot2", "car", "ggplot2", "writexl", "lme4", "lmerTest",
             "emmeans","multcomp", "multcompView","tidyverse", "broom", "openxlsx", "zoo",
             "dplyr")
install_if_missing <- function(pkg) {
  if (!requireNamespace(pkg, quietly = TRUE)) install.packages(pkg)
}
lapply(packages, install_if_missing)

# Load packages
library(readxl)
library(ggplot2)
library(car)
library(writexl)
library(lme4)
library(lmerTest)
library(emmeans)
library(multcomp)
library(multcompView)
library(tidyverse)
library(broom)
library(openxlsx)
library(zoo)
library(dplyr)
```

2. Import and Inspect Data

Where Intake is the as fed individual intake data from the Smart Feeder, Drymatter is the percent dry matter measured in triplicate for each trial period, Weights is the Smart Scale weights of each individual animal, and Enteric is the gas emissions and oxygen consumption data from the GreenFeed for each individual animal.

```
# Look at sheet names in the Excel file
readxl::excel_sheets(path = "Data/intake.final.xlsx")
readxl::excel_sheets(path = "Data/Forage_Data.xlsx")
readxl::excel_sheets(path = "Data/ScaleWeights.xlsx")
readxl::excel_sheets(path = "Data/GreenFeed_Summarized_Data_298_2022_02_01_To_2022_05_17.xlsm")
# Load the intake data
Intake <- readxl::read_excel("Data/intake.final.xlsx", sheet = "Sheet1")
Drymatter <- readxl::read_excel("Data/Forage_Data.xlsx", sheet = "Sheet1")
Weights <- readxl::read_excel("Data/ScaleWeights.xlsx", sheet = "Sheet1")
Enteric <- readxl::read_excel("Data/GreenFeed_Summarized_Data_298_2022_02_01_To_2022_05_17.xls
m", sheet = "Visit_Data")
```

3. Review Column Names and Adjust

Here we fix column names so that data frames can be merged and for consistent plots.

```
head(Intake)
```

```
## # A tibble: 6 × 5
##   FeedType    AnimalName AnimalTag Date                IntakeLb
##   <chr>       <chr>          <dbl> <dttm>                 <dbl>
## 1 1_low_adapt 3                  3 2022-03-02 00:00:00        0
## 2 1_low_adapt 3                  3 2022-03-03 00:00:00        0
## 3 1_low_adapt 3                  3 2022-03-04 00:00:00        0
## 4 1_low_adapt 3                  3 2022-03-05 00:00:00        0
## 5 1_low_adapt 3                  3 2022-03-06 00:00:00        0
## 6 1_low_adapt 3                  3 2022-03-07 00:00:00        0
```

```
head(Drymatter)
```

```
## # A tibble: 6 × 3
##   TrialPeriod   sample_numb DM_pct
##   <chr>               <dbl>  <dbl>
## 1 2_low_adapt             1   93.6
## 2 2_low_adapt             2   93.6
## 3 2_low_adapt             3   92.8
## 4 2_low_collect           1   93.1
## 5 2_low_collect           2   93.8
## 6 2_low_collect           3   93.4
```

```
head(Weights)
```

```
## # A tibble: 6 × 3
##   AnimalTag Date                Weight
##       <dbl> <dttm>               <dbl>
## 1         3 2022-02-22 00:00:00     0
## 2       167 2022-02-22 00:00:00  1314.
## 3       195 2022-02-22 00:00:00     0
## 4       215 2022-02-22 00:00:00  1283.
## 5       224 2022-02-22 00:00:00  1285.
## 6       235 2022-02-22 00:00:00  1317.
```

```
head(Enteric)
```

```
## # A tibble: 6 × 23
##   `RFID Number`  `Farm Number` `Unit ID` `Start Time`        `End Time`
##   <chr>          <chr>             <dbl> <dttm>              <dttm>
## 1 0000000009820… 000000000982…       298 2022-03-25 19:03:07 2022-03-25 19:10:59
## 2 0000000009820… 000000000982…       298 2022-03-27 09:49:51 2022-03-27 10:05:05
## 3 0000000009820… 000000000982…       298 2022-03-27 12:03:49 2022-03-27 12:10:25
## 4 0000000009820… 000000000982…       298 2022-03-27 14:10:34 2022-03-27 14:16:19
## 5 0000000009820… 000000000982…       298 2022-03-28 01:45:09 2022-03-28 01:54:11
## 6 0000000009820… 000000000982…       298 2022-03-28 04:27:09 2022-03-28 04:32:49
## # ℹ 18 more variables: `Total Time with Good Data` <dttm>,
## #   `Hour of the Day` <dbl>, `Time of day bin` <lgl>, `CO2 (g/d)` <dbl>,
## #   `CH4 (g/d)` <dbl>, `O2 (g/d)` <dbl>, `Airflow (L/s)` <dbl>,
## #   `Airflow Cf` <dbl>, `Wind Sp` <dbl>, `Wind Dir` <dbl>, `Wind Cf` <dbl>,
## #   `midpoint >= 1 hour since last baseline (TRUE/FALSE)` <lgl>,
## #   `midpoint >= 1 hour until next baseline (TRUE/FALSE)` <chr>,
## #   `event was interrupted` <lgl>, `interrupting tag(s)` <chr>, …
```

```
colnames(Intake)
```

```
## [1] "FeedType"   "AnimalName" "AnimalTag"  "Date"       "IntakeLb"
```

```
colnames(Drymatter)
```

```
## [1] "TrialPeriod" "sample_numb" "DM_pct"
```

```
colnames(Weights)
```

```
## [1] "AnimalTag" "Date"      "Weight"
```

```
colnames(Enteric)
```

```
##  [1] "RFID Number"
##  [2] "Farm Number"
##  [3] "Unit ID"
##  [4] "Start Time"
##  [5] "End Time"
##  [6] "Total Time with Good Data"
##  [7] "Hour of the Day"
##  [8] "Time of day bin"
##  [9] "CO2 (g/d)"
## [10] "CH4 (g/d)"
## [11] "O2 (g/d)"
## [12] "Airflow (L/s)"
## [13] "Airflow Cf"
## [14] "Wind Sp"
## [15] "Wind Dir"
## [16] "Wind Cf"
## [17] "midpoint >= 1 hour since last baseline (TRUE/FALSE)"
## [18] "midpoint >= 1 hour until next baseline (TRUE/FALSE)"
## [19] "event was interrupted"
## [20] "interrupting tag(s)"
## [21] "stddev of last hour backgrounds"
## [22] "Average Temperature Celsius Gas Temp (O2)"
## [23] "Average Temperature Celsius In Pipe"
```

```
#Using head and colnames ee see that FeedType and TrialPeriod are the different but mean the sam
e thing.
#The code below makes them consistent by selecting the column and reassigning a new name "Trial_
Period."
colnames(Intake)[1]<- "Trial_Period"
colnames(Drymatter)[1]<- "Trial_Period"

#Drop "AnimalName" from the Intake dataframe because it is the same as AnimalTag
#and is not needed.

# Drop a column by name
#If this does not work then Restart R
Intake <- Intake %>% select(-AnimalName)

#Now lets change the Trial_Period to cleaner names and reorder them for Intake then for Forage
#data frames.
feedtype_rename <- c(
  "pre trail"     = "Pre_Trial",
  "1_low_adapt"   = "G2_Adapt_1",
  "1_low_collect" = "G2_Collect_1",
  "2_low_adapt"   = "G2_Adapt_2",
  "2_low_collect" = "G2_Collect_2",
  "high_adapt"    = "G1_Adapt",
  "high_collect"  = "G1_Collect"
)

#Rename and reorder the Intake data frame

Intake <- Intake %>%
  mutate(
    Trial_Period = dplyr::recode(Trial_Period,
                              "pre trail"     = "Pre_Trial",
                              "1_low_adapt"   = "G2_Adapt_1",
                              "1_low_collect" = "G2_Collect_1",
                              "2_low_adapt"   = "G2_Adapt_2",
                              "2_low_collect" = "G2_Collect_2",
                              "high_adapt"    = "G1_Adapt",
                              "high_collect"  = "G1_Collect"
    ),
    Trial_Period = factor(Trial_Period, levels = c(
      "Pre_Trial", "G2_Adapt_1", "G2_Collect_1",
      "G1_Adapt", "G1_Collect", "G2_Adapt_2", "G2_Collect_2"
    ))
  )


#Rename and reorder the Drymatter data frame

Drymatter <- Drymatter %>%
  mutate(
    Trial_Period = dplyr::recode(Trial_Period,
                                  "pre trail"     = "Pre_Trial",
```

```
                                   "1_low_adapt"   = "G2_Adapt_1",
                                   "1_low_collect" = "G2_Collect_1",
                                   "2_low_adapt"   = "G2_Adapt_2",
                                   "2_low_collect" = "G2_Collect_2",
                                   "high_adapt"    = "G1_Adapt",
                                   "high_collect"  = "G1_Collect"
    ),
    Trial_Period = factor(Trial_Period, levels = c(
      "Pre_Trial", "G2_Adapt_1", "G2_Collect_1",
      "G1_Adapt", "G1_Collect", "G2_Adapt_2", "G2_Collect_2"
    ))
  )

#Check if data frames were reordered
levels(Intake$Trial_Period)
```

```
## [1] "Pre_Trial"    "G2_Adapt_1"   "G2_Collect_1" "G1_Adapt"     "G1_Collect"
## [6] "G2_Adapt_2"   "G2_Collect_2"
```

```
levels(Drymatter$Trial_Period)
```

```
## [1] "Pre_Trial"    "G2_Adapt_1"   "G2_Collect_1" "G1_Adapt"     "G1_Collect"
## [6] "G2_Adapt_2"   "G2_Collect_2"
```

4. Incorporate Dry Matter Data

Average dry matter percentage by period (i.e., average sub-samples) and then merge into the Intake data frame. Create a dry matter intake column (DMI) using as fed intake and percent dry matter (DM_pct).

```
Drymatter_avg <- Drymatter %>%
  group_by(Trial_Period) %>%
  summarise(DM_pct = mean(DM_pct, na.rm = TRUE))

#Merge Data frames (Intake and Drymatter)
Intake_merged <- Intake %>%
  left_join(Drymatter_avg, by = "Trial_Period")

#Now we have 1480 obs with 5 variables in the Intake_merged data frame. The dry matter fraction
will be used to convert as fed to a DM basis and a new column called "DMI" is added.
Intake_merged$DMI = Intake_merged$IntakeLb * (Intake_merged$DM_pct/100)
```

5. Merge Weight Data

Now feed intake is reported on a dry matter basis we can calculate DMI as a percentage of body weight. The scale weight called Weights data frame is used. We see from using colnames() that the Weights data frame has the correct column heading of AnimalTag, which will be used to merge weight data into the Intake data frame.

```
colnames(Weights)
```

```
## [1] "AnimalTag" "Date"      "Weight"
```

```
Intake_Weight_Merged <- Intake_merged %>%
  left_join(Weights, by = c("AnimalTag", "Date"))

#Weight has successfully been integrated with the Intake and Forage data.
#Percent DMI of Body Weight can be estimated and added to the dataframe
#which we will use to remove outliers.

Intake_Weight_Merged$DMI_pct = ((Intake_Weight_Merged$DMI/Intake_Weight_Merged$Weight)* 100)

#The DMI_pct column has been added. NA and inf values need to be removed.
Intake_Weight_Merged <- Intake_Weight_Merged %>%
  mutate(DMI_pct = ifelse(is.na(DMI_pct) | is.infinite(DMI_pct), 0, DMI_pct))

#Confirm undesired values were removed.
summary(Intake_Weight_Merged$DMI_pct)
```

```
##    Min. 1st Qu.  Median    Mean 3rd Qu.    Max.
##   0.000   0.000   1.322   1.275   2.349   4.697
```

```
any(is.na(Intake_Weight_Merged$DMI_pct))       # should be FALSE
```

```
## [1] FALSE
```

```
any(is.infinite(Intake_Weight_Merged$DMI_pct))  # should be FALSE
```

```
## [1] FALSE
```

6. Dry Matter Intake Outlier Removal

Get rid outliers in DMI that we know are within a biologically acceptable range (though these bounds may be expanded). Note the current dataframe has 1480 obs with 8 variables and is reduced to 308 observations. These 308 observations will constrain the enteric data after it is merged.

```
Intake_Weight_Merged = subset(Intake_Weight_Merged, DMI_pct < 2.5)
Intake_Weight_Merged= subset(Intake_Weight_Merged, DMI_pct > 1.8)
```

##7. Process GreenFeed Data

Bring in enteric emissions and oxygen consumption data from GreenFeed files.

```
#Start Time is converted to a date.
Enteric$`Start Time`= as.Date(Enteric$`Start Time`)

#Each gas is brought in from the same file individually and aggregated by date.
average_CH4 = aggregate(Enteric$`CH4 (g/d)`, by = list (Enteric$`Start Time`, Enteric$`RFID Numb
er` ),FUN = mean)
colnames(average_CH4)= c("Date", "AnimalName", "Daily_Avg_CH4")

average_CO2 = aggregate(Enteric$`CO2 (g/d)`, by = list (Enteric$`Start Time`, Enteric$`RFID Numb
er` ),FUN = mean)
colnames(average_CO2)= c("Date", "AnimalName", "Daily_Avg_CO2")

#Average O2
average_O2 = aggregate(Enteric$`O2 (g/d)`, by = list (Enteric$`Start Time`, Enteric$`RFID Number
` ),FUN = mean)
colnames(average_O2)= c("Date", "AnimalName", "Daily_Avg_O2")
```

8. Merge Gas Data

Merge individual gas data frames into the Intake_Weight_Merged data frame.

```
# Ensure AnimalTag is consistent across data frames to be able to merge.
average_CH4 <- average_CH4 %>% rename(AnimalTag = AnimalName)
average_CO2 <- average_CO2 %>% rename(AnimalTag = AnimalName)
average_O2  <- average_O2 %>% rename(AnimalTag = AnimalName)

# Merge all emissions into a single data frame.
emissions_merged <- average_CH4 %>%
  full_join(average_CO2, by = c("AnimalTag", "Date")) %>%
  full_join(average_O2, by = c("AnimalTag", "Date"))

#Extract last 3 characters from AnimalName in emissions data to create AnimalTag
#that is consistent with other dataframes. I.e., the last three of the RFID tag.
emissions_merged <- emissions_merged %>%
  mutate(
    AnimalTag = substr(as.character(AnimalTag), nchar(AnimalTag) - 2, nchar(AnimalTag))
  )

# Ensure AnimalTag is character in all data frames
Intake_Weight_Merged <- Intake_Weight_Merged %>%
  mutate(AnimalTag = as.character(AnimalTag))

emissions_merged <- emissions_merged %>%
  mutate(AnimalTag = as.character(AnimalTag))

# Merge
Intake_Weight_Emissions <- Intake_Weight_Merged %>%
  left_join(emissions_merged, by = c("AnimalTag", "Date"))

# View result
summary(Intake_Weight_Emissions)
```

```
##       Trial_Period  AnimalTag              Date                    
##  Pre_Trial   : 0    Length:308         Min.   :2022-02-22 00:00:00.00  
##  G2_Adapt_1  :37    Class :character   1st Qu.:2022-03-16 00:00:00.00  
##  G2_Collect_1:69    Mode  :character   Median :2022-04-05 00:00:00.00  
##  G1_Adapt    :45                       Mean   :2022-04-05 01:14:48.30  
##  G1_Collect  :63                       3rd Qu.:2022-04-25 06:00:00.00  
##  G2_Adapt_2  :49                       Max.   :2022-05-17 00:00:00.00  
##  G2_Collect_2:45                                                       
##     IntakeLb         DM_pct           DMI            Weight        DMI_pct     
##  Min.   :21.83   Min.   :93.30   Min.   :20.77   Min.   :1113   Min.   :1.801  
##  1st Qu.:28.48   1st Qu.:93.33   1st Qu.:27.09   1st Qu.:1306   1st Qu.:2.004  
##  Median :31.82   Median :93.43   Median :30.12   Median :1382   Median :2.158  
##  Mean   :32.21   Mean   :94.40   Mean   :30.39   Mean   :1403   Mean   :2.164  
##  3rd Qu.:35.05   3rd Qu.:95.13   3rd Qu.:32.88   3rd Qu.:1513   3rd Qu.:2.339  
##  Max.   :46.60   Max.   :96.93   Max.   :43.49   Max.   :1792   Max.   :2.499  
##                                                                                
##  Daily_Avg_CH4    Daily_Avg_CO2    Daily_Avg_O2 
##  Min.   : 40.04   Min.   : 2900   Min.   :1843  
##  1st Qu.:202.32   1st Qu.: 6568   1st Qu.:4794  
##  Median :258.48   Median : 7581   Median :5451  
##  Mean   :252.14   Mean   : 7555   Mean   :5372  
##  3rd Qu.:301.15   3rd Qu.: 8766   3rd Qu.:6122  
##  Max.   :442.67   Max.   :11320   Max.   :8241  
##  NA's   :172      NA's   :172     NA's   :172   
```

9. Subset Fully Merged Data

Now we have a data frame with all the data merged we can subset the data for animals that were present during the entire trial. Note that data loss can happen during merges so it is prudent to check subsets of data from original data frames prior to merging (details not included in this tutorial).

```
#Cow RFID tag numbers (last 3)
keep_tags <- c(167, 224, 235, 263, 313, 336, 387)

# Subset the data frame
complete_data <- Intake_Weight_Emissions[Intake_Weight_Merged$AnimalTag %in% keep_tags, ]

#Rename complete_data so that we don't override the completed dataset during next
#coding sections.

clean_data <- complete_data
# 1) Daily herd-level averages
herd_daily_avg <- clean_data %>%
  group_by(Trial_Period, Date) %>%
  summarise(
    herd_DMI  = mean(DMI,              na.rm = TRUE),
    herd_CH4  = mean(Daily_Avg_CH4,    na.rm = TRUE),
    herd_CO2  = mean(Daily_Avg_CO2,    na.rm = TRUE),
    herd_O2   = mean(Daily_Avg_O2,     na.rm = TRUE),
    .groups = "drop"
  )

# 2) Period-level herd averages (averaged over time within each Trial_Period)
herd_period_avg <- herd_daily_avg %>%
  group_by(Trial_Period) %>%
  summarise(
    avg_DMI  = mean(herd_DMI,  na.rm = TRUE),
    avg_CH4  = mean(herd_CH4,  na.rm = TRUE),
    avg_CO2  = mean(herd_CO2,  na.rm = TRUE),
    avg_O2   = mean(herd_O2,   na.rm = TRUE),
    .groups = "drop"
  )

# Inspect
print(herd_daily_avg)
```

```
## # A tibble: 75 × 6
##    Trial_Period Date                herd_DMI herd_CH4 herd_CO2 herd_O2
##    <fct>        <dttm>                 <dbl>    <dbl>    <dbl>   <dbl>
##  1 G2_Adapt_1   2022-02-22 00:00:00     30.3     NaN      NaN     NaN
##  2 G2_Adapt_1   2022-02-23 00:00:00     24.0     NaN      NaN     NaN
##  3 G2_Adapt_1   2022-02-24 00:00:00     32.2     205.    7303.   6470.
##  4 G2_Adapt_1   2022-02-28 00:00:00     37.3     243.    9123.   6525.
##  5 G2_Adapt_1   2022-03-01 00:00:00     23.8     NaN      NaN     NaN
##  6 G2_Adapt_1   2022-03-02 00:00:00     27.8     149.    6671.   4904.
##  7 G2_Adapt_1   2022-03-03 00:00:00     27.9     138.    4739.   3535.
##  8 G2_Adapt_1   2022-03-04 00:00:00     28.9     223.    6989.   5539.
##  9 G2_Adapt_1   2022-03-05 00:00:00     24.8     NaN      NaN     NaN
## 10 G2_Adapt_1   2022-03-07 00:00:00     25.6     NaN      NaN     NaN
## # ℹ 65 more rows
```

```
print(herd_period_avg)
```

```
## # A tibble: 6 × 5
##   Trial_Period avg_DMI avg_CH4 avg_CO2 avg_O2
##   <fct>          <dbl>   <dbl>   <dbl>  <dbl>
## 1 G2_Adapt_1      28.3    191.   6965.  5394.
## 2 G2_Collect_1    29.3    291.   8457.  5685.
## 3 G1_Adapt        29.3    192.   6065.  4390.
## 4 G1_Collect      28.6    198.   6421.  4812.
## 5 G2_Adapt_2      33.2    284.   7967.  5632.
## 6 G2_Collect_2    33.2    276.   8063.  5787.
```

```
clean_data_no<-na.omit(clean_data)
```

10. Descriptive Statistics

Perform descriptive statistics on all periods. The trial had an additional G2 adaptation and collection period but only the last two collection periods (G1_Collect and G2_Collect_2) were used due to low animal use of the GreenFeed.

```
# Define color scheme for treatments.
feed_colors <- c(
  "G2_Adapt_1"    = "#56b400",
  "G2_Collect_1"  = "#009e73",
  "G1_Adapt"      = "#0072b2",
  "G1_Collect"    = "#9467bd",
  "G2_Adapt_2"    = "#e377c2",
  "G2_Collect_2"  = "#f781bf"
)

# Ensure Trial_Period is a factor with all levels defined.
clean_data <- clean_data %>%
  mutate(
    Trial_Period = factor(Trial_Period, levels = names(feed_colors)),
    DMI_kg = DMI * 0.453592,
    Weight_kg = Weight * 0.453592
  )

# Summarise and preserve all levels.
summary_means <- clean_data %>%
  group_by(Trial_Period) %>%
  summarise(
    DMI_kg = mean(DMI_kg, na.rm = TRUE),
    Weight_kg = mean(Weight_kg, na.rm = TRUE),
    CH4 = mean(Daily_Avg_CH4, na.rm = TRUE),
    CO2 = mean(Daily_Avg_CO2, na.rm = TRUE),
    O2 = mean(Daily_Avg_O2, na.rm = TRUE),
    .groups = "drop"
  ) %>%

# Ensure all periods appear, even if NA.
  complete(Trial_Period = factor(levels(clean_data$Trial_Period), levels = levels(clean_data$Tri
al_Period))) %>%
# Replace any remaining NAs with 0 for plotting
  mutate(across(where(is.numeric), ~ replace_na(., 0)))

plot_bar <- function(var) {
  y_label <- switch(var,
                    "DMI_kg"   = bquote("DMI (kg" / day * ")"),
                    "Weight_kg"= bquote("Weight (kg)"),
                    "CH4"      = bquote("CH"[4] ~ "(g/day)"),
                    "CO2"      = bquote("CO"[2] ~ "(g/day)"),
                    "O2"       = bquote("O"[2] ~ "(g/day)")
  )

  title_label <- switch(var,
                        "DMI_kg"   = "Average DMI by Trial Period",
                        "Weight_kg"= "Average Weight by Trial Period",
                        "CH4"      = expression("Average CH"[4] * " by Trial Period"),
                        "CO2"      = expression("Average CO"[2] * " by Trial Period"),
                        "O2"       = expression("Average O"[2] * " by Trial Period")
  )
```

```
  ggplot(summary_means, aes(x = Trial_Period, y = .data[[var]], fill = Trial_Period)) +
    geom_bar(stat = "identity") +
    scale_fill_manual(values = feed_colors) +
    ggtitle(title_label) +
    labs(x = "Trial Period", y = y_label) +
    theme_minimal() +
    theme(axis.text.x = element_text(angle = 45, hjust = 1))
}
plot_bar("DMI_kg")
```

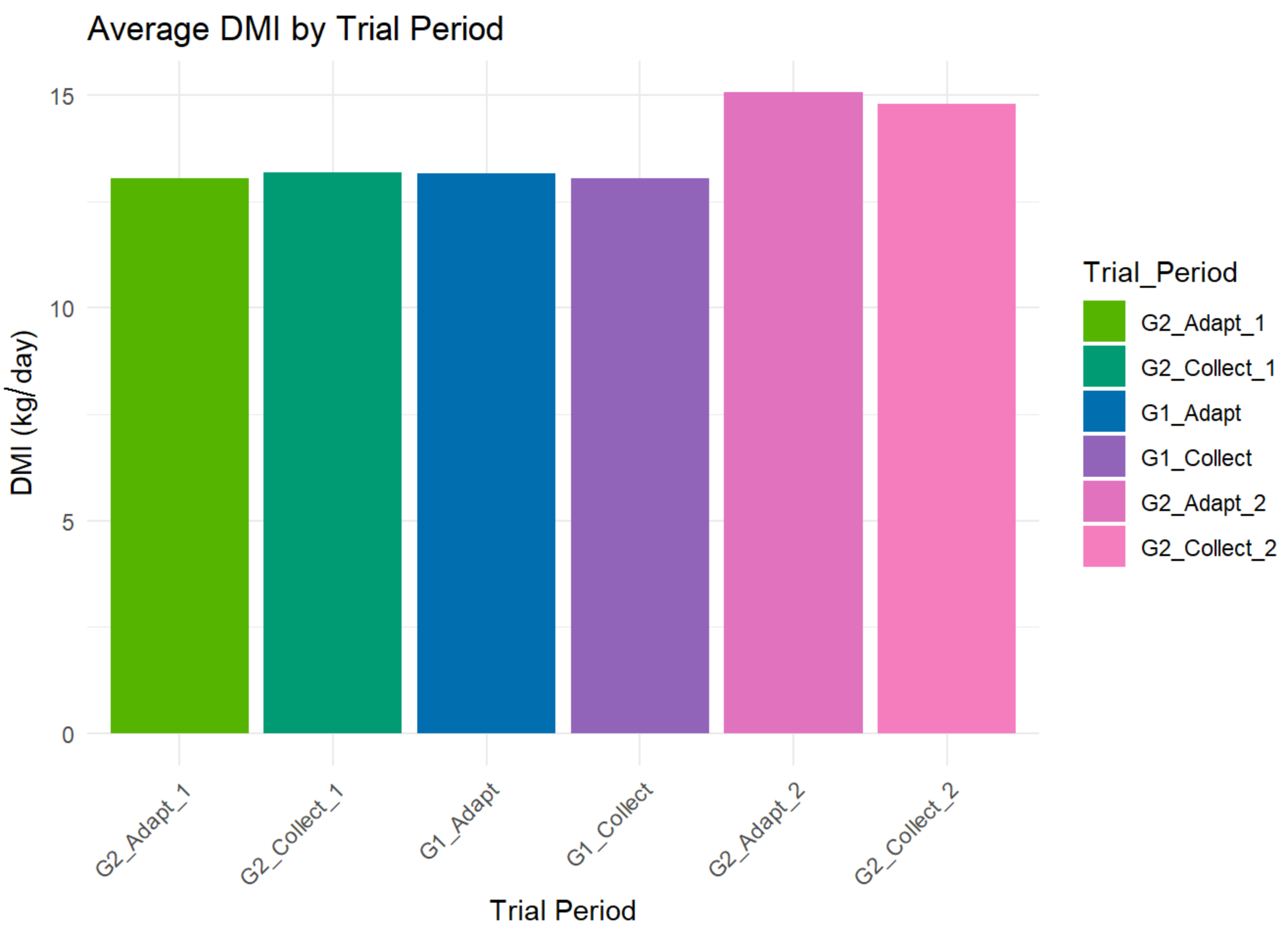


```
plot_bar("CH4")
```

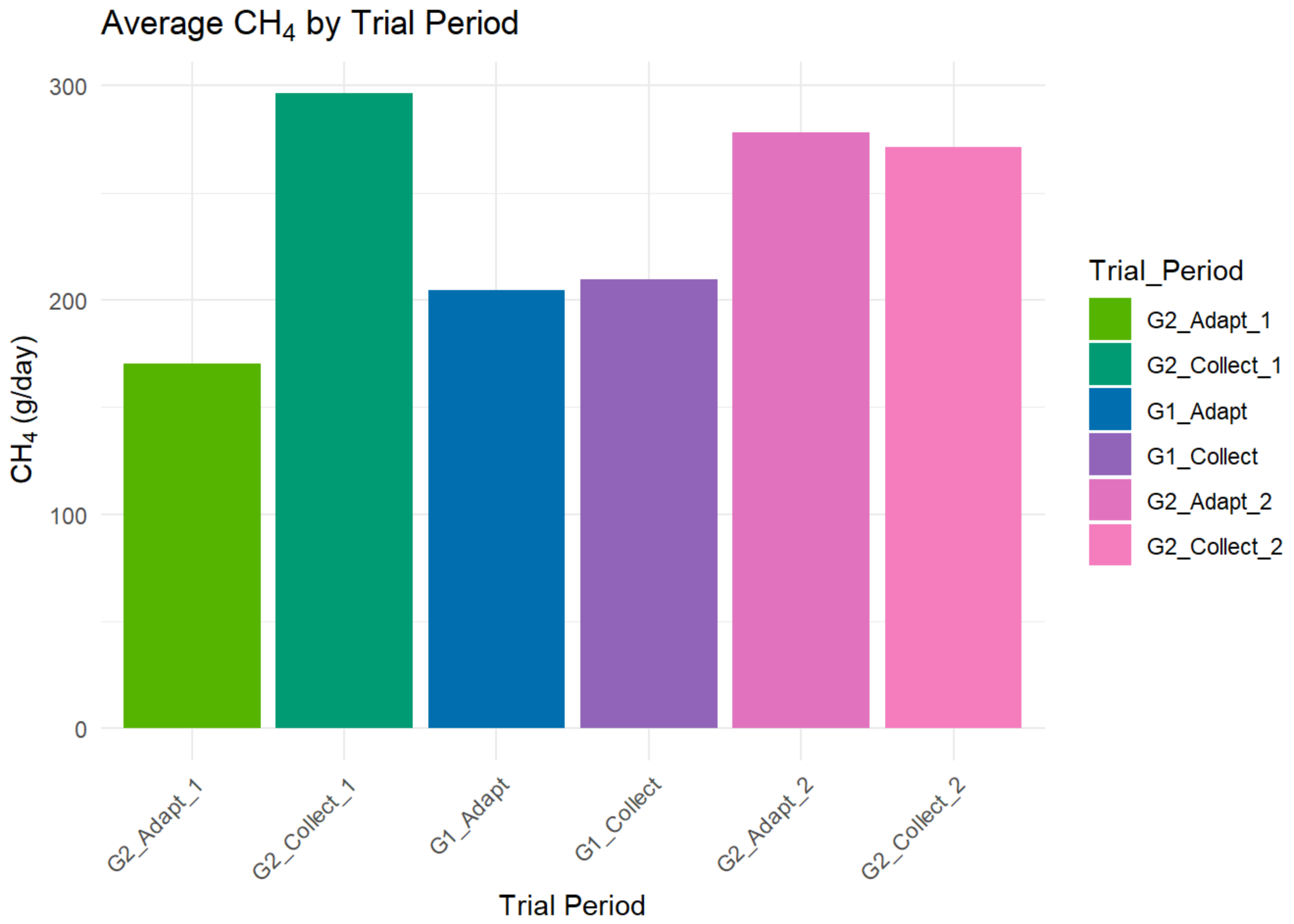
Average CH_4 by Trial Period
300
200
100
0
CH_4 (g/day)
G2_Adapt_1
G2_Collect_1
G1_Adapt
G1_Collect
G2_Adapt_2
G2_Collect_2
Trial Period
Trial_Period
G2_Adapt_1
G2_Collect_1
G1_Adapt
G1_Collect
G2_Adapt_2
G2_Collect_2

```
plot_bar("CO2")
```

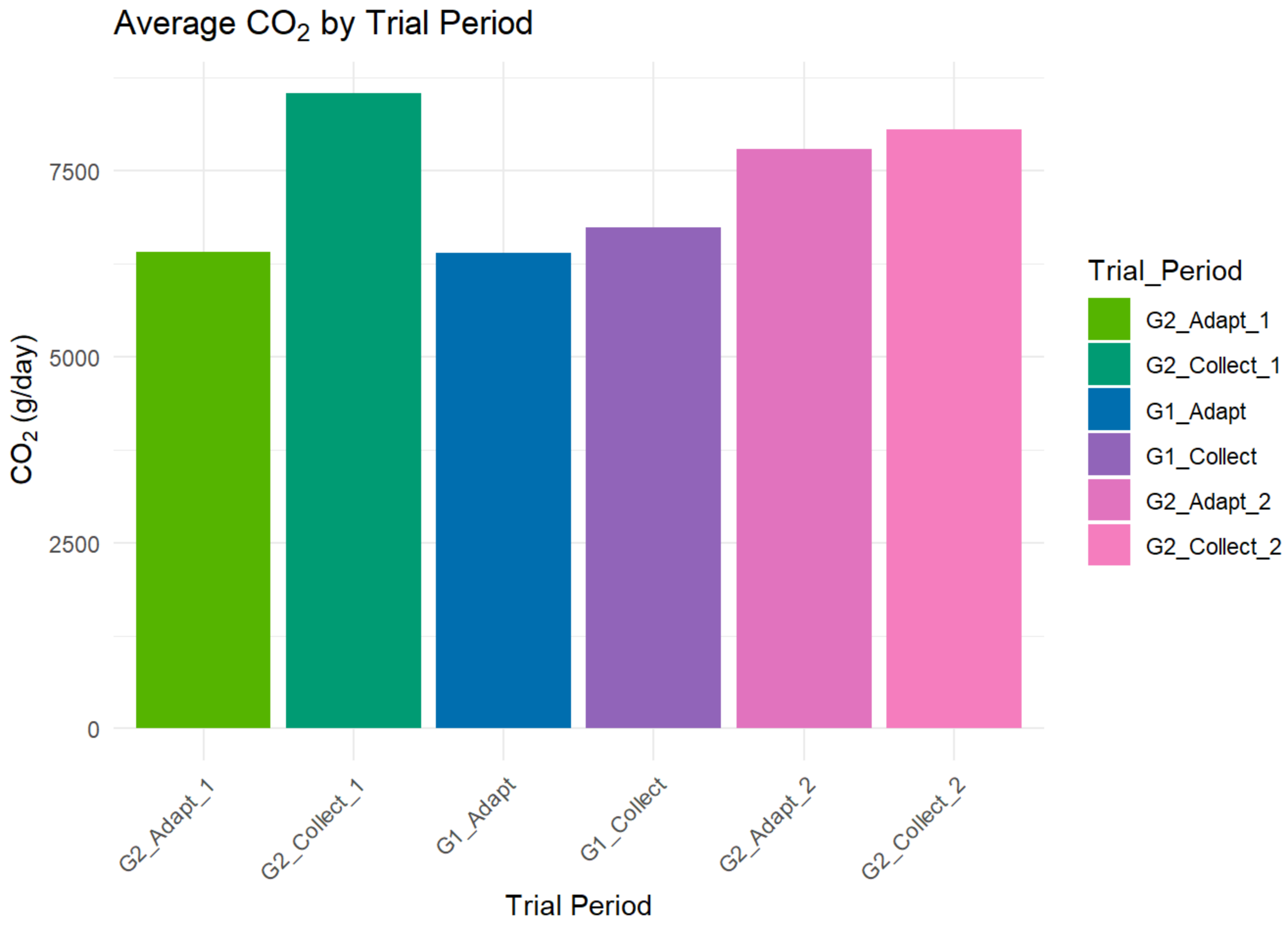
Average CO_2 by Trial Period
7500
5000
2500
0
CO_2 (g/day)
G2_Adapt_1
G2_Collect_1
G1_Adapt
G1_Collect
G2_Adapt_2
G2_Collect_2
Trial Period
Trial_Period
G2_Adapt_1
G2_Collect_1
G1_Adapt
G1_Collect
G2_Adapt_2
G2_Collect_2

```
plot_bar("O2")
```

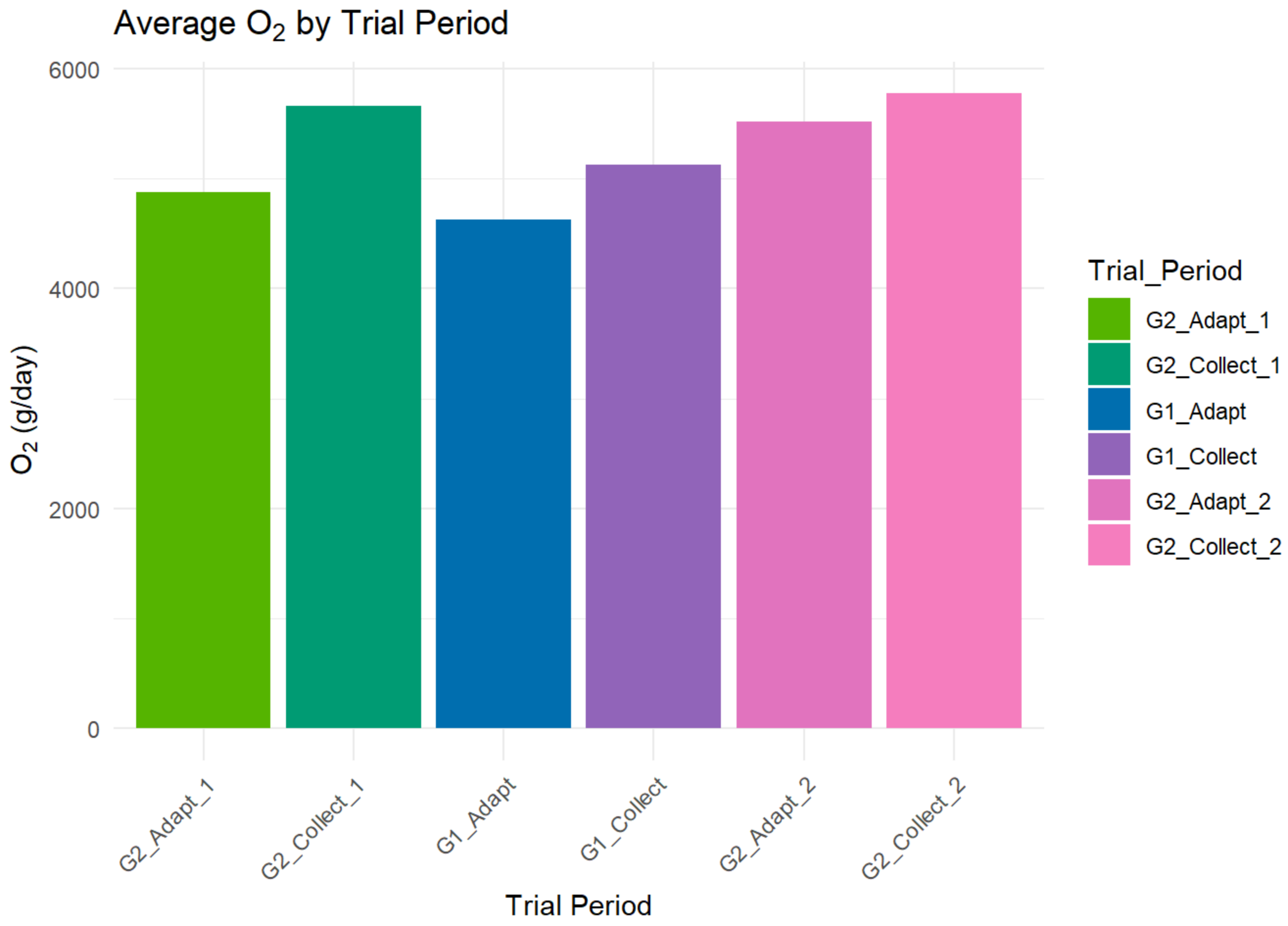

11. Remove Outliers

Here two outlier removal methods are presented: 1) Interquartile Range (IRQ) and 2) two-standard deviations from the mean. In the current study we use IQR but the user can adjust parameters of the IQR or switch to the standard deviation method using the “remove_outliers_sd” data frame instead of the “remove_outliers” data frame.

```r
# Define a function to remove outliers using 0.75 * IQR.
remove_outliers <- function(df, column) {
  q <- quantile(df[[column]], probs = c(0.25, 0.75), na.rm = TRUE)
  iqr <- IQR(df[[column]], na.rm = TRUE)
  lower <- q[1] - 0.75 * iqr
  upper <- q[2] + 0.75 * iqr
  df[df[[column]] > lower & df[[column]] < upper, ]
}
#Create a function to remove outliers using 2 standard deviations.
remove_outliers_sd <- function(df, column) {
  mean_val <- mean(df[[column]], na.rm = TRUE)
  sd_val <- sd(df[[column]], na.rm = TRUE)
  lower <- mean_val - 2 * sd_val
  upper <- mean_val + 2 * sd_val
  df[df[[column]] >= lower & df[[column]] <= upper, ]
}
# Remove outliers for each gas. Choose remove_outliers for IQR or
#choose remove_outliers_sd for less strict outlier removal.
#The IQR data frames are used for the rest of the code and the
#other can be deployed for further data exploration and analysis.

# Apply to CH4
clean_dataCH4 <- remove_outliers(clean_data, "Daily_Avg_CH4")

# Apply to CO2
clean_dataCO2 <- remove_outliers(clean_data, "Daily_Avg_CO2")

# Apply to O2
clean_dataO2 <- remove_outliers(clean_data, "Daily_Avg_O2")

#clean_data has no extreme outliers for CH4, CO2, or O2 using the IQR method.

# Subset to only G2_Collect_2 and G1_Collect for IQR.
subset_data <- clean_data %>%
  filter(Trial_Period %in% c("G2_Collect_2", "G1_Collect"))
subset_data_clean <- subset_data %>%
  drop_na(Daily_Avg_CH4, Daily_Avg_CO2, Daily_Avg_O2)

# Ensure Trial_Period is a factor with the correct chronological order.
subset_data_clean$Trial_Period <- factor(subset_data_clean$Trial_Period, levels = c("G1_Collec
t", "G2_Collect_2"))

#Apply two standard deviations for outlier removal (optional)
#Normally is is less strict than IQR but this method reduces observations to 41
#compared to 44 using the IQR method.

#Remove outliers for each gas from original clean_data
clean2_dataCH4 <- remove_outliers_sd(clean_data, "Daily_Avg_CH4")
clean2_dataCO2 <- remove_outliers_sd(clean_data, "Daily_Avg_CO2")
clean2_dataO2  <- remove_outliers_sd(clean_data, "Daily_Avg_O2")

#Keep only observations that are present in all cleaned data sets
```

```
# (i.e., no outliers in CH4, CO2, or O2)

clean_data_filtered2 <- clean_data %>%
  semi_join(clean2_dataCH4, by = c("AnimalTag", "Date")) %>%
  semi_join(clean2_dataCO2, by = c("AnimalTag", "Date")) %>%
  semi_join(clean2_dataO2,  by = c("AnimalTag", "Date"))

#Repeat if needed for alternative outlier removal:
#Subset to only the desired trial periods.
subset_data2 <- clean_data_filtered2 %>%
  filter(Trial_Period %in% c("G1_Collect", "G2_Collect_2"))

#Drop any remaining NA values
subset_data_clean2 <- subset_data2 %>%
  drop_na(Daily_Avg_CH4, Daily_Avg_CO2, Daily_Avg_O2)

#Ensure correct factor level order
subset_data_clean2$Trial_Period <- factor(subset_data_clean2$Trial_Period, levels = c("G1_Collec
t", "G2_Collect_2"))
```

12. Descriptive statistics.

Run descriptive statistics and plots on subset data for collection periods (G1 and G2).

```
clean_data <- subset_data_clean %>%
  mutate(
    Trial_Period = factor(Trial_Period, levels = names(feed_colors)),
  )

#Summarise and preserve all levels
summary_means <- clean_data %>%
  group_by(Trial_Period) %>%
  summarise(
    DMI_kg = mean(DMI_kg, na.rm = TRUE),
    Weight_kg = mean(Weight_kg, na.rm = TRUE),
    CH4 = mean(Daily_Avg_CH4, na.rm = TRUE),
    CO2 = mean(Daily_Avg_CO2, na.rm = TRUE),
    O2 = mean(Daily_Avg_O2, na.rm = TRUE),
    .groups = "drop"
  ) %>%

# Ensure all periods appear, even if NA
  complete(Trial_Period = factor(levels(clean_data$Trial_Period), levels = levels(clean_data$Tri
al_Period))) %>%
# Replace any remaining NAs with 0 for plotting
  mutate(across(where(is.numeric), ~ replace_na(., 0)))

#Create plotting function.
plot_bar <- function(var) {
  y_label <- switch(var,
                    "DMI_kg"   = bquote("DMI (kg" / day * ")"),
                    "Weight_kg"= bquote("Weight (kg)"),
                    "CH4"      = bquote("CH"[4] ~ "(g/day)"),
                    "CO2"      = bquote("CO"[2] ~ "(g/day)"),
                    "O2"       = bquote("O"[2] ~ "(g/day)")
  )

  title_label <- switch(var,
                        "DMI_kg"   = "Average DMI by Trial Period",
                        "Weight_kg"= "Average Weight by Trial Period",
                        "CH4"      = expression("Average CH"[4] * " by Trial Period"),
                        "CO2"      = expression("Average CO"[2] * " by Trial Period"),
                        "O2"       = expression("Average O"[2] * " by Trial Period")
  )

  ggplot(summary_means, aes(x = Trial_Period, y = .data[[var]], fill = Trial_Period)) +
    geom_bar(stat = "identity") +
    scale_fill_manual(values = feed_colors) +
    ggtitle(title_label) +
    labs(x = "Trial Period", y = y_label) +
    theme_minimal() +
    theme(axis.text.x = element_text(angle = 45, hjust = 1))
}
```

```
#Create plots.
plot_bar("DMI_kg")
```

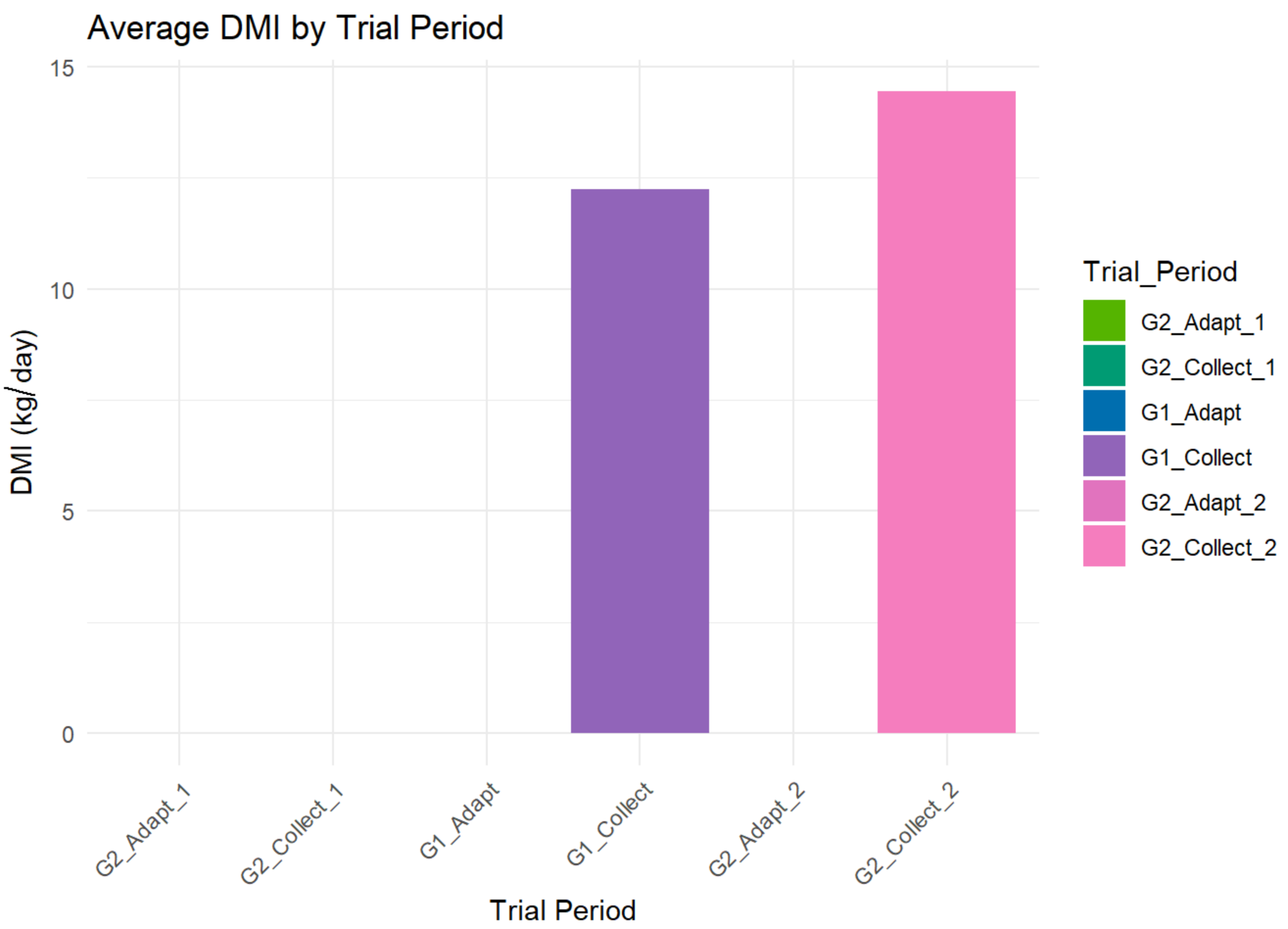


```
plot_bar("CH4")
```

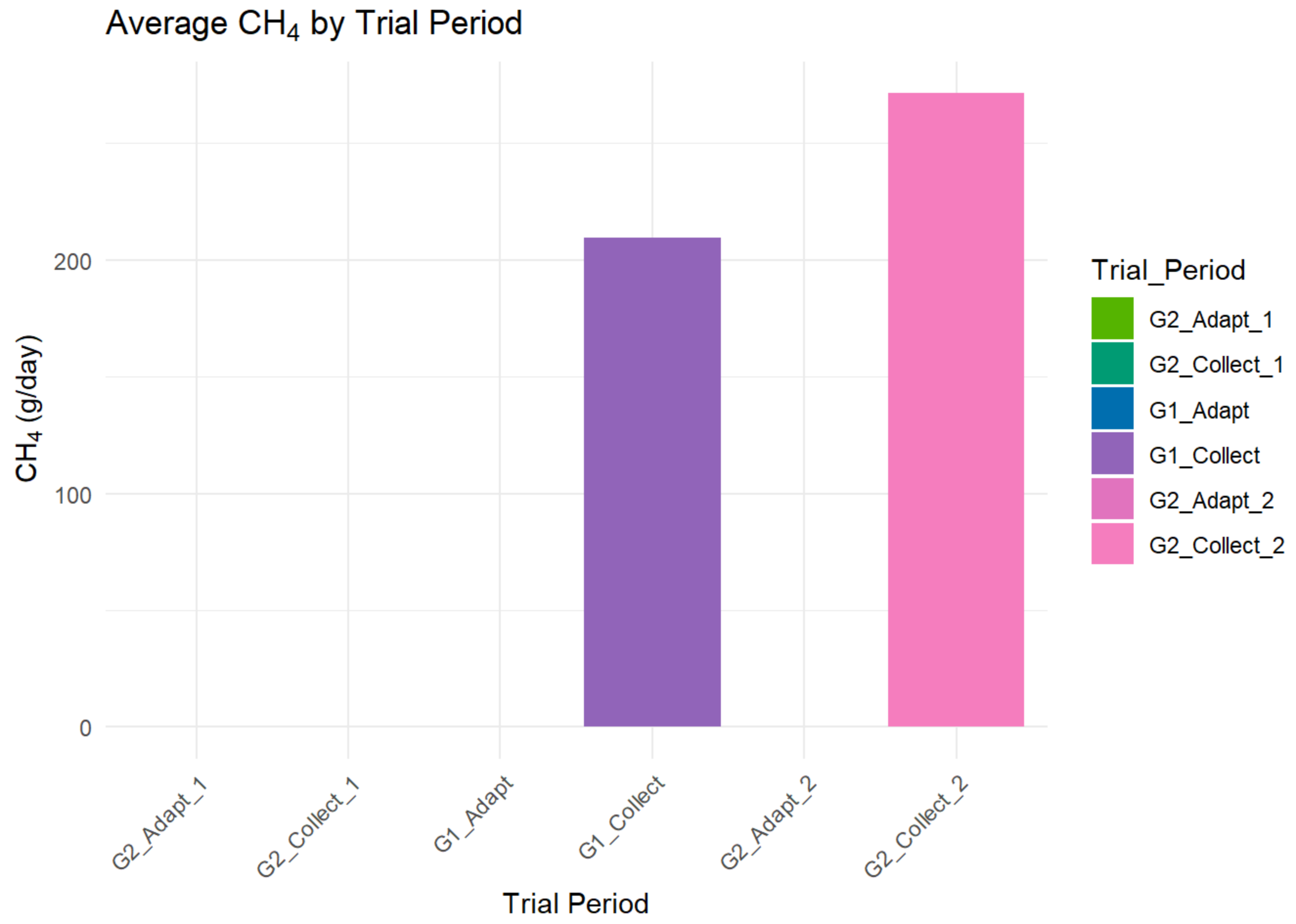
Average CH_4 by Trial Period
CH_4 (g/day)
200
100
0
G2_Adapt_1
G2_Collect_1
G1_Adapt
G1_Collect
G2_Adapt_2
G2_Collect_2
Trial Period
Trial_Period
G2_Adapt_1
G2_Collect_1
G1_Adapt
G1_Collect
G2_Adapt_2
G2_Collect_2

```
plot_bar("CO2")
```

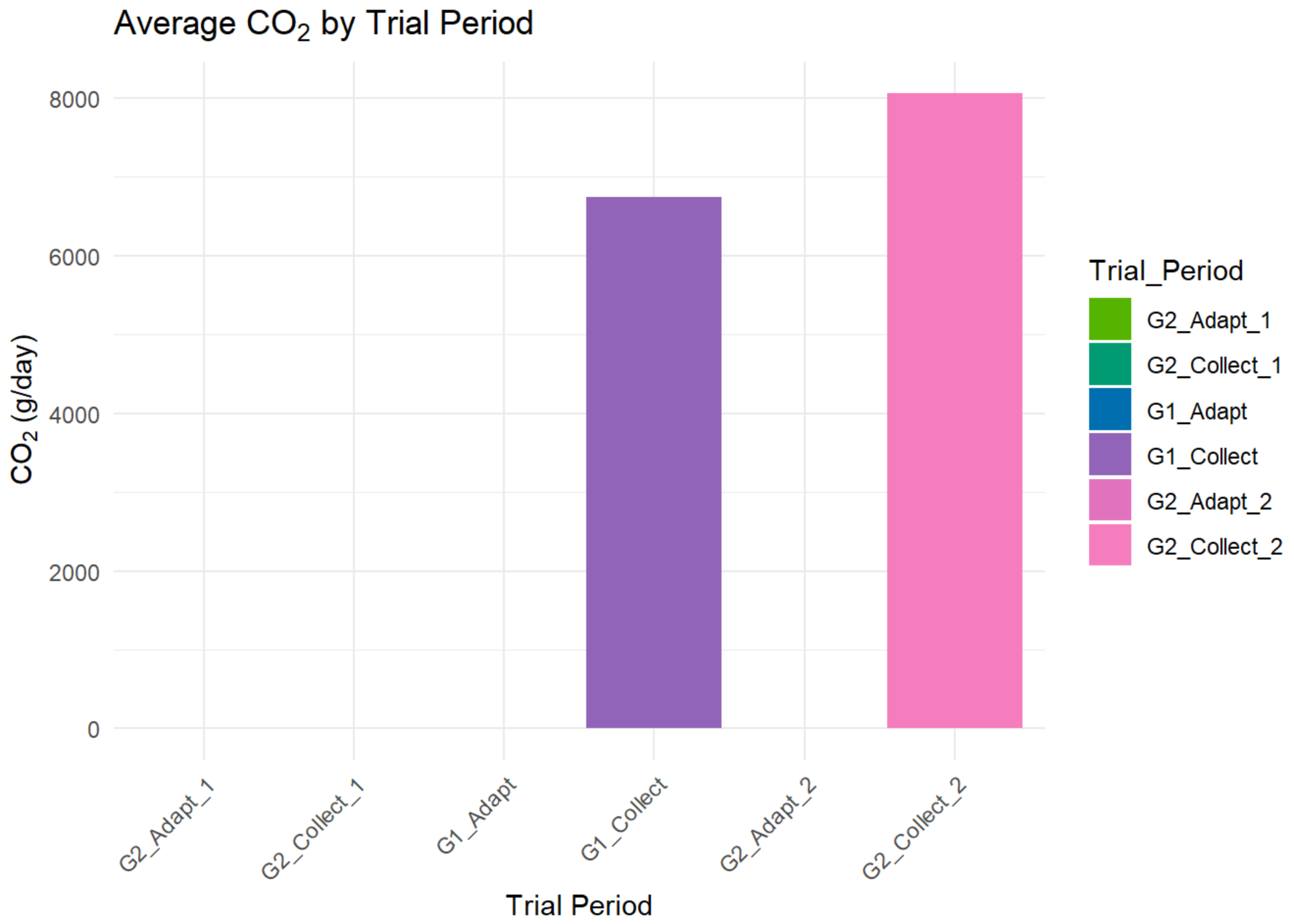
Average CO_2 by Trial Period
8000
6000
4000
2000
0
CO_2 (g/day)
G2_Adapt_1
G2_Collect_1
G1_Adapt
G1_Collect
G2_Adapt_2
G2_Collect_2
Trial Period
Trial_Period
G2_Adapt_1
G2_Collect_1
G1_Adapt
G1_Collect
G2_Adapt_2
G2_Collect_2

```
plot_bar("O2")
```

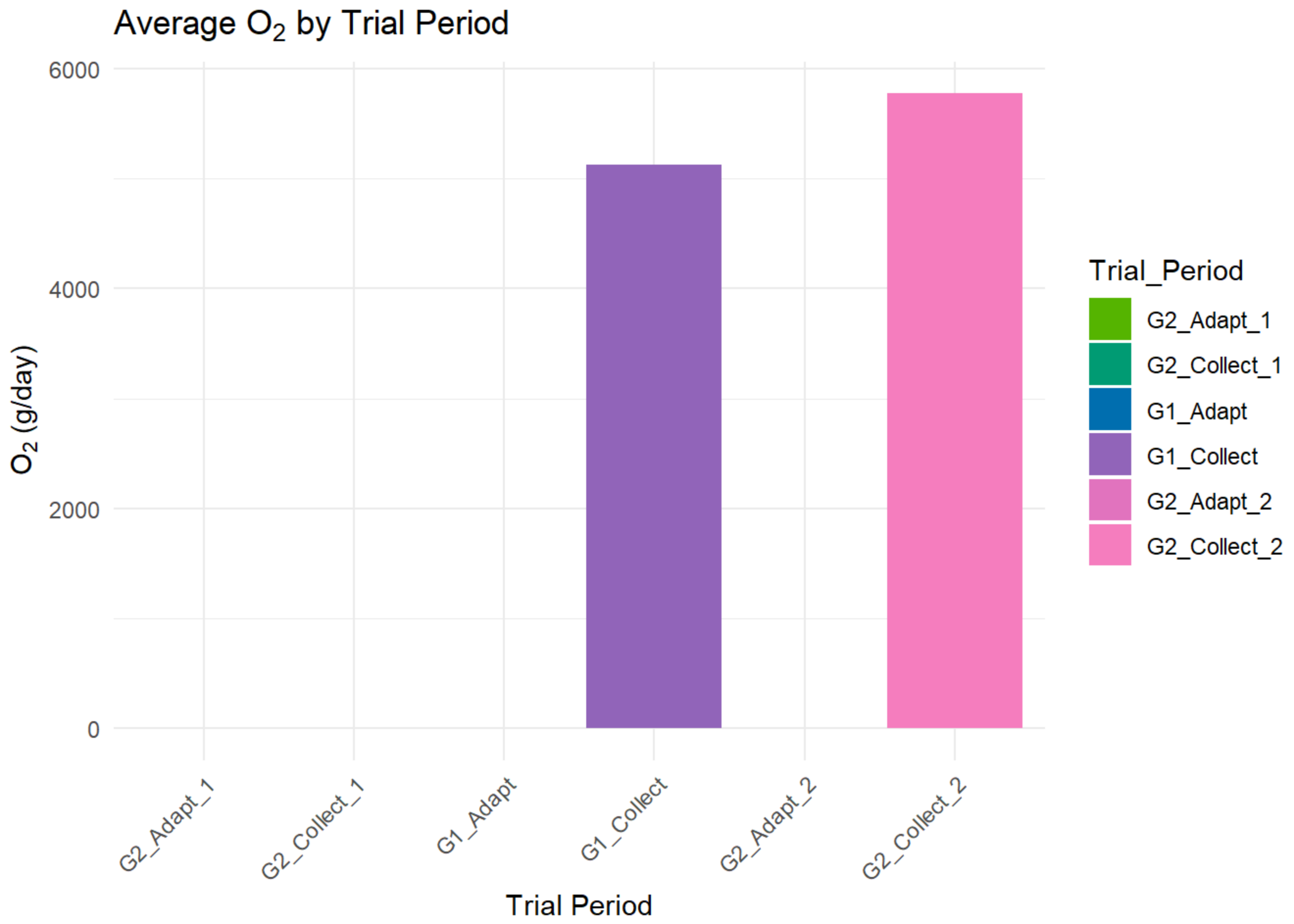


```
#Note that the other bars are not present because the subset was successful.
```

13. Descriptive stats grouped by Trial_Period.

Descriptive statistics include mean, standard deviation (sd), minimum (min), maximum (max), and sample size (n).

```
descriptive_stats <- subset_data_clean %>%
  filter(Trial_Period %in% c("G1_Collect", "G2_Collect_2")) %>%
  group_by(Trial_Period) %>%
  summarise(
    # DMI
    mean_DMI = mean(DMI, na.rm = TRUE),
    sd_DMI   = sd(DMI, na.rm = TRUE),
    min_DMI  = min(DMI, na.rm = TRUE),
    max_DMI  = max(DMI, na.rm = TRUE),
    n_DMI    = sum(!is.na(DMI)),

    # CH4
    mean_CH4 = mean(Daily_Avg_CH4, na.rm = TRUE),
    sd_CH4   = sd(Daily_Avg_CH4, na.rm = TRUE),
    min_CH4  = min(Daily_Avg_CH4, na.rm = TRUE),
    max_CH4  = max(Daily_Avg_CH4, na.rm = TRUE),
    n_CH4    = sum(!is.na(Daily_Avg_CH4)),

    # CO2
    mean_CO2 = mean(Daily_Avg_CO2, na.rm = TRUE),
    sd_CO2   = sd(Daily_Avg_CO2, na.rm = TRUE),
    min_CO2  = min(Daily_Avg_CO2, na.rm = TRUE),
    max_CO2  = max(Daily_Avg_CO2, na.rm = TRUE),
    n_CO2    = sum(!is.na(Daily_Avg_CO2)),

    # O2
    mean_O2 = mean(Daily_Avg_O2, na.rm = TRUE),
    sd_O2   = sd(Daily_Avg_O2, na.rm = TRUE),
    min_O2  = min(Daily_Avg_O2, na.rm = TRUE),
    max_O2  = max(Daily_Avg_O2, na.rm = TRUE),
    n_O2    = sum(!is.na(Daily_Avg_O2))
  )

descriptive_stats
```

```
## # A tibble: 2 × 21
##   Trial_Period mean_DMI sd_DMI min_DMI max_DMI n_DMI mean_CH4 sd_CH4 min_CH4
##   <fct>           <dbl>  <dbl>   <dbl>   <dbl> <int>    <dbl>  <dbl>   <dbl>
## 1 G1_Collect       27.0   3.98    21.1    34.0    18     210.   60.4    106.
## 2 G2_Collect_2     31.8   4.74    22.1    42.4    26     271.   65.3    126.
## # ℹ 12 more variables: max_CH4 <dbl>, n_CH4 <int>, mean_CO2 <dbl>,
## #   sd_CO2 <dbl>, min_CO2 <dbl>, max_CO2 <dbl>, n_CO2 <int>, mean_O2 <dbl>,
## #   sd_O2 <dbl>, min_O2 <dbl>, max_O2 <dbl>, n_O2 <int>
```

14. Run Mixed Model ANOVA

Assessing differences in DMI, CH_4, CO_2, and O_2 by trial period (G1 and G2).

```
# Fit mixed model
model_DMI <- lmer(DMI ~ Trial_Period + (1 | AnimalTag), data = subset_data_clean)
summary(model_DMI)
```

```
## Linear mixed model fit by REML. t-tests use Satterthwaite's method [
## lmerModLmerTest]
## Formula: DMI ~ Trial_Period + (1 | AnimalTag)
##    Data: subset_data_clean
##
## REML criterion at convergence: 228.3
##
## Scaled residuals:
##      Min       1Q   Median       3Q      Max
## -1.79316 -0.53223  0.09016  0.49855  1.83012
##
## Random effects:
##  Groups    Name        Variance Std.Dev.
##  AnimalTag (Intercept) 15.397   3.924
##  Residual               8.321   2.885
## Number of obs: 44, groups:  AnimalTag, 7
##
## Fixed effects:
##                         Estimate Std. Error     df t value Pr(>|t|)
## (Intercept)               28.874      1.677  7.305  17.213 3.53e-07 ***
## Trial_PeriodG2_Collect_2    2.883      1.023 38.566   2.819  0.00757 **
## ---
## Signif. codes:  0 '***' 0.001 '**' 0.01 '*' 0.05 '.' 0.1 ' ' 1
##
## Correlation of Fixed Effects:
##             (Intr)
## Trl_PG2_C_2 -0.359
```

```
# Get estimated marginal means
emm <- emmeans(model_DMI, ~ Trial_Period)
emm_df <- as.data.frame(emm)

# Convert from pounds to kilograms
emm_df$emmean_kg <- emm_df$emmean / 2.20462
emm_df$SE_kg <- emm_df$SE / 2.20462

# Assign labels (manually since only 2 groups)
emm_df$Label <- c("b", "a")  # adjust based on group means

# Plot
ggplot(emm_df, aes(x = Trial_Period, y = emmean_kg, fill = Trial_Period)) +
  geom_col() +
  geom_errorbar(aes(ymin = emmean_kg - SE_kg, ymax = emmean_kg + SE_kg), width = 0.5) +
  geom_text(
    aes(label = Label, y = emmean_kg + SE_kg + 0.5),
    fontface = "bold", size = 5
  ) +
  ylim(0, max(emm_df$emmean_kg + emm_df$SE_kg) + 1) +
  labs(x = "Trial Period", y = "Dry Matter Intake (kg/day)") +
  scale_fill_manual(values = c("yellow", "blue")) +
  theme_classic()
```

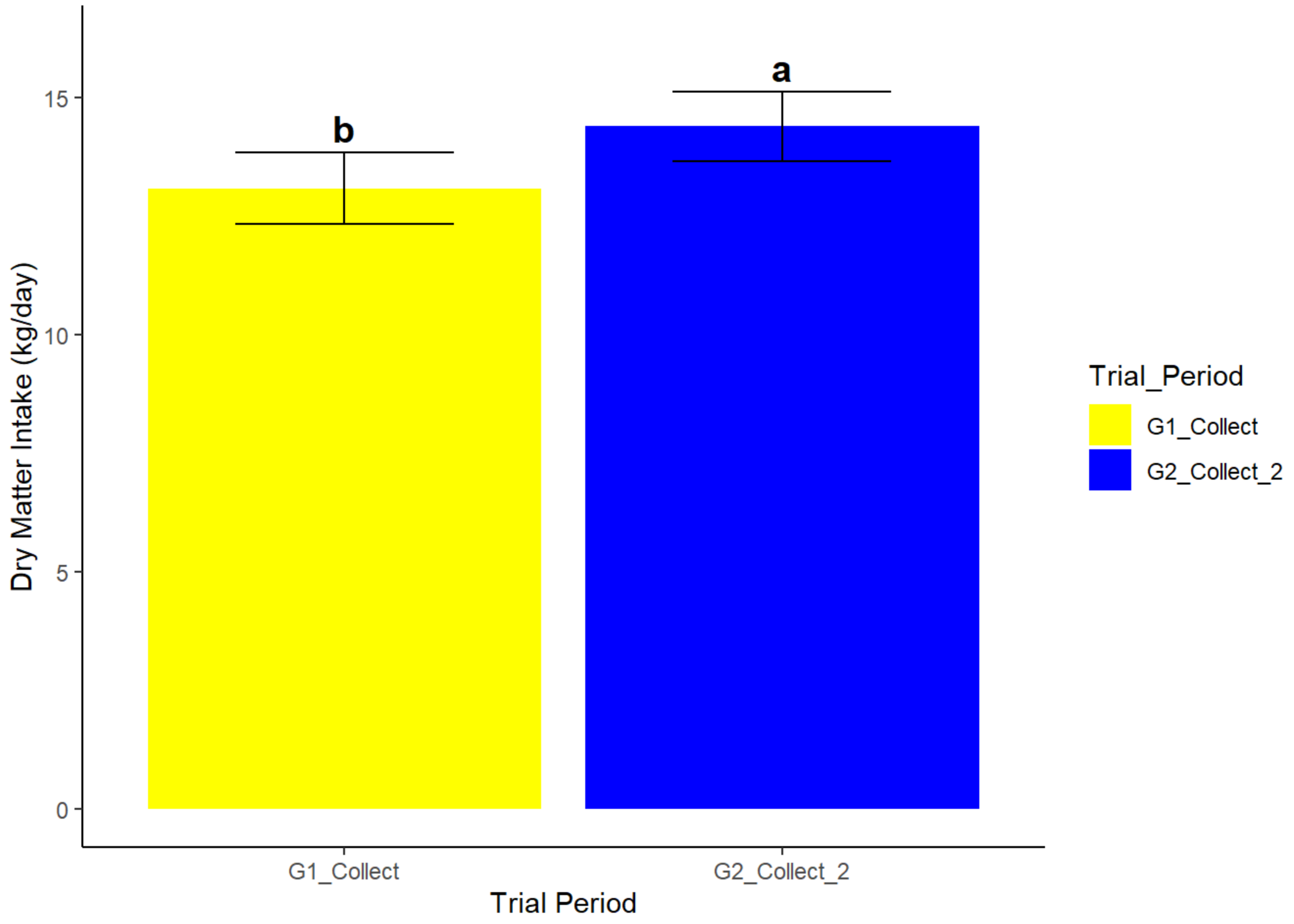

```
# Fit mixed model for CH4
response_var_CH4 <- "Daily_Avg_CH4"
y_label_CH4 <- expression(CH[4] ~ (g/day))
formula_CH4 <- as.formula(paste(response_var_CH4, "~ Trial_Period + (1 | AnimalTag)"))

# Model
model_CH4 <- lmer(formula_CH4, data = subset_data_clean)
summary(model_CH4)
```

```
## Linear mixed model fit by REML. t-tests use Satterthwaite's method [
## lmerModLmerTest]
## Formula: formula_CH4
##    Data: subset_data_clean
##
## REML criterion at convergence: 469.7
##
## Scaled residuals:
##      Min       1Q   Median       3Q      Max
## -2.90580 -0.38910  0.00624  0.38896  2.64348
##
## Random effects:
##  Groups    Name        Variance Std.Dev.
##  AnimalTag (Intercept)  733.4   27.08
##  Residual              3246.2   56.98
## Number of obs: 44, groups:  AnimalTag, 7
##
## Fixed effects:
##                          Estimate Std. Error     df t value Pr(>|t|)
## (Intercept)                220.95      17.94  15.73  12.318 1.72e-09 ***
## Trial_PeriodG2_Collect_2    54.41      18.92  42.00   2.876   0.0063 **
## ---
## Signif. codes:  0 '***' 0.001 '**' 0.01 '*' 0.05 '.' 0.1 ' ' 1
##
## Correlation of Fixed Effects:
##             (Intr)
## Trl_PG2_C_2 -0.636
```

```
# EMM
emm_CH4 <- emmeans(model_CH4, ~ Trial_Period)
emm_df_CH4 <- as.data.frame(emm_CH4)
emm_df_CH4$Label <- c("b", "a")

# Plot
ggplot(emm_df_CH4, aes(x = Trial_Period, y = emmean, fill = Trial_Period)) +
  geom_col() +
  geom_errorbar(aes(ymin = emmean - SE, ymax = emmean + SE), width = 0.5) +
  geom_text(aes(label = Label, y = emmean + SE + 10), fontface = "bold", size = 5) +
  scale_y_continuous(limits = c(0, 310), expand = c(0, 0)) +
  labs(x = "Trial Period", y = y_label_CH4) +
  scale_fill_manual(values = c("yellow", "blue")) +
  theme_classic()
```

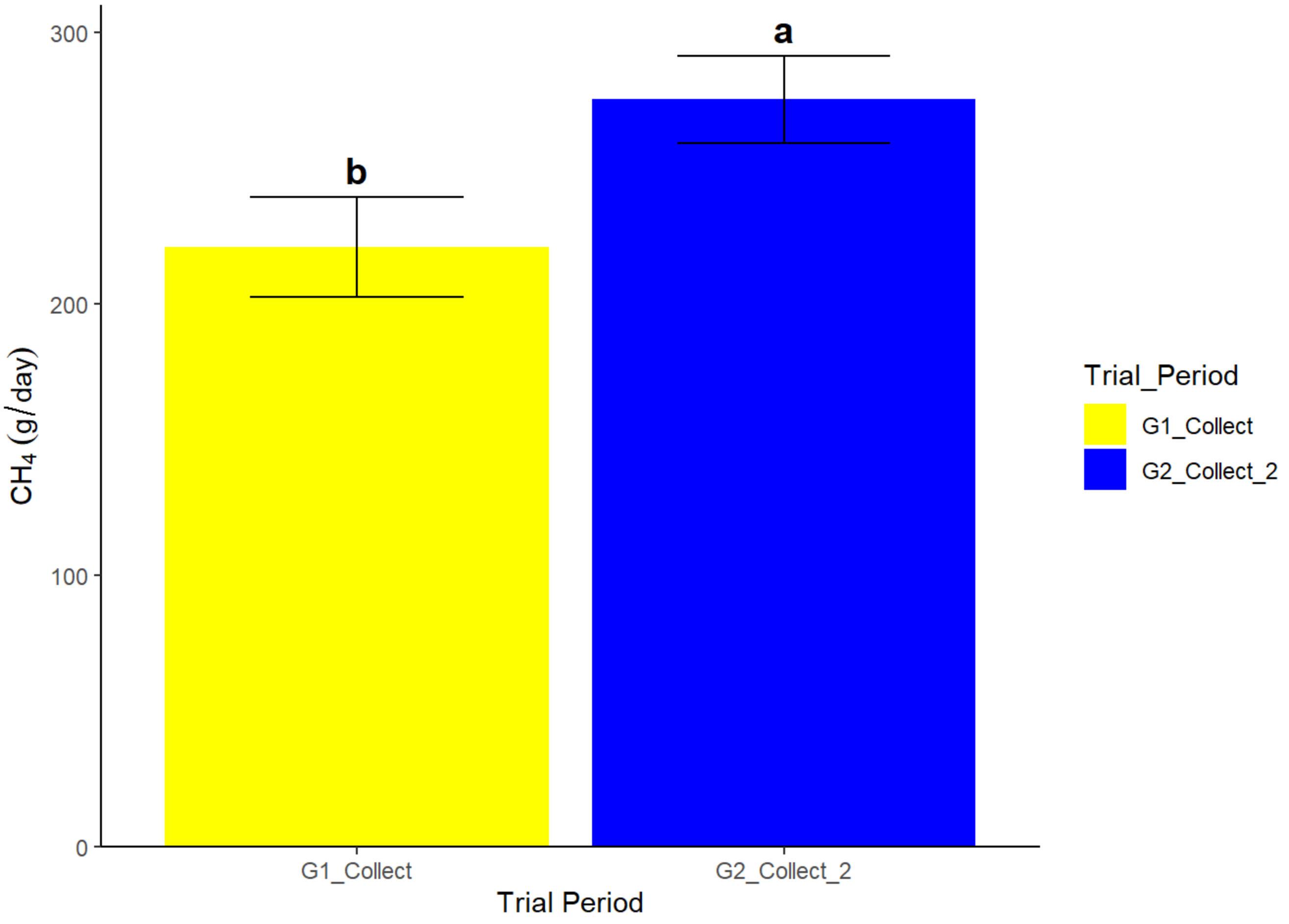


```
# CO2 setup
response_var_CO2 <- "Daily_Avg_CO2"
y_label_CO2 <- expression(CO[2] ~ (g/day))
formula_CO2 <- as.formula(paste(response_var_CO2, "~ Trial_Period + (1 | AnimalTag)"))

# Model
model_CO2 <- lmer(formula_CO2, data = subset_data_clean)
summary(model_CO2)
```

```
## Linear mixed model fit by REML. t-tests use Satterthwaite's method [
## lmerModLmerTest]
## Formula: formula_CO2
##    Data: subset_data_clean
##
## REML criterion at convergence: 731.8
##
## Scaled residuals:
##      Min       1Q   Median       3Q      Max
## -2.78019 -0.46998  0.03694  0.68831  1.77680
##
## Random effects:
##  Groups    Name        Variance Std.Dev.
##  AnimalTag (Intercept)  393914   627.6
##  Residual              1659118  1288.1
## Number of obs: 44, groups:  AnimalTag, 7
##
## Fixed effects:
##                          Estimate Std. Error      df t value Pr(>|t|)
## (Intercept)               6968.84     409.36   14.61  17.024 4.87e-11 ***
## Trial_PeriodG2_Collect_2  1196.79     428.52   42.00   2.793  0.00783 **
## ---
## Signif. codes:  0 '***' 0.001 '**' 0.01 '*' 0.05 '.' 0.1 ' ' 1
##
## Correlation of Fixed Effects:
##             (Intr)
## Trl_PG2_C_2 -0.631
```

```
# Get Estimate Marginal Means
emm_CO2 <- emmeans(model_CO2, ~ Trial_Period)
emm_df_CO2 <- as.data.frame(emm_CO2)
emm_df_CO2$Label <- c("b", "a")
emm_df_CO2$Label_y <- emm_df_CO2$emmean + emm_df_CO2$SE + 0.05 * max(emm_df_CO2$emmean)

# Plot
ggplot(emm_df_CO2, aes(x = Trial_Period, y = emmean, fill = Trial_Period)) +
  geom_col() +
  geom_errorbar(aes(ymin = emmean - SE, ymax = emmean + SE), width = 0.5) +
  geom_text(aes(label = Label, y = Label_y), fontface = "bold", size = 5) +
  scale_y_continuous(limits = c(0, 10000), expand = c(0, 0)) +
  labs(x = "Trial Period", y = y_label_CO2) +
  scale_fill_manual(values = c("yellow", "blue")) +
  theme_classic()
```

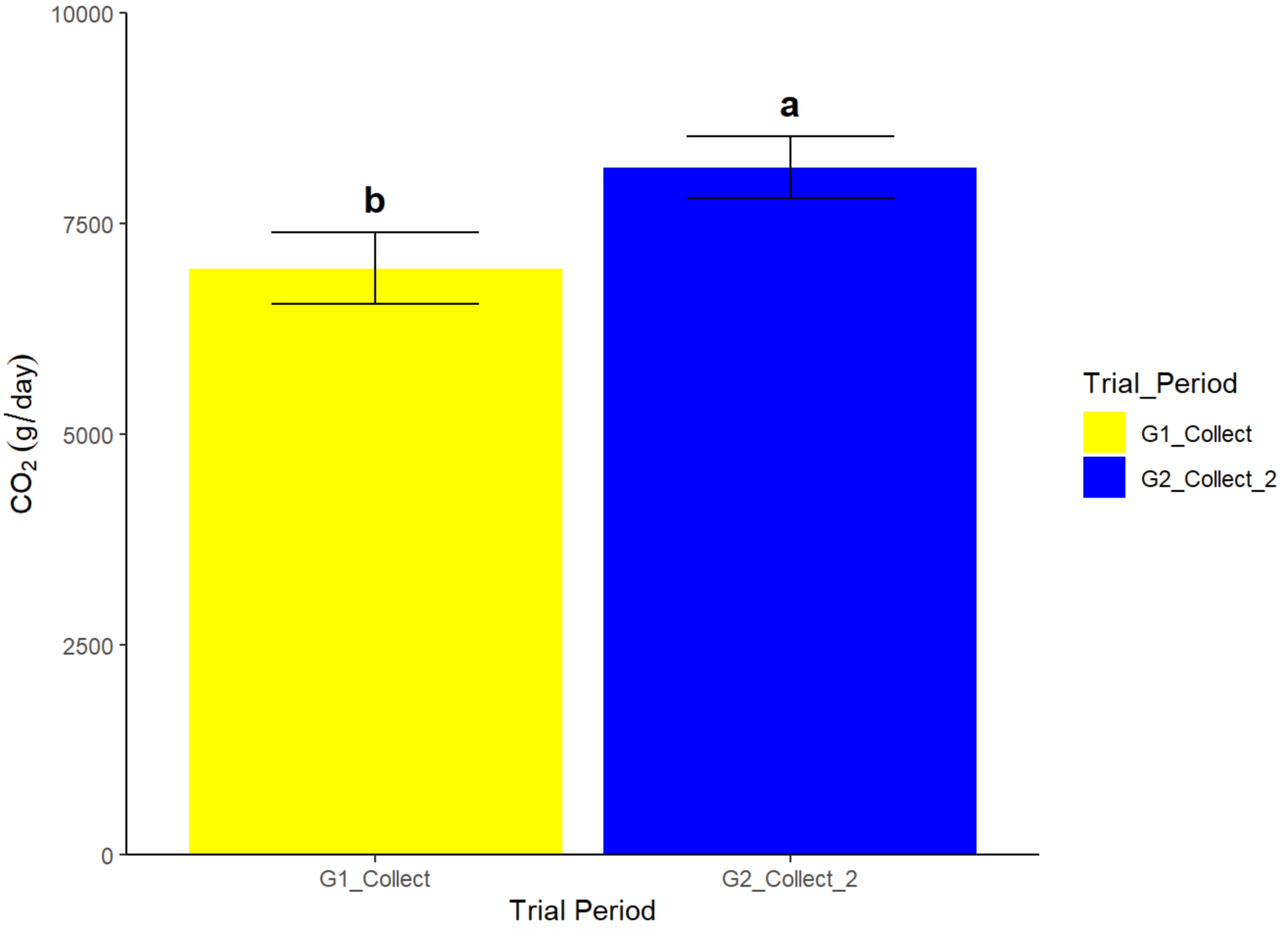


```
# O2 setup
response_var_O2 <- "Daily_Avg_O2"
y_label_O2 <- expression(O[2] ~ (g/day))
formula_O2 <- as.formula(paste(response_var_O2, "~ Trial_Period + (1 | AnimalTag)"))

# Model
model_O2 <- lmer(formula_O2, data = subset_data_clean)
summary(model_O2)
```

```
## Linear mixed model fit by REML. t-tests use Satterthwaite's method [
## lmerModLmerTest]
## Formula: formula_O2
##    Data: subset_data_clean
##
## REML criterion at convergence: 709.6
##
## Scaled residuals:
##     Min      1Q  Median      3Q     Max
## -3.3280 -0.3718  0.0472  0.5543  2.0155
##
## Random effects:
##  Groups    Name        Variance Std.Dev.
##  AnimalTag (Intercept)  141476   376.1
##  Residual              1014618  1007.3
## Number of obs: 44, groups:  AnimalTag, 7
##
## Fixed effects:
##                         Estimate Std. Error      df t value Pr(>|t|)
## (Intercept)              5253.14     292.67   14.90   17.95 1.69e-11 ***
## Trial_PeriodG2_Collect_2   574.54     328.39   41.75    1.75   0.0875 .
## ---
## Signif. codes:  0 '***' 0.001 '**' 0.01 '*' 0.05 '.' 0.1 ' ' 1
##
## Correlation of Fixed Effects:
##             (Intr)
## Trl_PG2_C_2 -0.676
```

```
# EMM
emm_O2 <- emmeans(model_O2, ~ Trial_Period)
emm_df_O2 <- as.data.frame(emm_O2)
emm_df_O2$Label <- c("a", "a")
emm_df_O2$Label_y <- emm_df_O2$emmean + emm_df_O2$SE + 0.05 * max(emm_df_O2$emmean, na.rm = TRU
E)

# Plot
ggplot(emm_df_O2, aes(x = Trial_Period, y = emmean, fill = Trial_Period)) +
  geom_col() +
  geom_errorbar(aes(ymin = emmean - SE, ymax = emmean + SE), width = 0.5) +
  geom_text(aes(label = Label, y = Label_y), fontface = "bold", size = 5) +
  scale_y_continuous(
    limits = c(0, max(emm_df_O2$Label_y, na.rm = TRUE) + 0.05 * max(emm_df_O2$emmean, na.rm = TR
UE)),
    expand = c(0, 0)
  ) +
  labs(x = "Trial Period", y = y_label_O2) +
  scale_fill_manual(values = c("yellow", "blue")) +
  theme_classic()
```

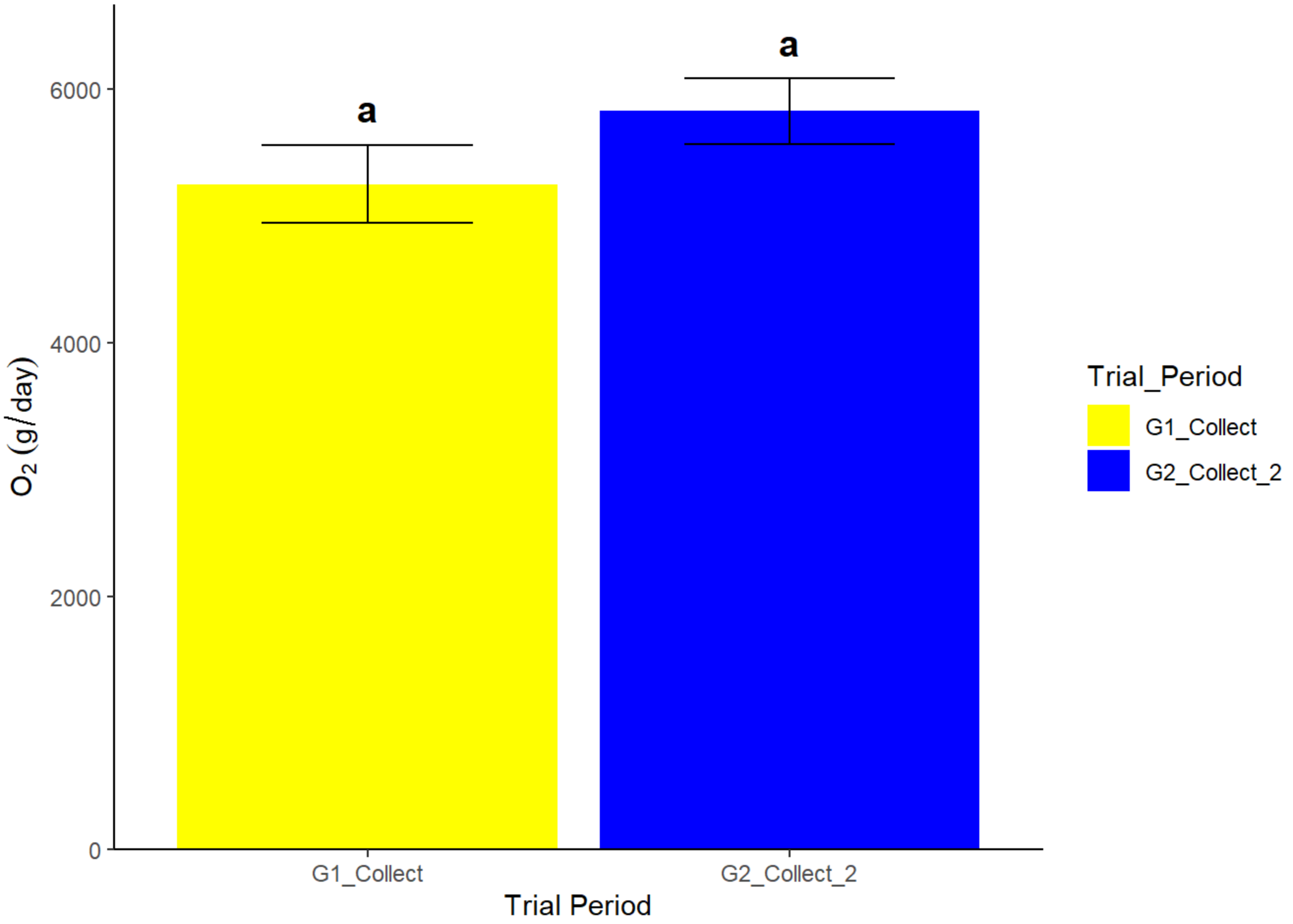

15. Test the Normality of Residuals.

```
# Example: for DMI model
# 1. Residual histogram
hist(residuals(model_DMI), main = "Histogram of Residuals", xlab = "Residuals")
```

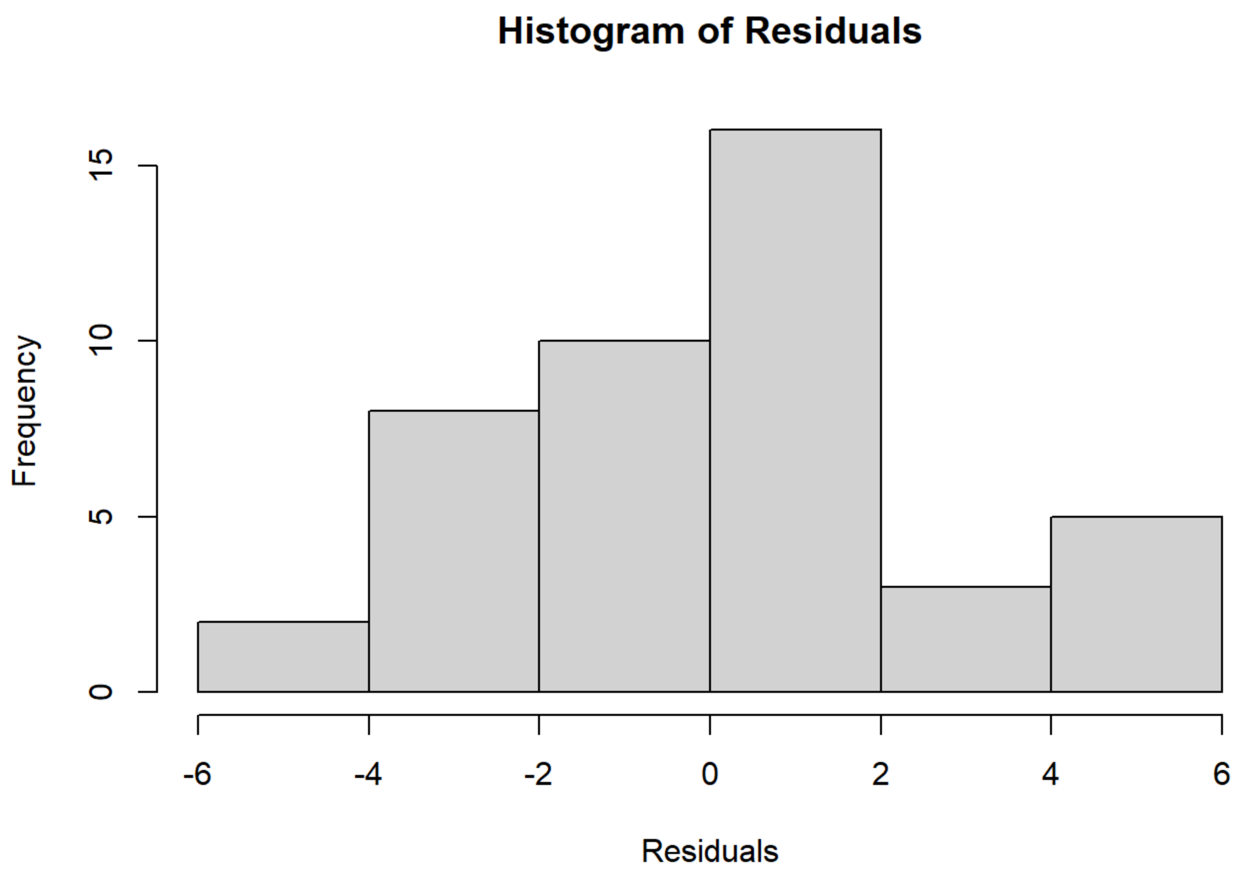


```
# 2. Q-Q Plot for normality
qqnorm(residuals(model_DMI))
qqline(residuals(model_DMI), col = "red", lwd = 2)
```

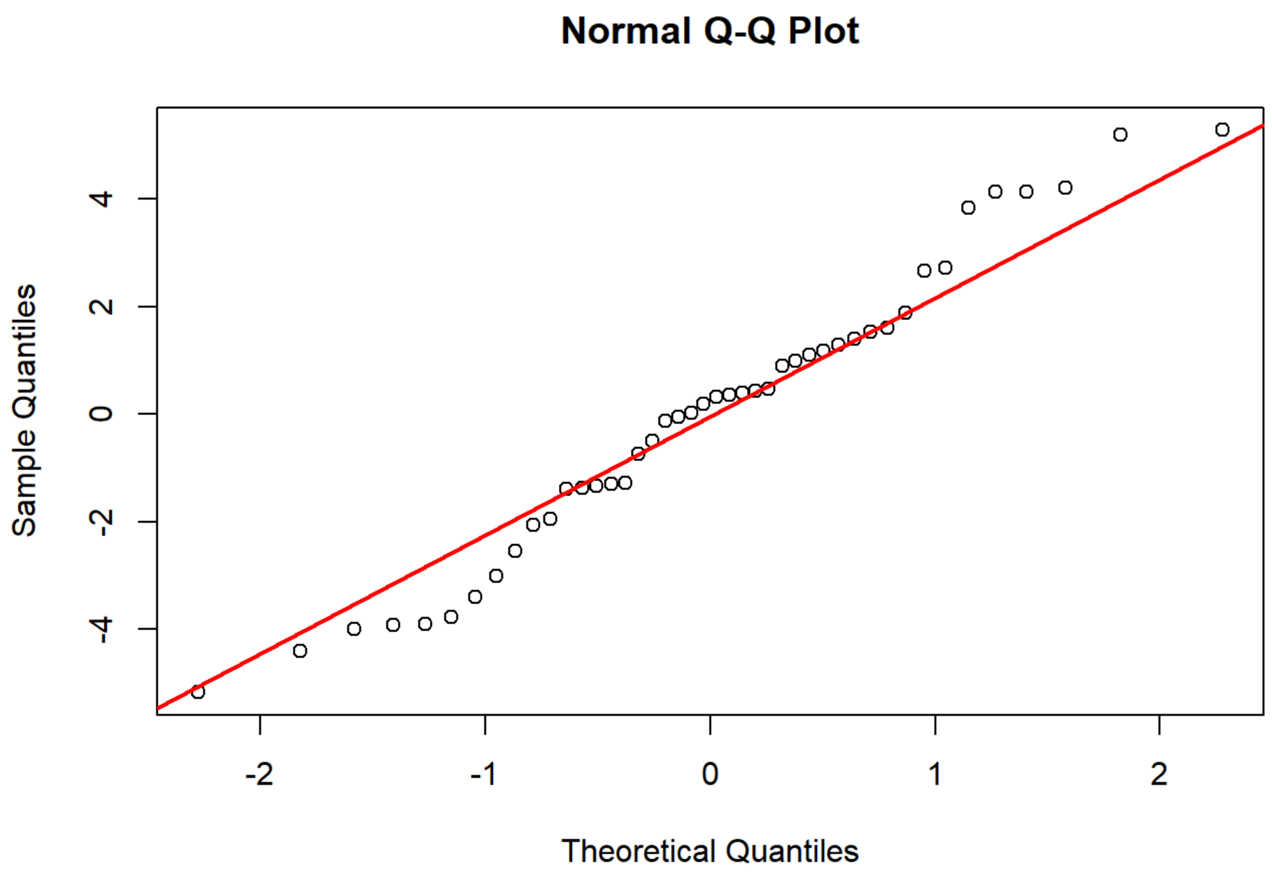


```
# 3. Shapiro-Wilk test (for small samples, < 5000)
shapiro.test(residuals(model_DMI))  # returns W and p-value
```

```
##
##  Shapiro-Wilk normality test
##
## data:  residuals(model_DMI)
## W = 0.97476, p-value = 0.44
```

```
# 4. Plot residuals vs. fitted values (homoscedasticity check)
plot(fitted(model_DMI), residuals(model_DMI),
     main = "Residuals vs Fitted",
     xlab = "Fitted Values", ylab = "Residuals")
abline(h = 0, col = "blue", lwd = 2)
```

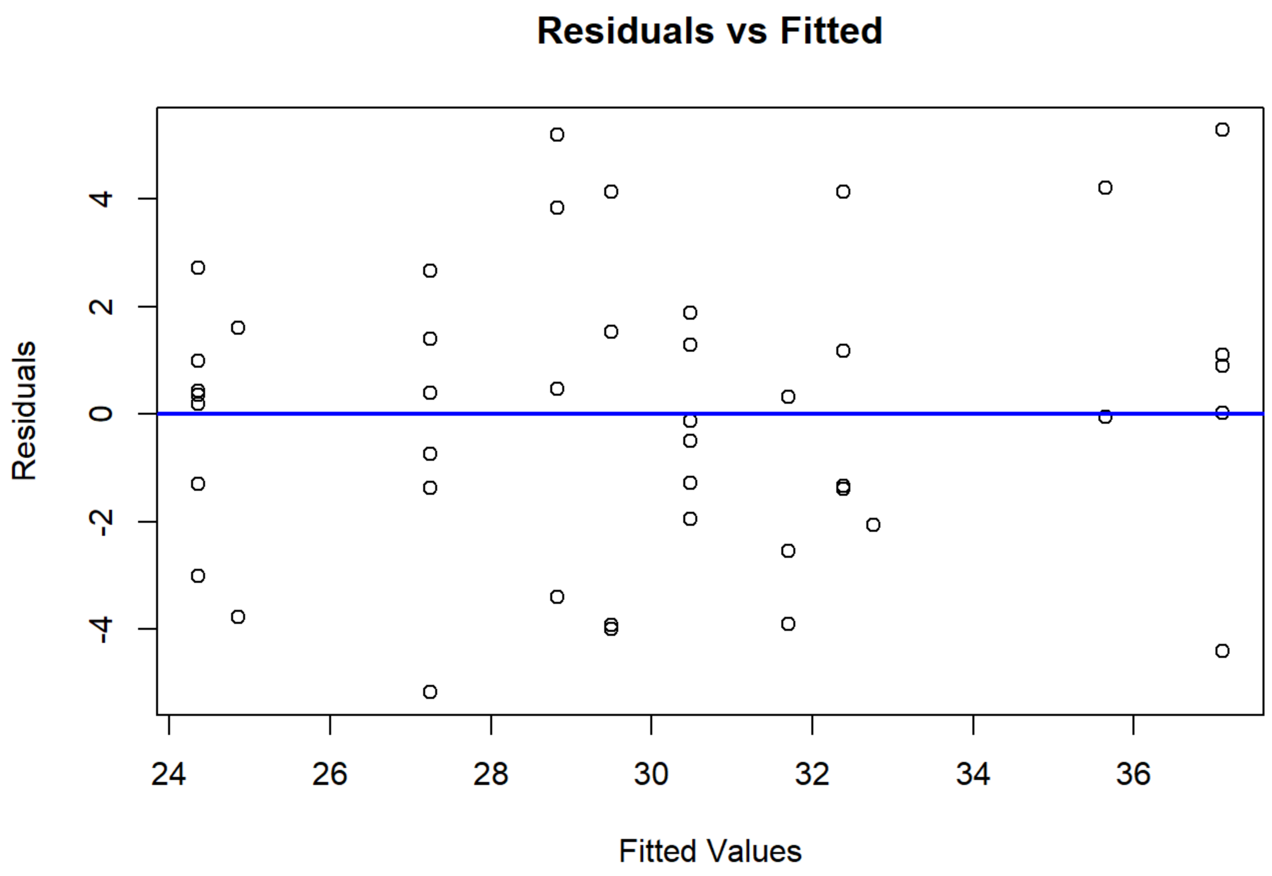


```
# Example: for CH4 model
# 1. Residual histogram
hist(residuals(model_CH4), main = "Histogram of Residuals", xlab = "Residuals")
```

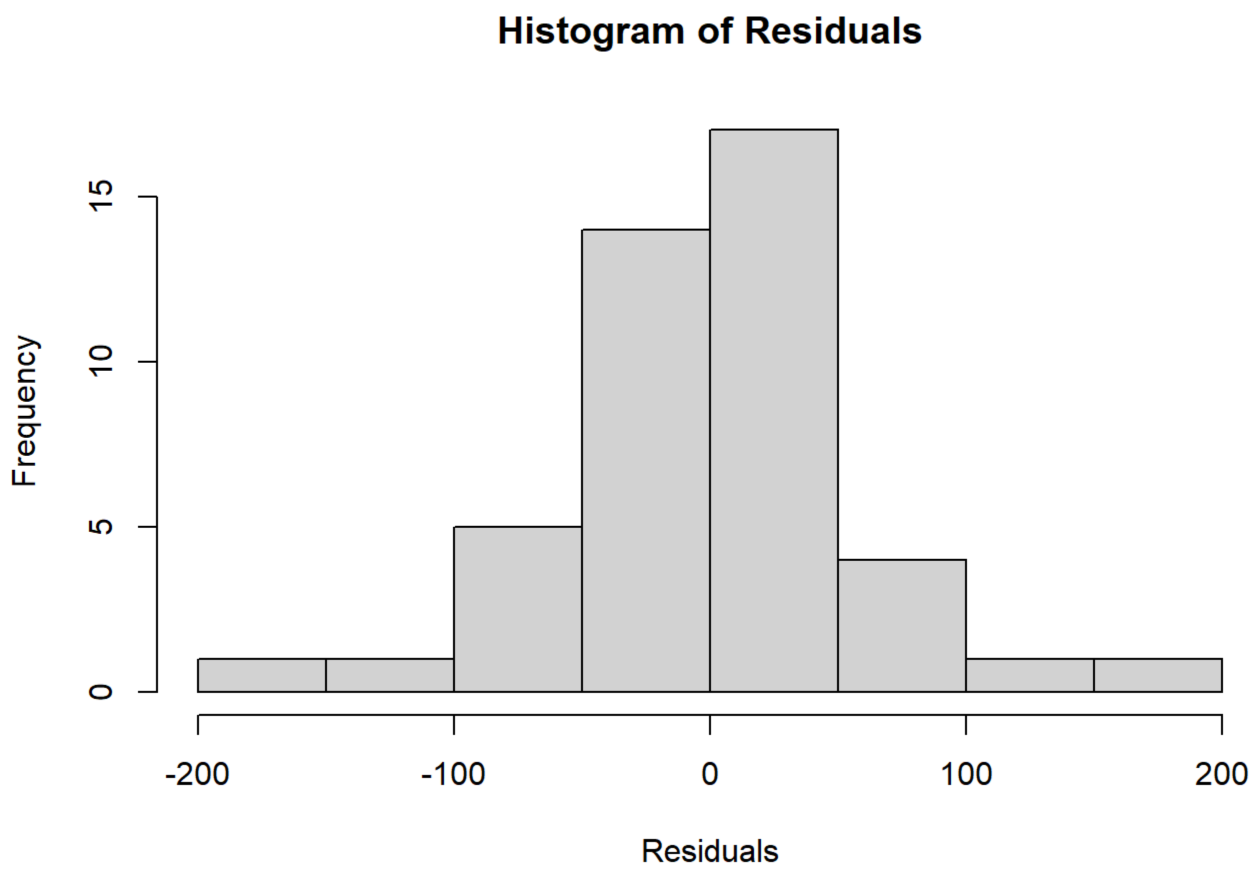


```
# 2. Q-Q Plot for normality
qqnorm(residuals(model_CH4))
qqline(residuals(model_CH4), col = "red", lwd = 2)
```

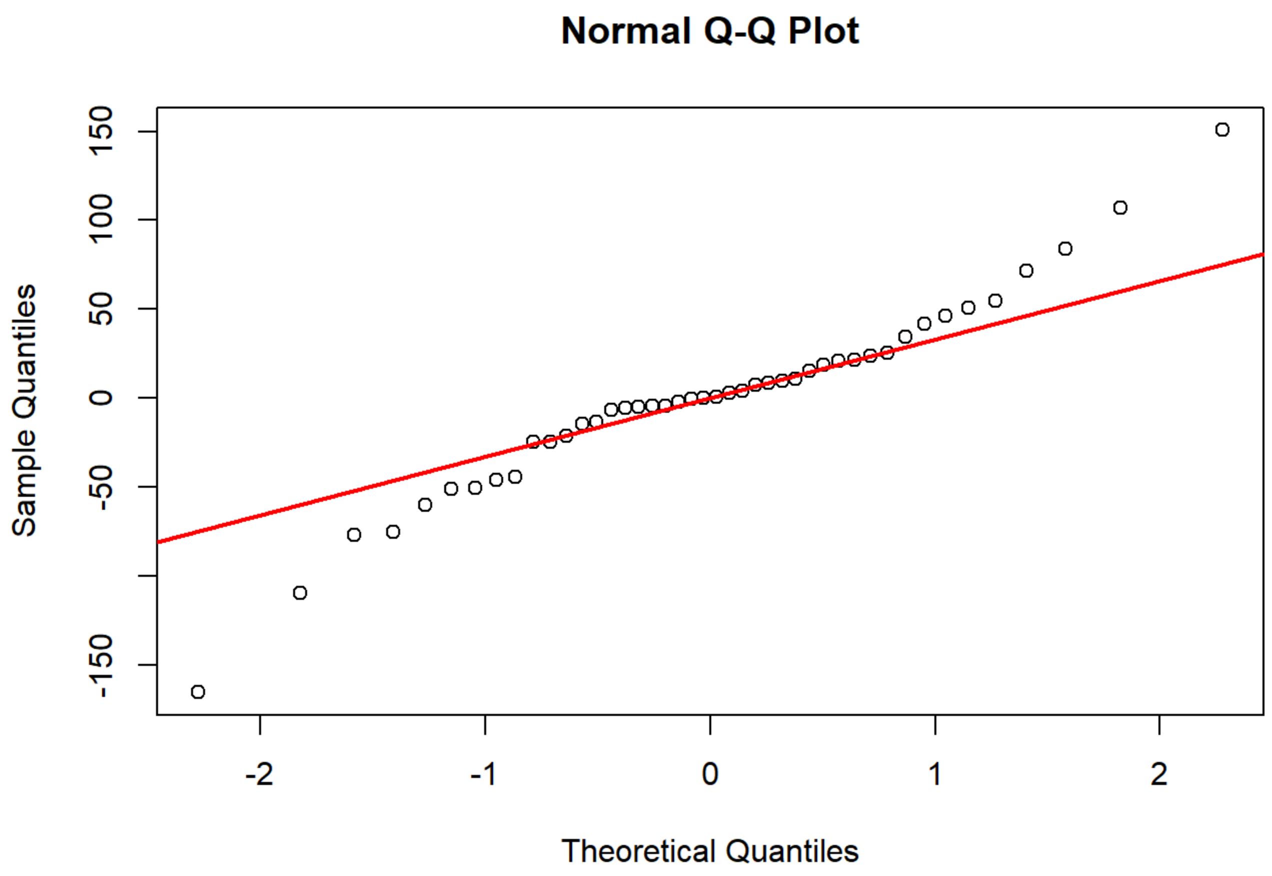


```
# 3. Shapiro-Wilk test (for small samples, < 5000)
shapiro.test(residuals(model_CH4))  # returns W and p-value
```

```
##
##  Shapiro-Wilk normality test
##
## data:  residuals(model_CH4)
## W = 0.95482, p-value = 0.08326
```

```
# 4. Plot residuals vs. fitted values (homoscedasticity check)
plot(fitted(model_CH4), residuals(model_CH4),
     main = "Residuals vs Fitted",
     xlab = "Fitted Values", ylab = "Residuals")
abline(h = 0, col = "blue", lwd = 2)
```

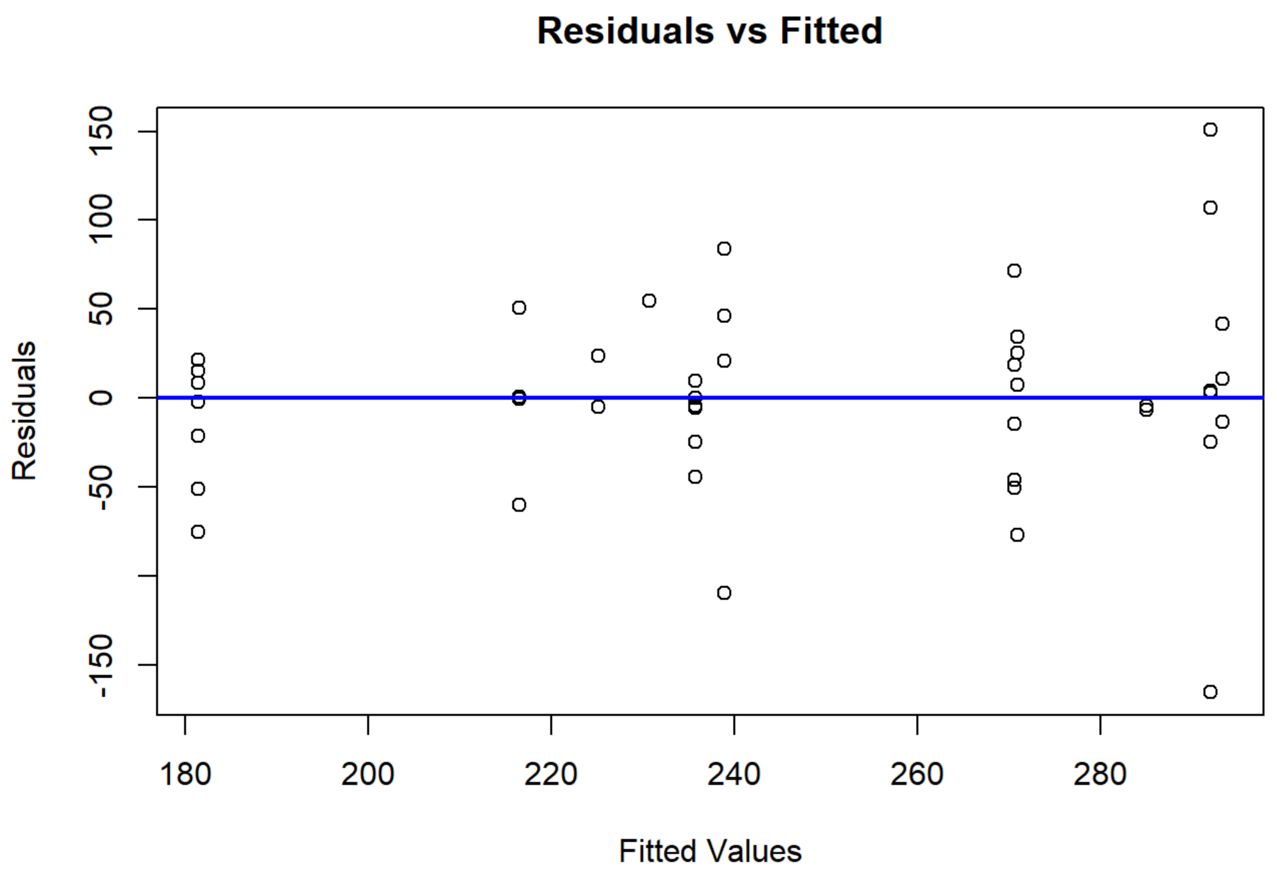


```
# Example: for CO2 model
# 1. Residual histogram
hist(residuals(model_CO2), main = "Histogram of Residuals", xlab = "Residuals")
```

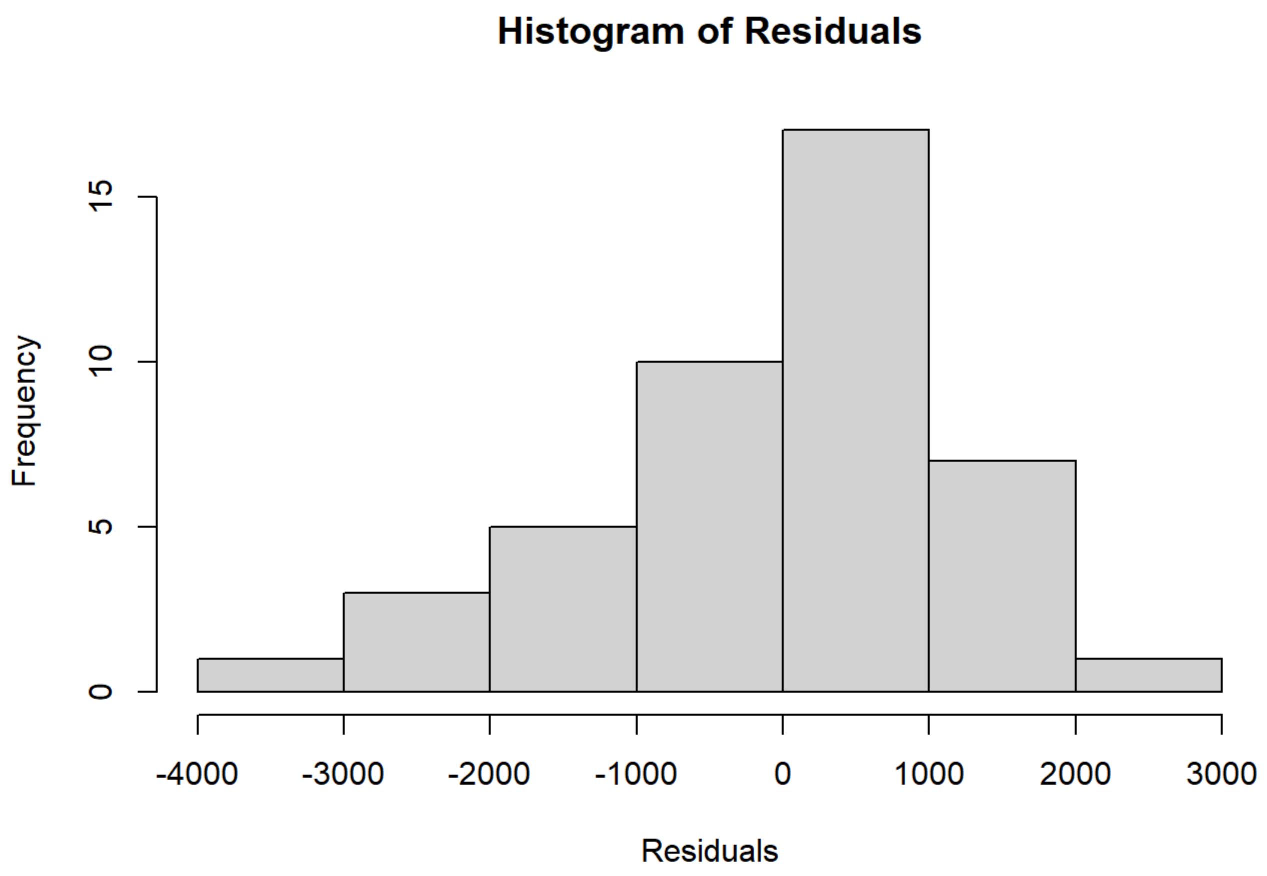


```
# 2. Q-Q Plot for normality
qqnorm(residuals(model_CO2))
qqline(residuals(model_CO2), col = "red", lwd = 2)
```

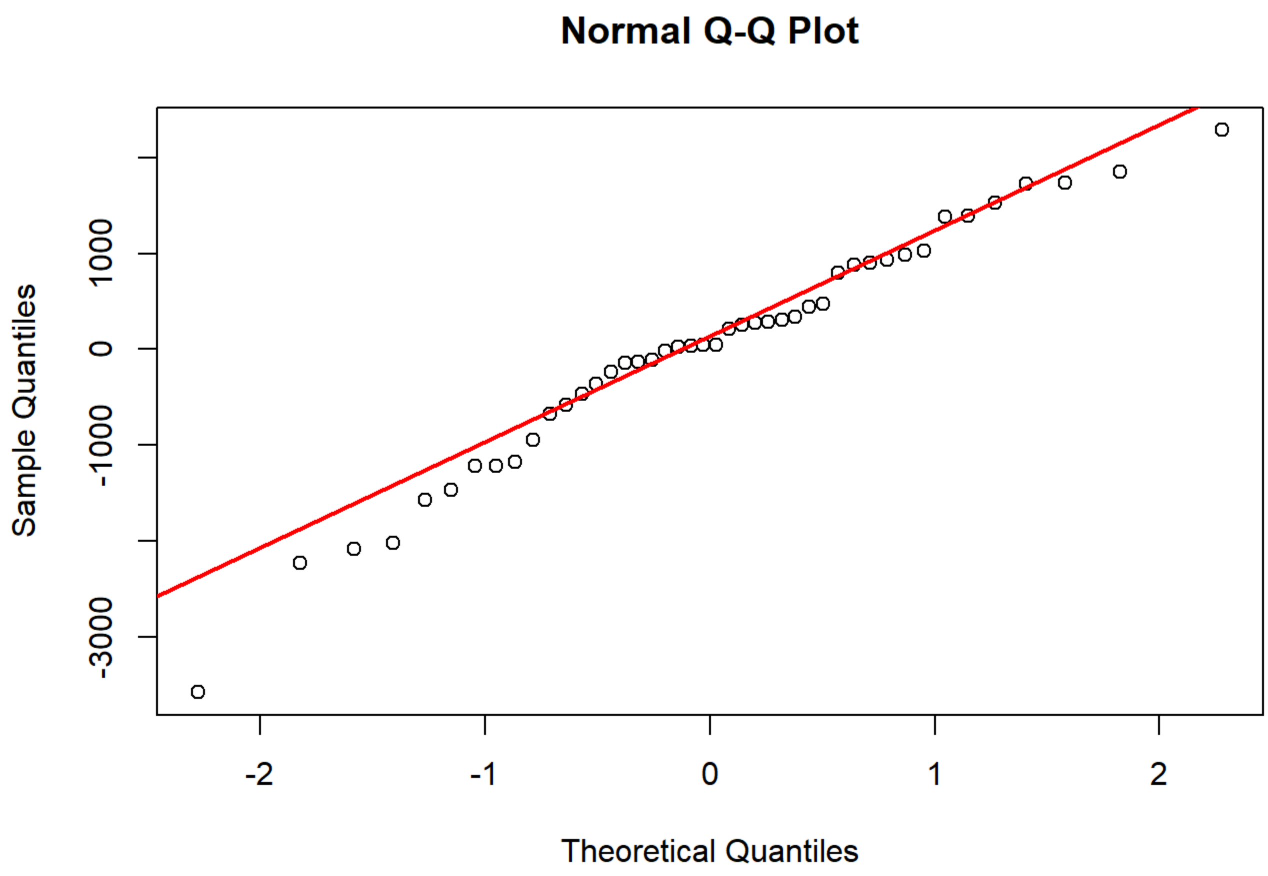


```
# 3. Shapiro-Wilk test (for small samples, < 5000)
shapiro.test(residuals(model_CO2))  # returns W and p-value
```

```
##
##  Shapiro-Wilk normality test
##
## data:  residuals(model_CO2)
## W = 0.97089, p-value = 0.3253
```

```
# 4. Plot residuals vs. fitted values (homoscedasticity check)
plot(fitted(model_CO2), residuals(model_CO2),
     main = "Residuals vs Fitted",
     xlab = "Fitted Values", ylab = "Residuals")
abline(h = 0, col = "blue", lwd = 2)
```

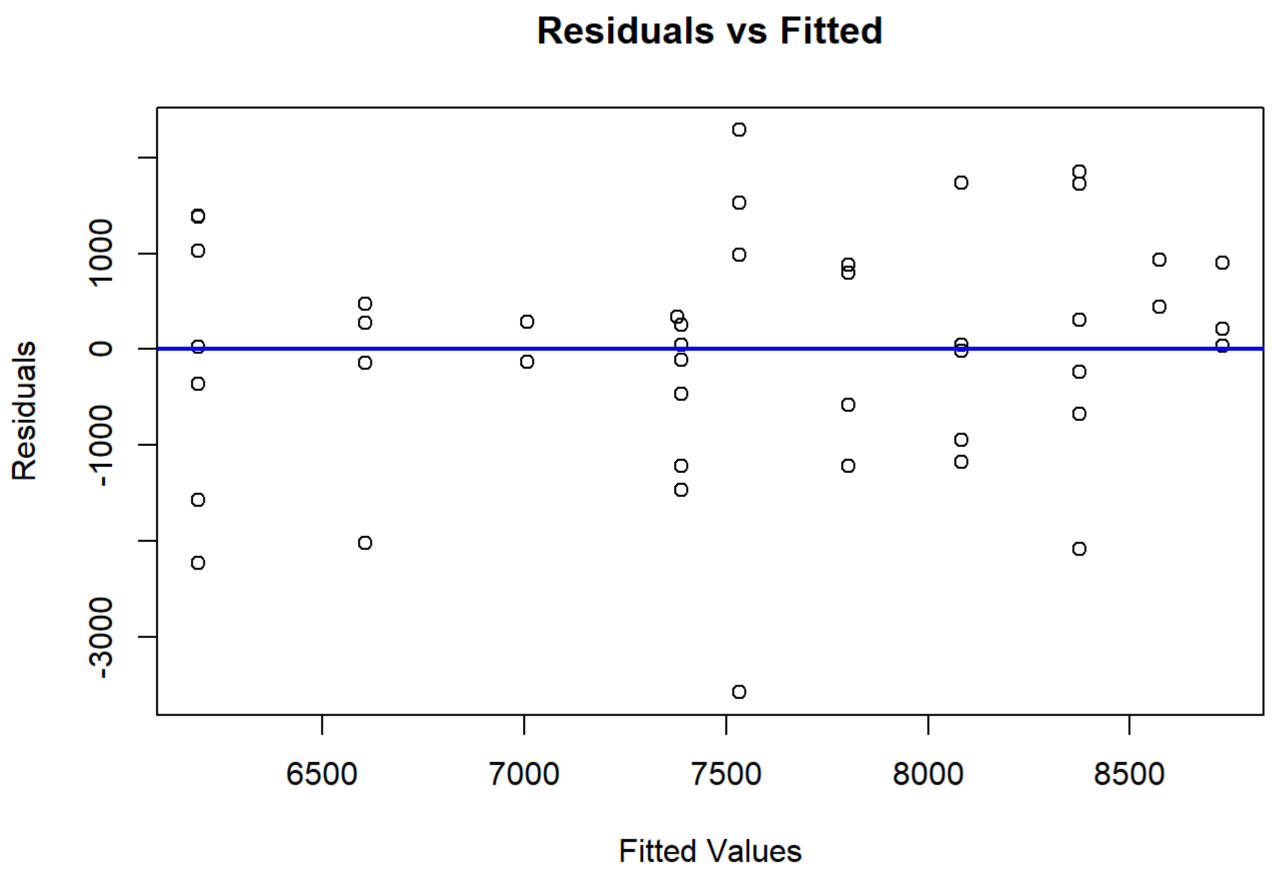


```
# Example: for O2 model
# 1. Residual histogram
hist(residuals(model_O2), main = "Histogram of Residuals", xlab = "Residuals")
```

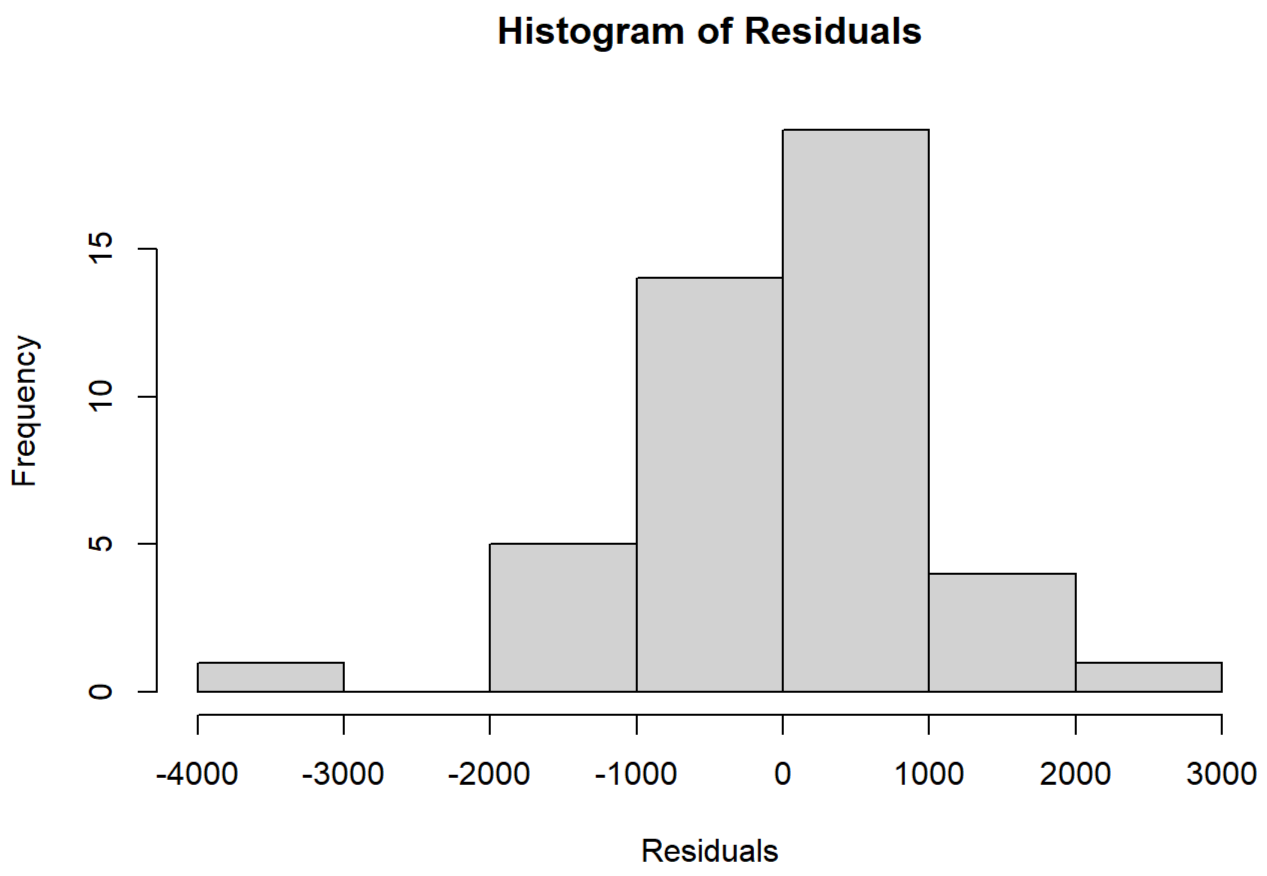


```
# 2. Q-Q Plot for normality
qqnorm(residuals(model_O2))
qqline(residuals(model_O2), col = "red", lwd = 2)
```

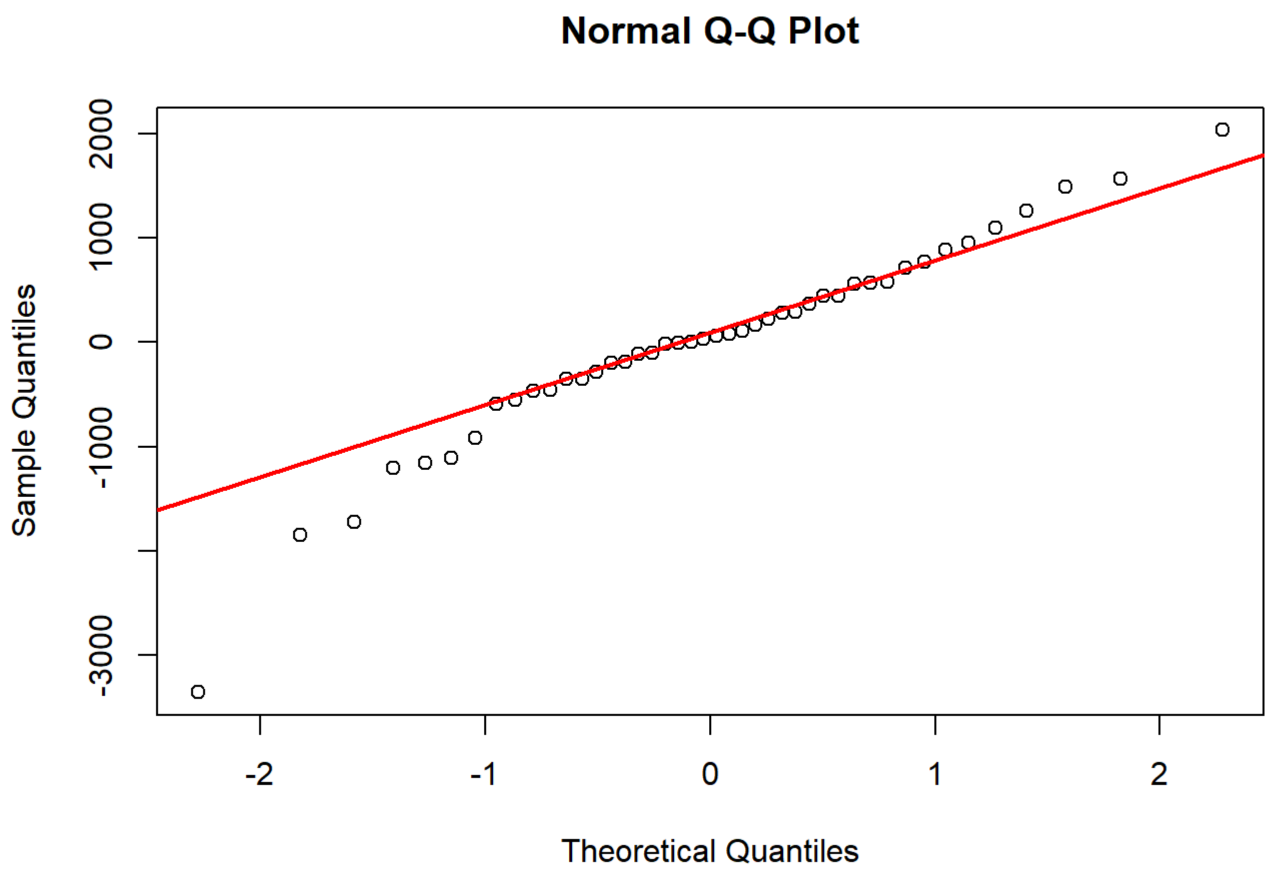


```
# 3. Shapiro-Wilk test (for small samples, < 5000)
shapiro.test(residuals(model_O2))  # returns W and p-value
```

```
##
##  Shapiro-Wilk normality test
##
## data:  residuals(model_O2)
## W = 0.9492, p-value = 0.05138
```

```
# 4. Plot residuals vs. fitted values (homoscedasticity check)
plot(fitted(model_O2), residuals(model_O2),
     main = "Residuals vs Fitted",
     xlab = "Fitted Values", ylab = "Residuals")
abline(h = 0, col = "blue", lwd = 2)
```

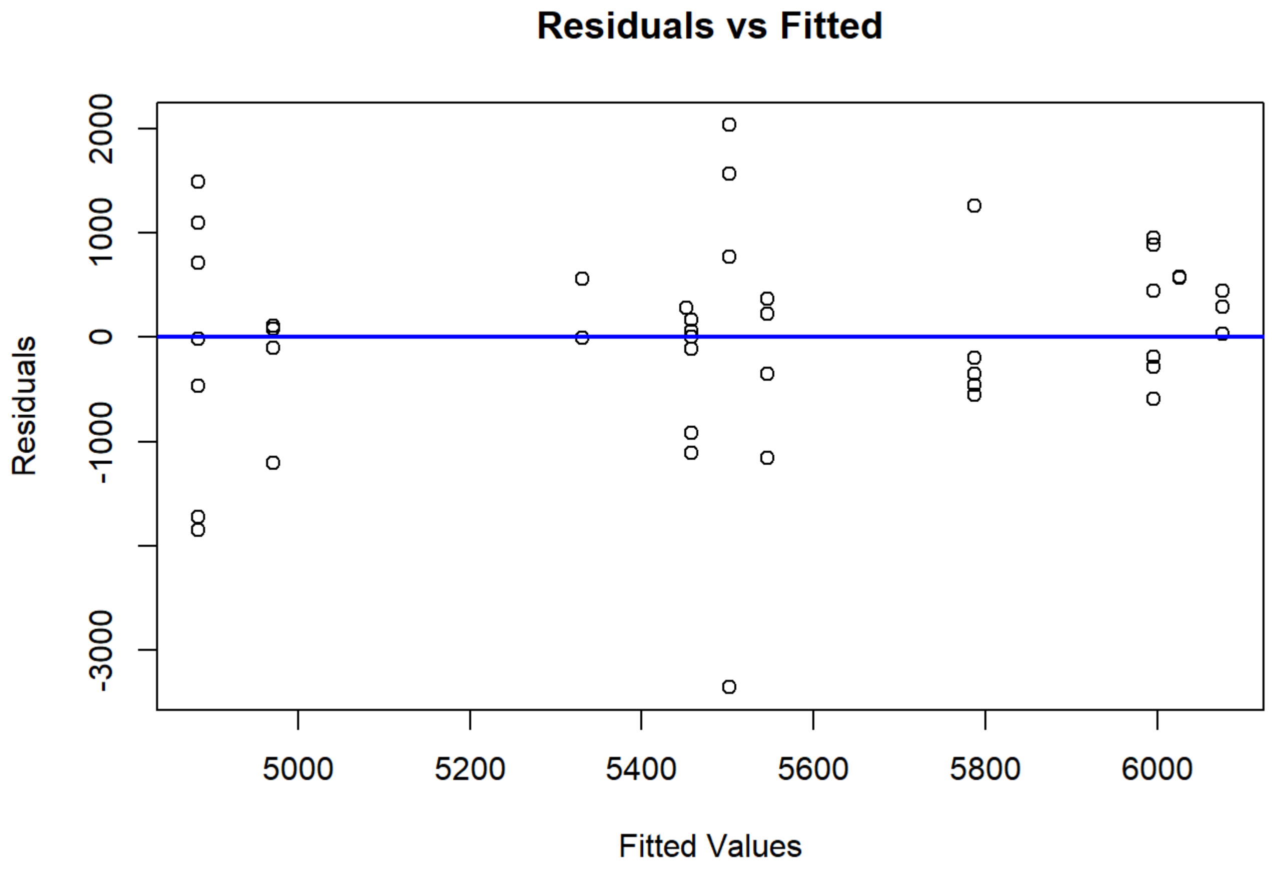

16. Estimate DMI Using Standard Nutrition Equations

Here the NASEM (2016) intake equation and percent body weight equations are deployed using the weight and nutrition data collected from the study. The DMI estimates are then plotted against observed values and the adjusted R2 and mean bias are reported on plots. Here the NASEM (2016) intake equation and percent body weight equations are deployed using the weight and nutrition data collected from the study. The DMI estimates are then plotted against observed values and the adjusted R2 and mean bias are reported on plots.

```
#library(dplyr)
#library(ggplot2)

# -------------------------------------------------------------------------------
# 1) Prepare data: compute NASEM and 1.8% BW predictions
# -------------------------------------------------------------------------------
tdn_values <- c(G1_Collect = 55.4, G2_Collect_2 = 47.6)

DMI_model_data <- subset_data_clean %>%
  # make sure Weight_kg already exists (you converted earlier)
  mutate(
    BW_75      = Weight_kg^0.75,
    TDN        = case_when(
                   Trial_Period == "G1_Collect"   ~ tdn_values["G1_Collect"],
                   Trial_Period == "G2_Collect_2" ~ tdn_values["G2_Collect_2"]
                 ),
    DE         = (TDN / 100) * 4.409,
    ME         = 0.9611 * DE - 0.2999,
    NEm        = 1.37 * ME - (0.138^2) * 0.0105 * (ME^3) - 1.12,
    NEm_Intake = BW_75 * (0.04997 * NEm^2 + 0.04631),
    NASEM_Pred = NEm_Intake / NEm,        # kg/day
    BW_1.8_Pred = Weight_kg * 0.018       # kg/day
  )

# -------------------------------------------------------------------------------
# 2) Metric function: adjusted R² + mean bias
# -------------------------------------------------------------------------------
model_metrics <- function(obs, pred) {
  fit       <- lm(obs ~ pred)
  adj_r2    <- summary(fit)$adj.r.squared
  mean_bias <- mean(pred - obs, na.rm = TRUE)
  list(adj_r2    = round(adj_r2,    3),
       mean_bias = round(mean_bias, 3))
}

# compute overall metrics
metrics_nasem <- model_metrics(DMI_model_data$DMI_kg, DMI_model_data$NASEM_Pred)
metrics_bw    <- model_metrics(DMI_model_data$DMI_kg, DMI_model_data$BW_1.8_Pred)

# -------------------------------------------------------------------------------
# 3) Plotting function: observed vs. predicted with parse-safe annotation
# -------------------------------------------------------------------------------
plot_obs_vs_pred <- function(data, pred_col, model_name, metrics) {
  # determine label location
  xpos <- max(data[[pred_col]], na.rm = TRUE)
  ypos <- min(data[["DMI_kg"]],     na.rm = TRUE)

  # build a plotmath-safe label
  label_text <- paste0(
    "Adj~R^2==",    metrics$adj_r2,
    "~','~Mean~Bias==", metrics$mean_bias, "~kg/day"
  )
```

```
  ggplot(data, aes(x = .data[[pred_col]], y = .data[["DMI_kg"]], color = AnimalTag)) +
    geom_point(size = 3, alpha = 0.7) +
    geom_smooth(method = "lm", se = FALSE, color = "black", linetype = "dashed") +
    annotate(
      "text",
      x     = xpos,
      y     = ypos,
      label = label_text,
      parse = TRUE,
      hjust = 1,
      vjust = 0,
      size  = 4
    ) +
    labs(
      title = paste("Observed vs Predicted DMI:", model_name),
      x     = "Predicted DMI (kg/day)",
      y     = "Observed DMI (kg/day)",
      color = "AnimalTag"
    ) +
    theme_minimal() +
    theme(legend.position = "bottom")
}

# --------------------------------------------------------------------------------
# 4) Helper to plot by Trial_Period
# --------------------------------------------------------------------------------
plot_by_group <- function(group_name, data, pred_col, model_name) {
  df      <- filter(data, Trial_Period == group_name)
  metrics <- model_metrics(df$DMI_kg, df[[pred_col]])
  plot_obs_vs_pred(df, pred_col, paste(model_name, "-", group_name), metrics)
}

# --------------------------------------------------------------------------------
# 5) Generate & display
# --------------------------------------------------------------------------------
plot_nasem  <- plot_obs_vs_pred(DMI_model_data, "NASEM_Pred",  "NASEM Model",     metrics_nasem)
plot_bw     <- plot_obs_vs_pred(DMI_model_data, "BW_1.8_Pred",  "1.8% BW Rule",    metrics_bw)
nasem_g1    <- plot_by_group("G1_Collect",    DMI_model_data, "NASEM_Pred",  "NASEM")
nasem_g2    <- plot_by_group("G2_Collect_2",  DMI_model_data, "NASEM_Pred",  "NASEM")
bw_g1       <- plot_by_group("G1_Collect",    DMI_model_data, "BW_1.8_Pred", "1.8% BW")
bw_g2       <- plot_by_group("G2_Collect_2",  DMI_model_data, "BW_1.8_Pred", "1.8% BW")

print(plot_nasem)
```

```
## `geom_smooth()` using formula = 'y ~ x'
```

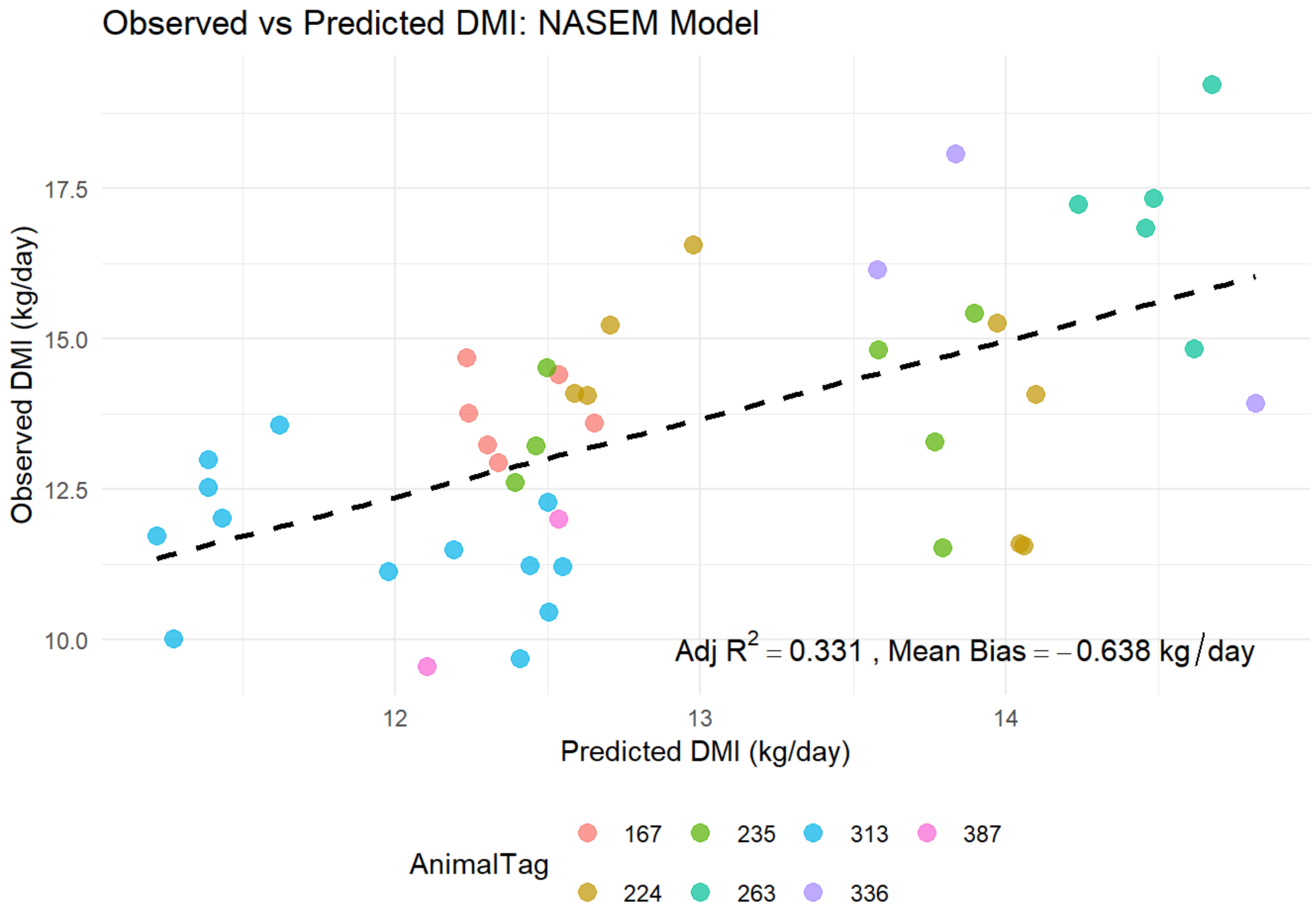


```
print(plot_bw)
```

```
## `geom_smooth()` using formula = 'y ~ x'
```

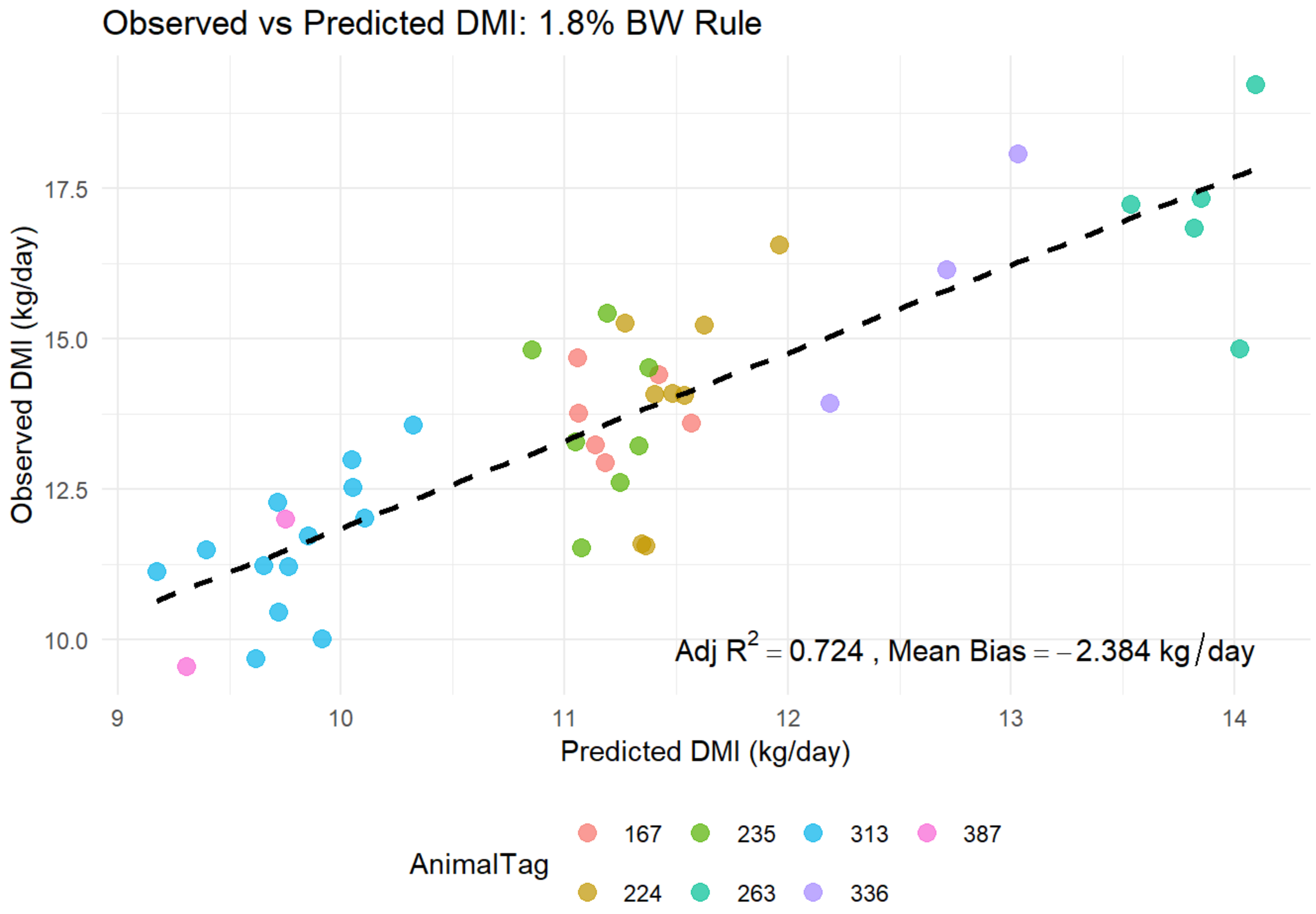


```
print(nasem_g1)
```

```
## `geom_smooth()` using formula = 'y ~ x'
```

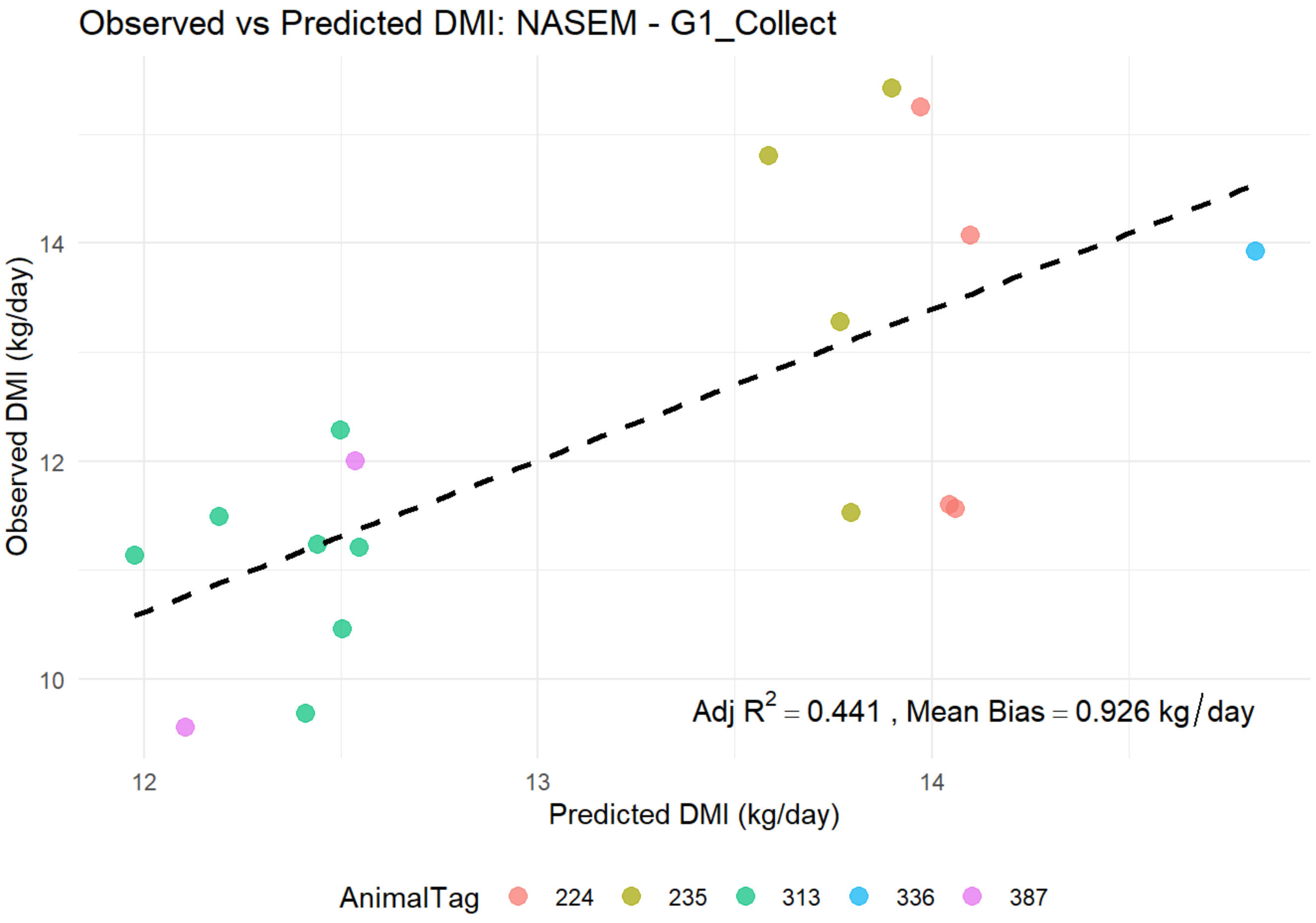


```
print(nasem_g2)
```

```
## `geom_smooth()` using formula = 'y ~ x'
```

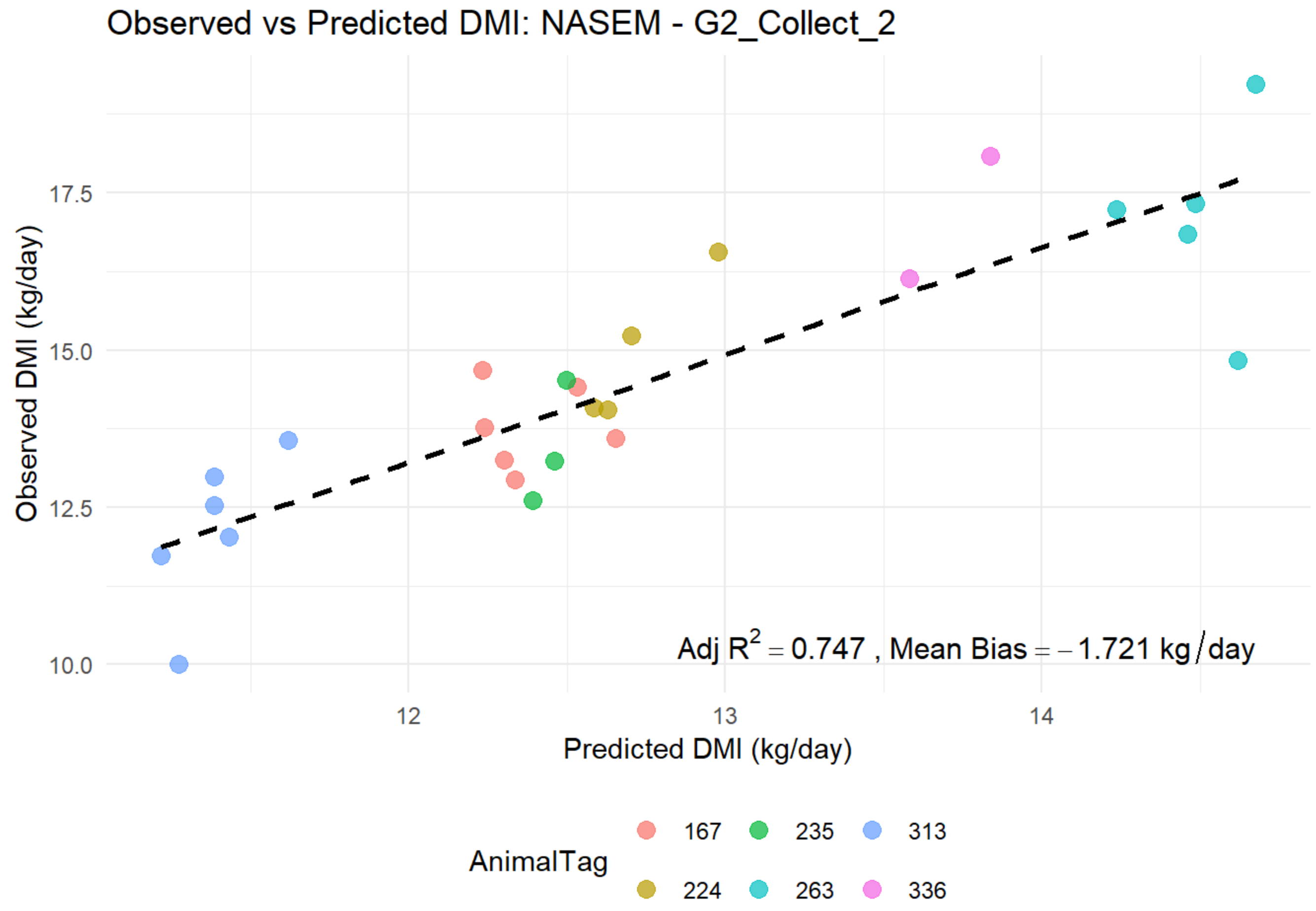


```
print(bw_g1)
```

```
## `geom_smooth()` using formula = 'y ~ x'
```

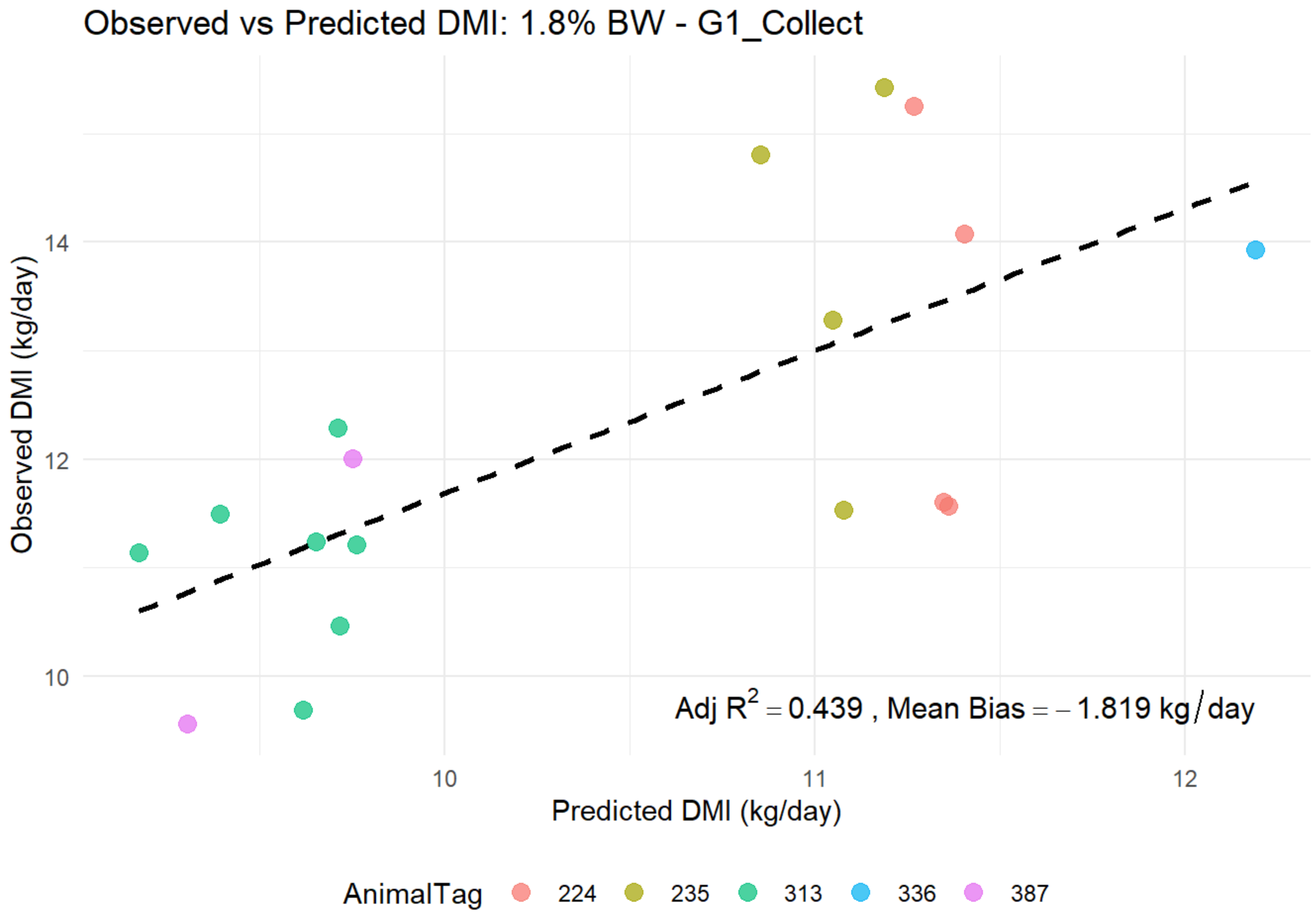


```
print(bw_g2)
```

```
## `geom_smooth()` using formula = 'y ~ x'
```

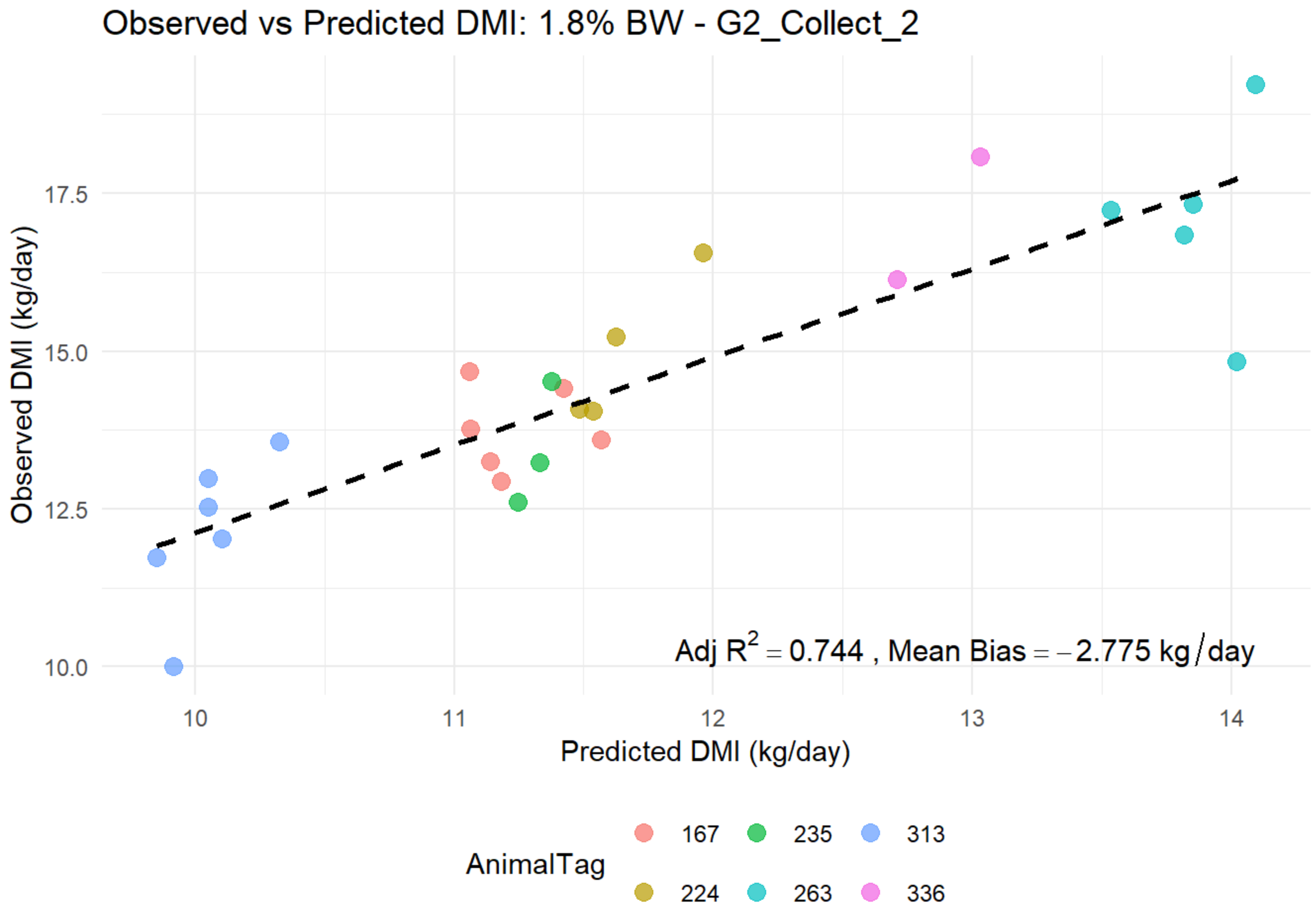

17A. Building a Predictive Model for DMI

Here we run model selection using corrected Akaike Information Criterion corrected (AICc) to determine the best covariate(s) to estimate DMI from each trial phases (G1 or G2 and G1 and G2 combined). Note that this process includes covariate removal due to multicollinearity. The code produces the adjusted R2 values and the best models for predicting DMI.

```
# # Step 1: Define AICc calculation function.
calculate_AICc <- function(model) {
  aic <- AIC(model)
  n <- length(model$residuals)
  k <- length(coef(model))
  aicc <- aic + (2 * k^2 + 2 * k) / (n - k - 1)
  return(aicc)
}

# # Step 2: Define model formulas.
formulas <- list(
  DMI_CH4      = DMI ~ Daily_Avg_CH4,
  DMI_CO2      = DMI ~ Daily_Avg_CO2,
  DMI_O2       = DMI ~ Daily_Avg_O2,
  DMI_AllGases = DMI ~ Daily_Avg_CH4 + Daily_Avg_CO2 + Daily_Avg_O2,
  DMI_All      = DMI ~ Daily_Avg_CH4 + Daily_Avg_CO2 + Daily_Avg_O2 + Weight
)
#
#
# # Step 3: Model fitting function.
fit_models <- function(data, formulas) {
  lapply(formulas, function(f) {
    model <- lm(f, data = data)
    list(
      model = model,
      AICc = calculate_AICc(model)
    )
  })
}
#
# # Step 4: Split data by trial period.
g1_data <- subset_data_clean %>% filter(Trial_Period == "G1_Collect")
g2_data <- subset_data_clean %>% filter(Trial_Period == "G2_Collect_2")
#
# # Step 5: Fit models.
models_g1 <- fit_models(g1_data, formulas)
models_g2 <- fit_models(g2_data, formulas)
models_combined <- fit_models(subset_data_clean, formulas)
#
# #Initial Variance Inflation Factor (VIF) testing.
# G1_Collect – model with all gases and weight
vif(models_g1$DMI_All$model)
```

```
## Daily_Avg_CH4 Daily_Avg_CO2  Daily_Avg_O2        Weight
##      8.196710     29.979381     25.058865      2.269381
```

```
#
# # G2_Collect_2 – model with all gases and weight
 vif(models_g2$DMI_All$model)
```

```
## Daily_Avg_CH4 Daily_Avg_CO2  Daily_Avg_O2        Weight
##      4.255390     15.421911      9.010877      1.108122
```

```
#
# # Combined — model with all gases and weight
 vif(models_combined$DMI_All$model)
```

```
## Daily_Avg_CH4 Daily_Avg_CO2  Daily_Avg_O2        Weight
##      5.370995     22.197944     12.211733      1.288642
```

```
#
# # Optional: VIF for model with only gases (no weight)
 vif(models_g1$DMI_AllGases$model)
```

```
## Daily_Avg_CH4 Daily_Avg_CO2  Daily_Avg_O2
##       4.99821      29.42664      20.31357
```

```
 vif(models_g2$DMI_AllGases$model)
```

```
## Daily_Avg_CH4 Daily_Avg_CO2  Daily_Avg_O2
##      4.082031     14.509846      8.869566
```

```
 vif(models_combined$DMI_AllGases$model)
```

```
## Daily_Avg_CH4 Daily_Avg_CO2  Daily_Avg_O2
##      5.370666     20.189162     10.991312
```

```
#
# # Step 6: Extract AICc
 extract_AICc <- function(model_list) {
   sapply(model_list, function(x) x$AICc)
 }
#
 cat("AICc - G1_Collect\n")
```

```
## AICc - G1_Collect
```

```
 print(extract_AICc(models_g1))
```

```
##      DMI_CH4      DMI_CO2       DMI_O2 DMI_AllGases      DMI_All
##     99.48332    103.89651    105.14346    101.97730    101.67840
```

```
#
 cat("\nAICc - G2_Collect_2\n")
```

```
##
## AICc - G2_Collect_2
```

```
 print(extract_AICc(models_g2))
```

```
##      DMI_CH4      DMI_CO2       DMI_O2 DMI_AllGases      DMI_All
##     159.4281     158.7570     159.6224     162.6085     129.4805
```

```
#
 cat("\nAICc - Combined\n")
```

```
##
## AICc - Combined
```

```
 print(extract_AICc(models_combined))
```

```
##      DMI_CH4      DMI_CO2       DMI_O2 DMI_AllGases      DMI_All
##     261.7743     262.9835     267.5757     262.5448     217.4429
```

```
#
# # Step 7: Extract tidy summaries
 model_summaries <- list(
   G1_Collect    = tidy(models_g1$DMI_CH4$model),
   G2_Collect_2  = tidy(models_g2$DMI_All$model),
   Combined      = tidy(models_combined$DMI_All$model)
 )
#
 print(model_summaries$G1_Collect)
```

```
## # A tibble: 2 × 5
##   term          estimate std.error statistic    p.value
##   <chr>            <dbl>     <dbl>     <dbl>      <dbl>
## 1 (Intercept)    19.2       2.94        6.51 0.00000720
## 2 Daily_Avg_CH4   0.0375    0.0135      2.77 0.0136
```

```
 print(model_summaries$G2_Collect_2)
```

```
## # A tibble: 5 × 5
##   term          estimate std.error statistic      p.value
##   <chr>            <dbl>     <dbl>     <dbl>        <dbl>
## 1 (Intercept)   -1.06      5.44       -0.196 0.847
## 2 Daily_Avg_CH4  0.00755   0.0154      0.491 0.629
## 3 Daily_Avg_CO2  0.00107   0.00153     0.699 0.492
## 4 Daily_Avg_O2  -0.00231   0.00195    -1.18  0.250
## 5 Weight         0.0249    0.00312     7.97  0.0000000868
```

```
print(model_summaries$Combined)
```

```
## # A tibble: 5 × 5
##   term           estimate std.error statistic  p.value
##   <chr>             <dbl>     <dbl>     <dbl>    <dbl>
## 1 (Intercept)   -6.48       3.84       -1.69  9.92e- 2
## 2 Daily_Avg_CH4  0.00617    0.0132      0.467 6.43e- 1
## 3 Daily_Avg_CO2  0.000498   0.00120     0.414 6.81e- 1
## 4 Daily_Avg_O2  -0.000353   0.00126    -0.281 7.80e- 1
## 5 Weight         0.0242     0.00277     8.73  1.04e-10
```

```
#
# # Step 8: VIF filtering function
# # Function to filter models by VIF threshold
 filter_models_by_vif <- function(model_list, threshold = 6) {
   filtered_models <- list()
#
   for (name in names(model_list)) {
     mod <- model_list[[name]]$model
#
#     # Extract predictor terms (not intercept)
     predictor_terms <- attr(terms(mod), "term.labels")
#
#     # If there is only 1 predictor (or none), skip VIF and retain the model
     if (length(predictor_terms) < 2) {
       filtered_models[[name]] <- model_list[[name]]
     } else {
       vif_values <- car::vif(mod)
       if (all(vif_values <= threshold)) {
         filtered_models[[name]] <- model_list[[name]]
       }
     }
   }
   return(filtered_models)
 }
#
# # ---- Step 9: Apply VIF Filtering ----
# # These filtered models account for multicollinearity using VIF.
# # Only models where all predictor variables have VIF ≤ 6 are retained.
# # This ensures the predictors are not highly collinear, improving model interpretability and s
tability.
 models_g1_filtered <- filter_models_by_vif(models_g1)
 models_g2_filtered <- filter_models_by_vif(models_g2)
 models_combined_filtered <- filter_models_by_vif(models_combined)
#
# # ---- Step 10: Interpret AICc Results ----
# # From the VIF-filtered models, the model with the **lowest AICc** is considered the best.
# # AICc (corrected Akaike Information Criterion) balances model fit and complexity.
# # This approach is appropriate for this dataset, as it contains fewer than 5000 observations —
# # making AICc a more reliable criterion than AIC in small samples.
 cat("\nModels retained for G1_Collect:\n")
```

```
##
## Models retained for G1_Collect:
```

```
 print(names(models_g1_filtered))
```

```
## [1] "DMI_CH4" "DMI_CO2" "DMI_O2"
```

```
#
 cat("\nModels retained for G2_Collect_2:\n")
```

```
##
## Models retained for G2_Collect_2:
```

```
 print(names(models_g2_filtered))
```

```
## [1] "DMI_CH4" "DMI_CO2" "DMI_O2"
```

```
#
 cat("\nModels retained for Combined:\n")
```

```
##
## Models retained for Combined:
```

```
 print(names(models_combined_filtered))
```

```
## [1] "DMI_CH4" "DMI_CO2" "DMI_O2"
```

```
#
# #Step 11:Display best models for each Trial Period
 best_models <- list(
   G1_Collect = models_g1_filtered$DMI_CH4$model,
   G2_Collect_2 = models_g2_filtered$DMI_CO2$model,
   Combined = models_combined_filtered$DMI_CH4$model
 )
#
# # Output tidy summaries for each
 model_summaries_best <- lapply(best_models, tidy)
#
# # Print each summary
 cat("\n--- G1_Collect: DMI ~ Daily_Avg_CH4 ---\n")
```

```
##
## --- G1_Collect: DMI ~ Daily_Avg_CH4 ---
```

```
 print(model_summaries_best$G1_Collect)
```

```
## # A tibble: 2 × 5
##   term          estimate std.error statistic    p.value
##   <chr>            <dbl>     <dbl>     <dbl>      <dbl>
## 1 (Intercept)    19.2       2.94        6.51 0.00000720
## 2 Daily_Avg_CH4   0.0375    0.0135      2.77 0.0136
```

```
#
 cat("\n--- G2_Collect_2: DMI ~ Daily_Avg_CO2 ---\n")
```

```
##
## --- G2_Collect_2: DMI ~ Daily_Avg_CO2 ---
```

```
 print(model_summaries_best$G2_Collect_2)
```

```
## # A tibble: 2 × 5
##   term           estimate std.error statistic  p.value
##   <chr>             <dbl>     <dbl>     <dbl>    <dbl>
## 1 (Intercept)   24.8       6.16          4.02 0.000501
## 2 Daily_Avg_CO2  0.000879  0.000755      1.16 0.256
```

```
#
 cat("\n--- Combined: DMI ~ Daily_Avg_CH4 ---\n")
```

```
##
## --- Combined: DMI ~ Daily_Avg_CH4 ---
```

```
 print(model_summaries_best$Combined)
```

```
## # A tibble: 2 × 5
##   term          estimate std.error statistic  p.value
##   <chr>            <dbl>     <dbl>     <dbl>    <dbl>
## 1 (Intercept)    21.9      2.52         8.67 6.68e-11
## 2 Daily_Avg_CH4   0.0325   0.00987      3.29 2.01e- 3
```

```
#
# #Step 11: Output adjusted R2 values
# # Extract adjusted R² values for the best models
 adj_r2_values <- list(
   G1_Collect = summary(models_g1_filtered$DMI_CH4$model)$adj.r.squared,
   G2_Collect_2 = summary(models_g2_filtered$DMI_CO2$model)$adj.r.squared,
   Combined = summary(models_combined_filtered$DMI_CH4$model)$adj.r.squared
 )
#
# # Print the adjusted R² values
 cat("\nAdjusted R² values for best models:\n")
```

```
##
## Adjusted R² values for best models:
```

```
 cat("G1_Collect (DMI ~ CH4):       ", round(adj_r2_values$G1_Collect, 4), "\n")
```

```
## G1_Collect (DMI ~ CH4):        0.2825
```

```
cat("G2_Collect_2 (DMI ~ CO2):     ", round(adj_r2_values$G2_Collect_2, 4), "\n")
```

```
## G2_Collect_2 (DMI ~ CO2):      0.0139
```

```
cat("Combined (DMI ~ CH4):         ", round(adj_r2_values$Combined, 4), "\n")
```

```
## Combined (DMI ~ CH4):          0.1863
```

17B. Plot Regressions Models

Plot the best models for predicting DMI for each trail phases and combined.

```
# Add a column to each dataset for grouping.
g1_data$Group <- "G1_Collect"
g2_data$Group <- "G2_Collect_2"
subset_data_clean$Group <- "Combined"

# Calculate adjusted R² for each best model.
r2_g1 <- summary(models_g1_filtered$DMI_CH4$model)$adj.r.squared
r2_g2 <- summary(models_g2_filtered$DMI_CO2$model)$adj.r.squared
r2_combined <- summary(models_combined_filtered$DMI_CH4$model)$adj.r.squared

plot_model <- function(data, xvar, yvar = "DMI", group_label, r2_value) {
  ggplot(data, aes(x = .data[[xvar]], y = .data[[yvar]])) +
    geom_point(color = "darkblue", size = 2) +
    geom_smooth(method = "lm", se = TRUE, color = "red") +
    labs(
      title = sprintf("%s: %s ~ %s", group_label, yvar, xvar),
      x = xvar,
      y = yvar
    ) +
    annotate(
      "text",
      x = Inf, y = -Inf,
      label = bquote(Adjusted~R^2 == .(round(r2_value, 3))),
      hjust = 1.1, vjust = -1.1,
      size = 5, color = "black"
    ) +
    theme_minimal(base_size = 14)
}

# Calculate Adjusted R² values
r2_g1       <- summary(models_g1_filtered$DMI_CH4$model)$adj.r.squared
r2_g2       <- summary(models_g2_filtered$DMI_CO2$model)$adj.r.squared
r2_combined <- summary(models_combined_filtered$DMI_CH4$model)$adj.r.squared

# Generate plots
plot1 <- plot_model(g1_data, "Daily_Avg_CH4", group_label = "G1_Collect",     r2_value = r2_g1)
plot2 <- plot_model(g2_data, "Daily_Avg_CO2", group_label = "G2_Collect_2",   r2_value = r2_g2)
plot3 <- plot_model(subset_data_clean, "Daily_Avg_CH4", group_label = "Combined", r2_value = r2_
combined)

# Display
print(plot1)
```

```
## `geom_smooth()` using formula = 'y ~ x'
```

```
## Warning in is.na(x): is.na() applied to non-(list or vector) of type 'language'
```

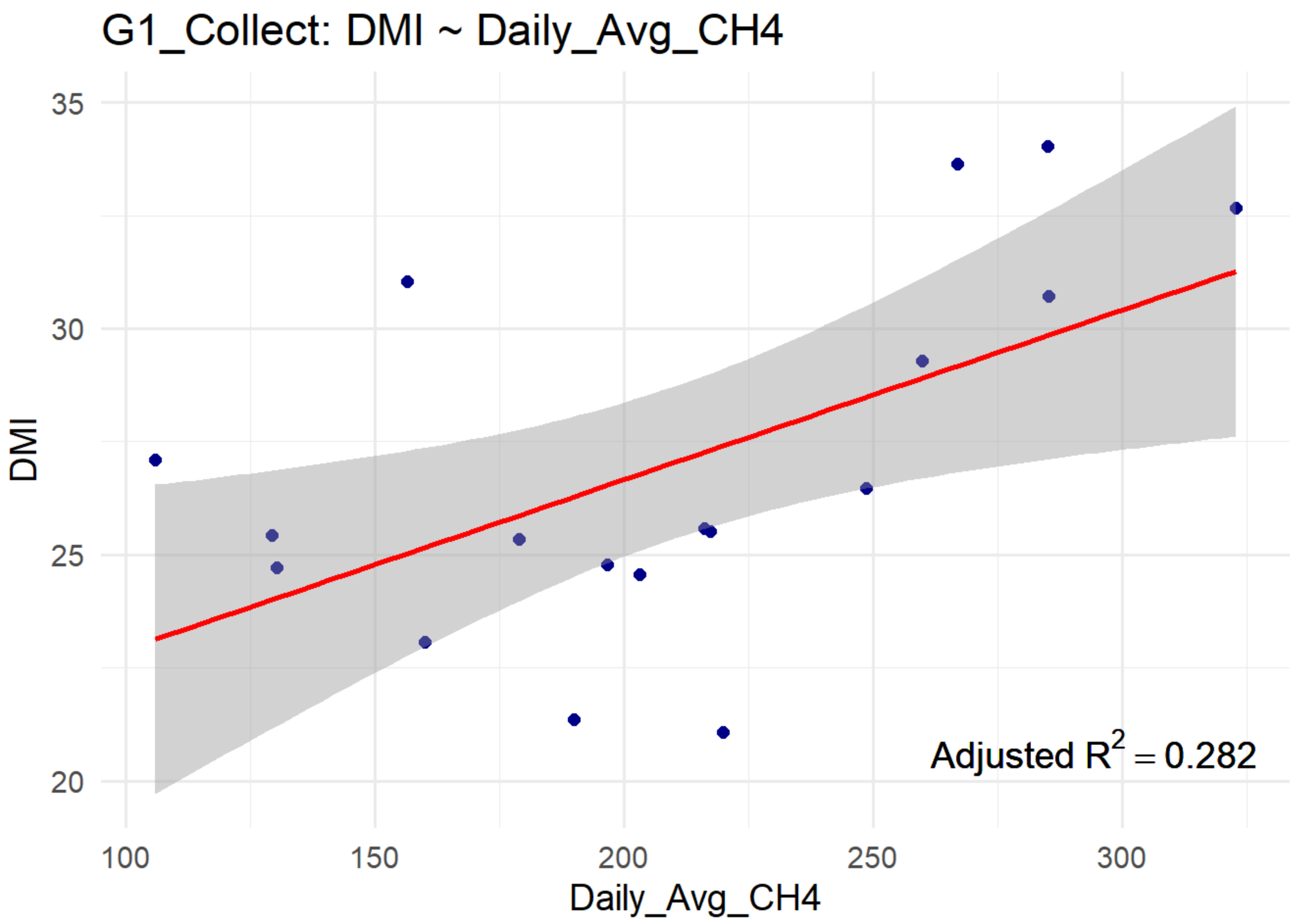


```
print(plot2)
```

```
## `geom_smooth()` using formula = 'y ~ x'
```

```
## Warning in is.na(x): is.na() applied to non-(list or vector) of type 'language'
```

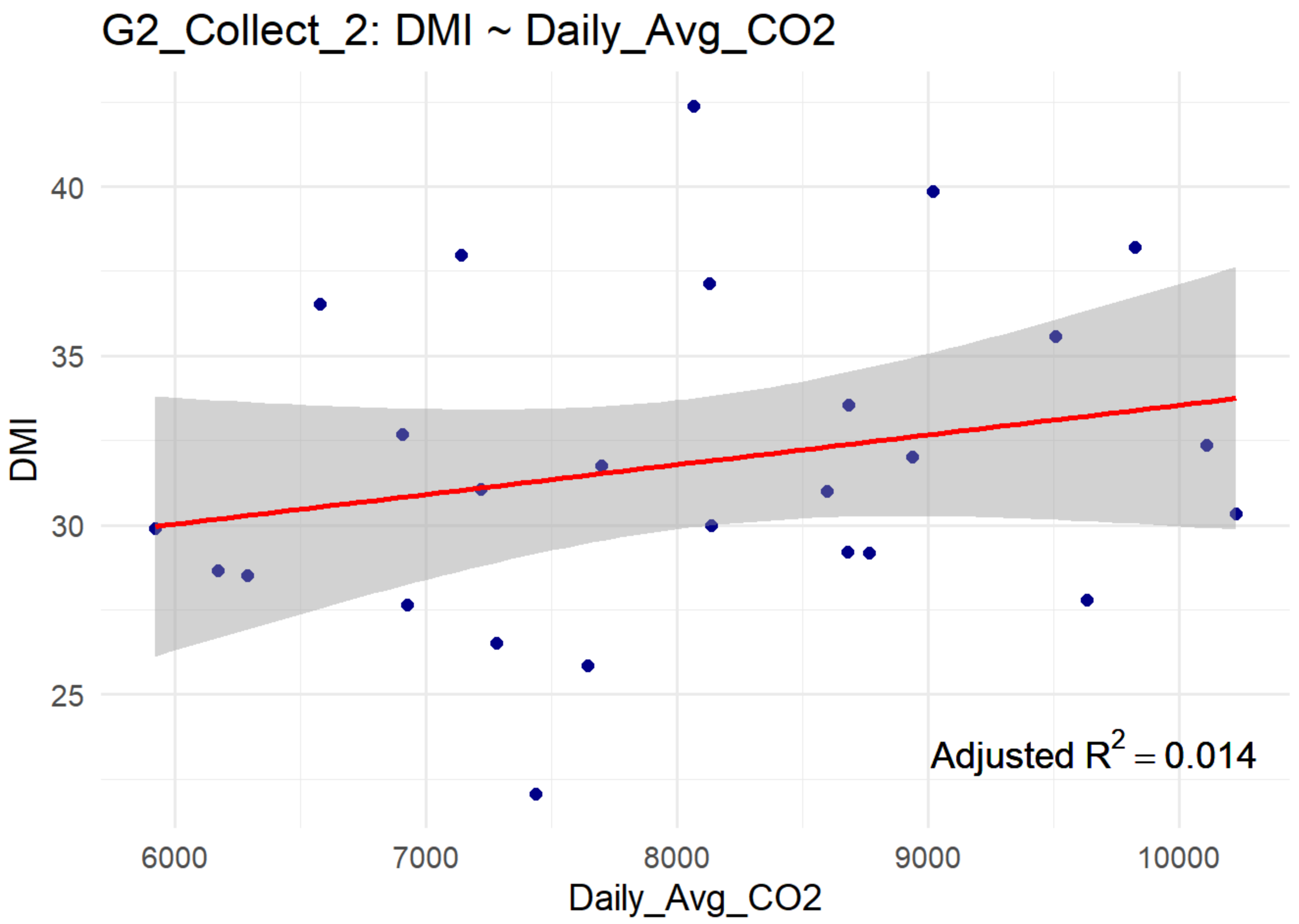


```
print(plot3)
```

```
## `geom_smooth()` using formula = 'y ~ x'
```

```
## Warning in is.na(x): is.na() applied to non-(list or vector) of type 'language'
```

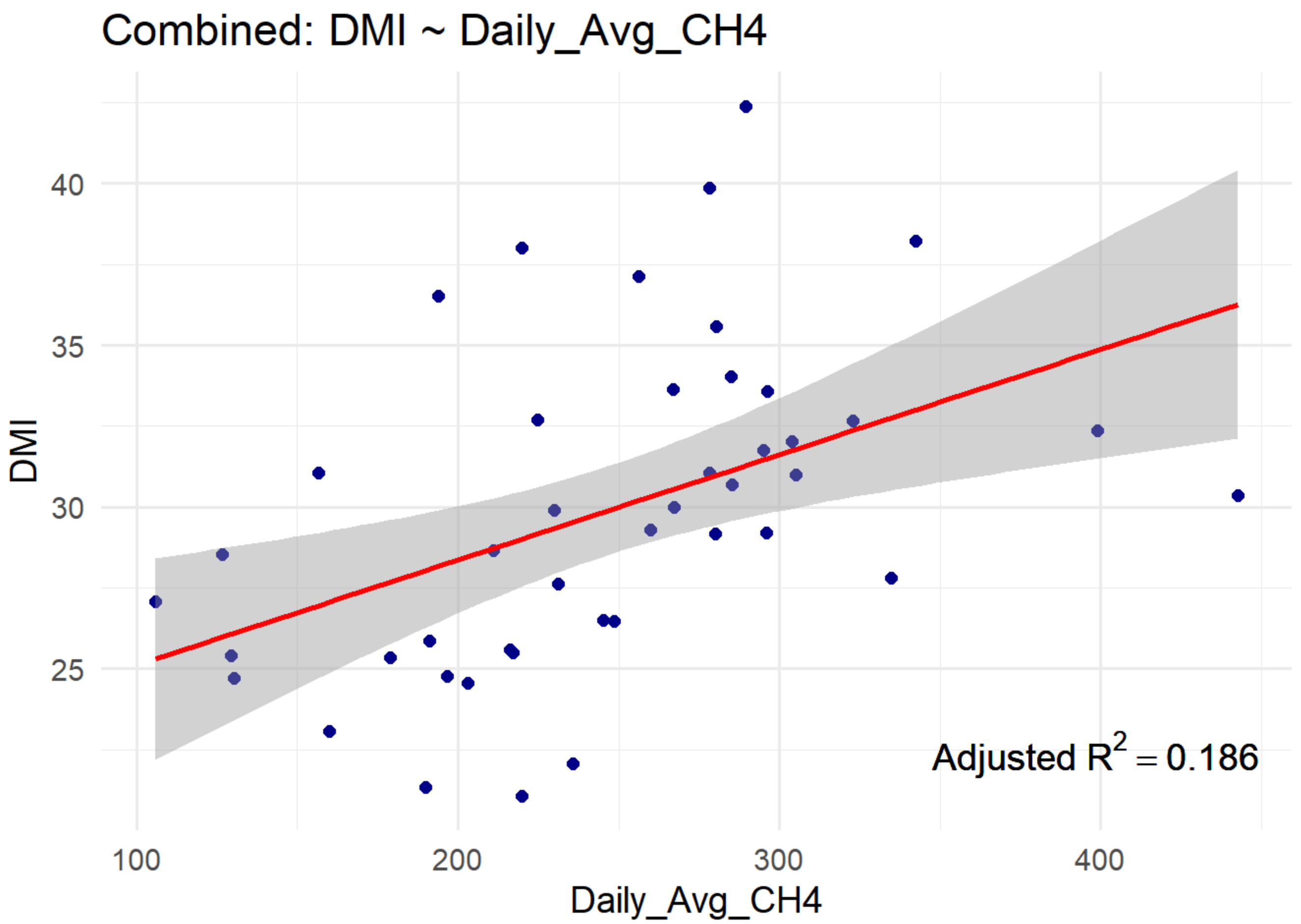

##18A. Data Smoothing Process

Here we bring back the entire data set to and apply an exponential smoothing function (like what dynamic modeling programs use such as Vensim DSS). The data are first averaged across all seven cows to give a herd average. If only one cow had an observation on a particular day then its individual value is used in the herd average data (step 1). The data is smoothing over a seven-day period. New variables such as DMI_R are added to the data frame (see step 4). We then plot of subset of G1 and G2 combined to demonstrate differences in original versus smoothed data (see step 7).

```
# 1) Compute daily herd means from clean_data_no
daily_herd_avg <- clean_data_no %>%
  group_by(Trial_Period, Date) %>%
  summarise(
    herd_DMI = mean(DMI,                 na.rm = TRUE),
    herd_CH4 = mean(Daily_Avg_CH4,       na.rm = TRUE),
    herd_CO2 = mean(Daily_Avg_CO2,       na.rm = TRUE),
    herd_O2  = mean(Daily_Avg_O2,        na.rm = TRUE),
    .groups  = "drop"
  )

# 2) Index each phase with a Day counter
daily_herd_indexed <- daily_herd_avg %>%
  arrange(Trial_Period, Date) %>%
  group_by(Trial_Period) %>%
  mutate(Day = row_number()) %>%
  ungroup()

# 3) Define Vensim-style SMOOTH
smooth_vensim <- function(x, tau, dt = 1) {
  alpha <- dt / tau
  y     <- numeric(length(x))
  y[1]  <- x[1]
  for (t in 2:length(x)) {
    y[t] <- y[t-1] + alpha * (x[t] - y[t-1])
  }
  y
}
tau_days <- 7

# 4) Apply smoothing to every phase
smoothed_all <- daily_herd_indexed %>%
  group_by(Trial_Period) %>%
  mutate(
    DMI_R = smooth_vensim(herd_DMI, tau_days),
    CH4_R = smooth_vensim(herd_CH4, tau_days),
    CO2_R = smooth_vensim(herd_CO2, tau_days),
    O2_R  = smooth_vensim(herd_O2,  tau_days)
  ) %>%
  ungroup()

# 5) Build the Combined_G1_G2 series *after* smoothing each day's raw mean
combined <- smoothed_all %>%
  filter(Trial_Period %in% c("G1_Collect","G2_Collect_2")) %>%
  group_by(Date) %>%
  summarise(
    herd_DMI = mean(herd_DMI, na.rm=TRUE),
    herd_CH4 = mean(herd_CH4, na.rm=TRUE),
    herd_CO2 = mean(herd_CO2, na.rm=TRUE),
    herd_O2  = mean(herd_O2,  na.rm=TRUE),
    .groups = "drop"
  ) %>%
```

```
  arrange(Date) %>%
  mutate(
    Day   = row_number(),
    DMI_R = smooth_vensim(herd_DMI, tau_days),
    CH4_R = smooth_vensim(herd_CH4, tau_days),
    CO2_R = smooth_vensim(herd_CO2, tau_days),
    O2_R  = smooth_vensim(herd_O2,  tau_days),
    Trial_Period = "Combined_G1_G2"
  )

# 6) Bind & filter to three series
to_plot <- bind_rows(smoothed_all, combined) %>%
  filter(Trial_Period %in% c("G1_Collect","G2_Collect_2","Combined_G1_G2"))

# 7) Plot raw vs. smoothed DMI, faceted
ggplot(to_plot, aes(x = Day)) +
  geom_line(aes(y = herd_DMI), color = "grey70") +
  geom_line(aes(y = DMI_R),     color = "steelblue", linetype = "dashed") +
  facet_wrap(~ Trial_Period, scales = "free_y") +
  labs(
    title    = "Raw vs. Exponentially-Smoothed Herd DMI",
    subtitle = "G1_Collect, G2_Collect_2 and Combined_G1_G2",
    x        = "Day index",
    y        = "DMI (g/day)"
  ) +
  theme_minimal()
```

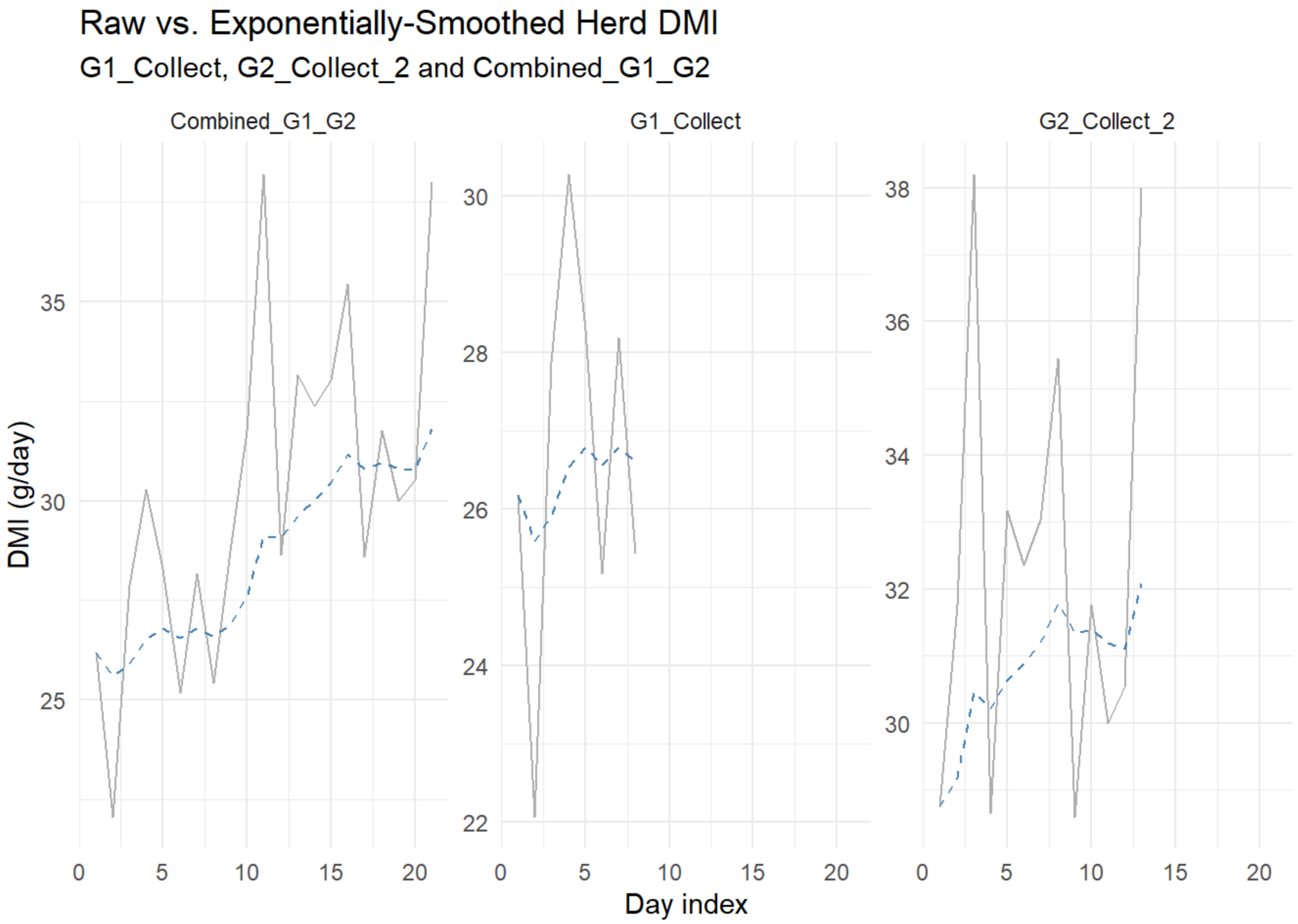

####

##18B. Additional Plots

See more detailed plots for each variable (DMI, CH4, CO2, and O2).

```
# 1) Pull out just the Combined series
combined <- to_plot %>%
  filter(Trial_Period == "Combined_G1_G2")

# 2) Helper to make each plot
make_plot <- function(df, raw, smooth, ylab, title){
  ggplot(df, aes(x = Day)) +
    geom_line(aes(y = .data[[raw]]),   color = "grey70", size = 0.5) +
    geom_line(aes(y = .data[[smooth]]), color = "steelblue", size = 1) +
    labs(title = title,
         x     = "Day",
         y     = ylab) +
    theme_minimal()
}

# 3) Four plots
p_dmi <- make_plot(combined,
                   raw    = "herd_DMI",
                   smooth = "DMI_R",
                   ylab   = "DMI (g/day)",
                   title  = "Combined DMI: Raw vs. Smoothed")
```

```
## Warning: Using `size` aesthetic for lines was deprecated in ggplot2 3.4.0.
## ℹ Please use `linewidth` instead.
## This warning is displayed once every 8 hours.
## Call `lifecycle::last_lifecycle_warnings()` to see where this warning was
## generated.
```

```
p_ch4 <- make_plot(combined,
                   raw    = "herd_CH4",
                   smooth = "CH4_R",
                   ylab   = expression(CH[4]~"(g/day)"),
                   title  = "Combined Methane: Raw vs. Smoothed")



p_co2 <- make_plot(combined,
                   raw    = "herd_CO2",
                   smooth = "CO2_R",
                   ylab   = expression(CO[2]~"(g/day)"),
                   title  = "Combined Carbon Dioxide: Raw vs. Smoothed")

p_o2  <- make_plot(combined,
                   raw    = "herd_O2",
                   smooth = "O2_R",
                   ylab   = expression(O[2]~"(g/day)"),
                   title  = "Combined Oxygen: Raw vs. Smoothed")

# 4) Display
print(p_dmi)
```

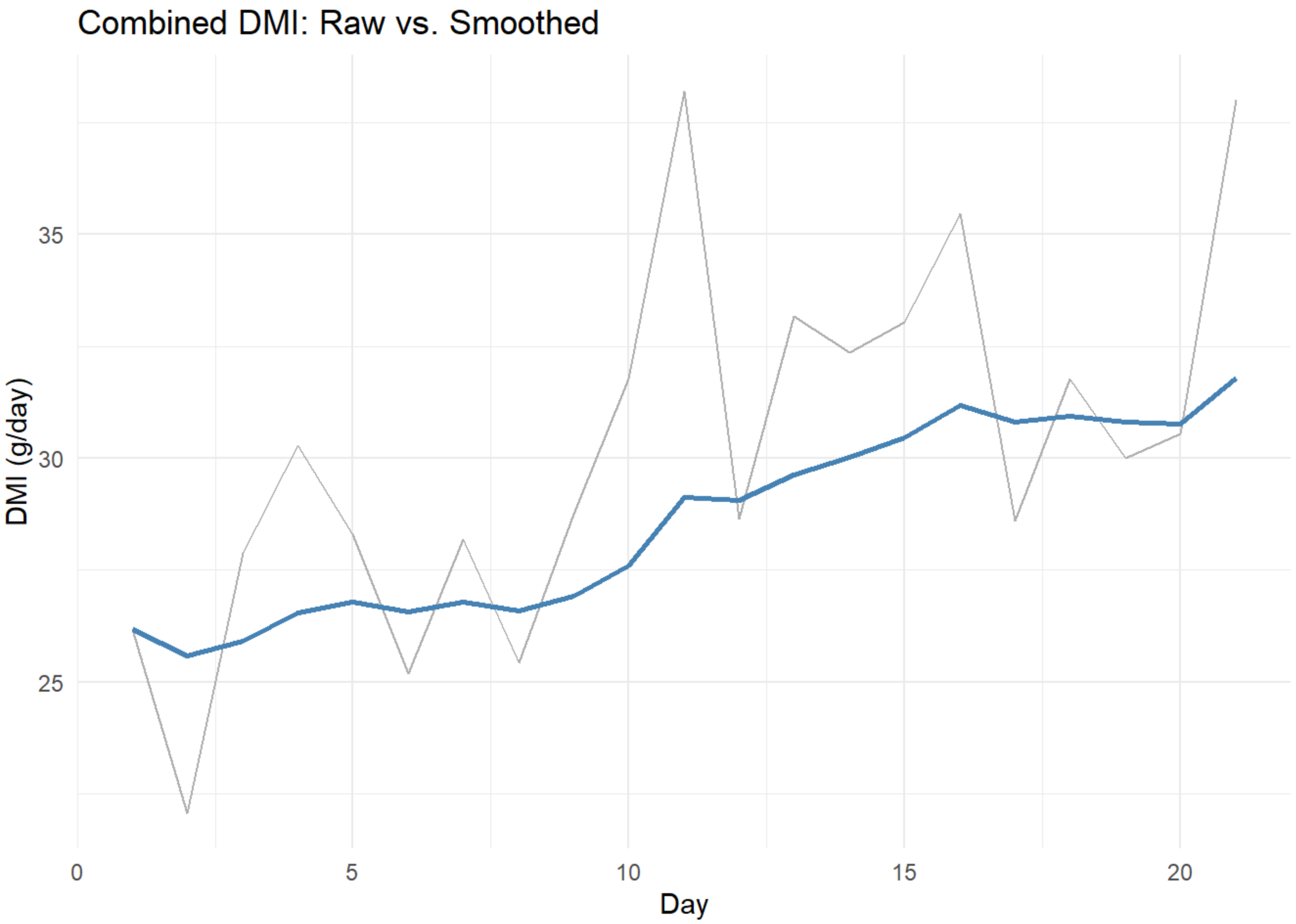


```
print(p_ch4)
```

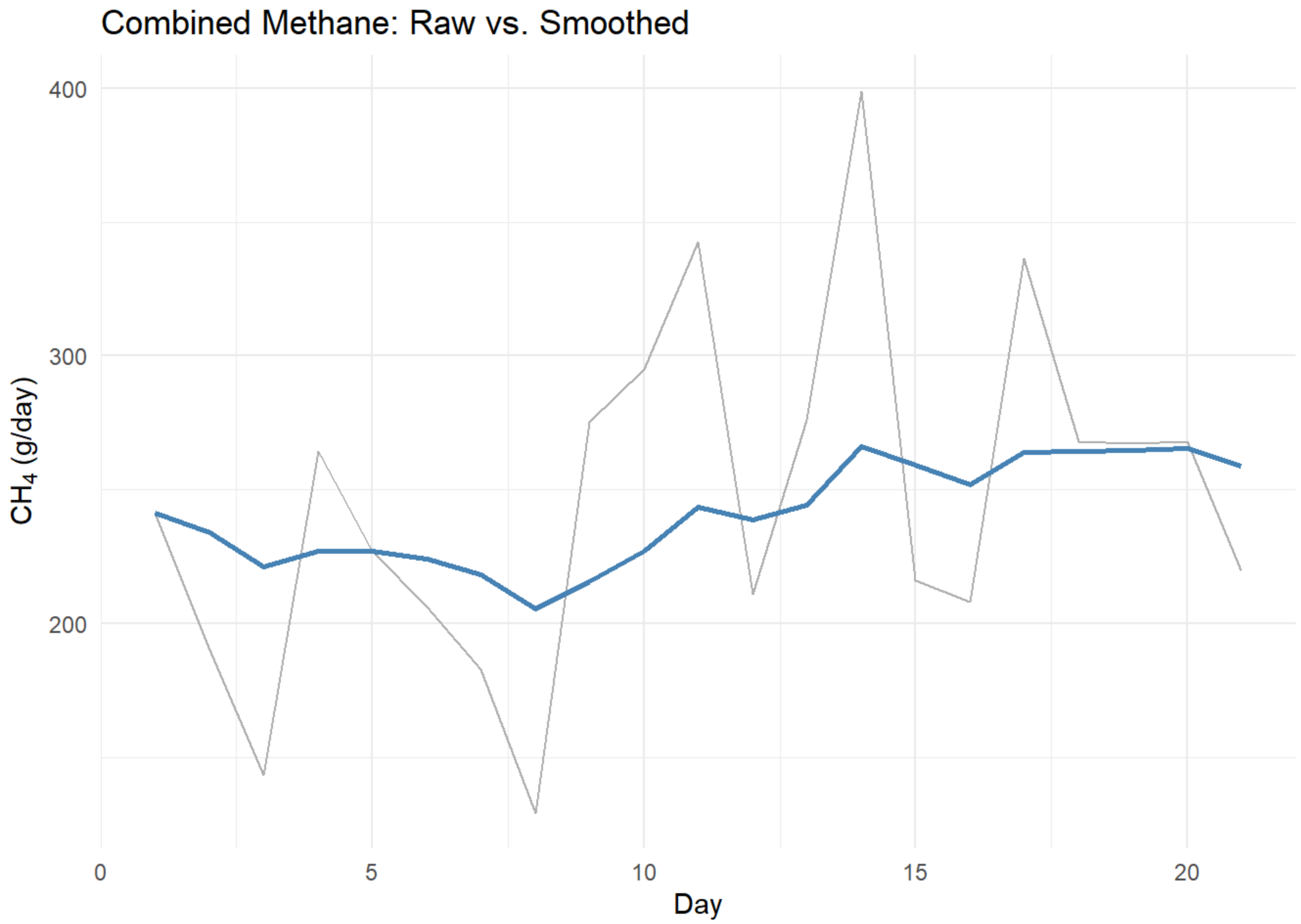
Combined Methane: Raw vs. Smoothed
400
300
200
CH4 (g/day)
0
5
10
15
20
Day

```
print(p_co2)
```

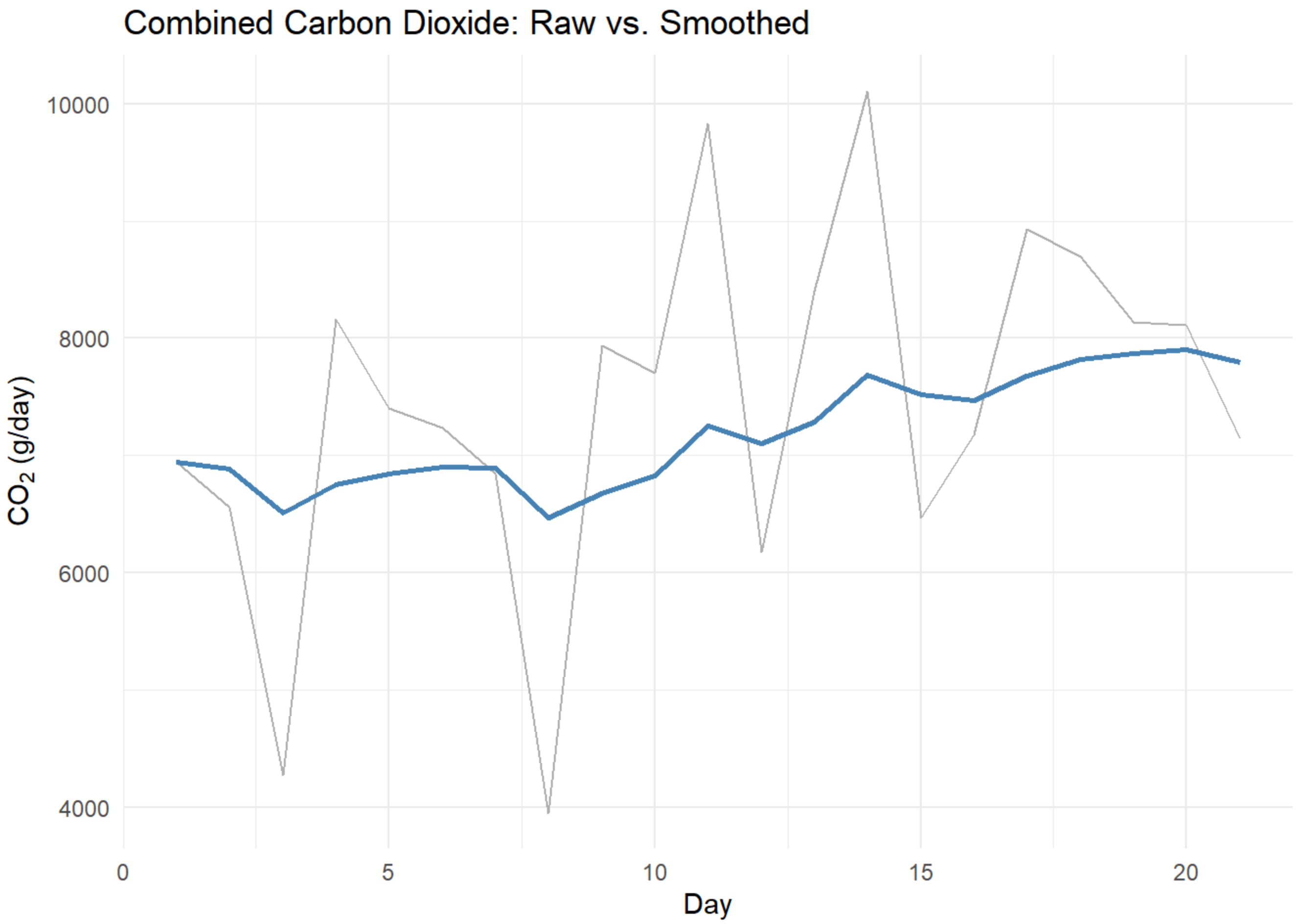
Combined Carbon Dioxide: Raw vs. Smoothed
10000
8000
6000
4000
CO2 (g/day)
0
5
10
15
20
Day

```
print(p_o2)
```

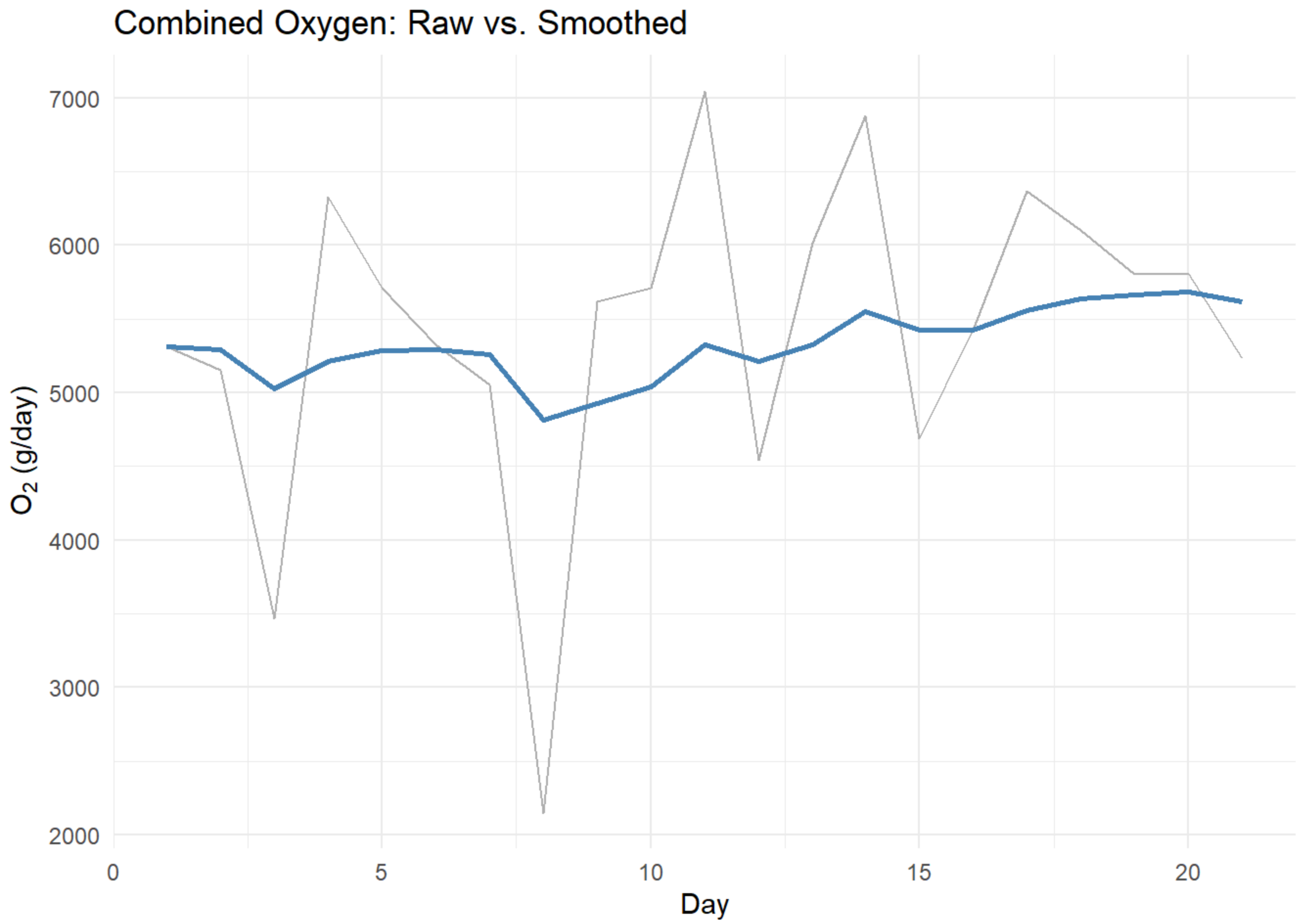

18C. The smoothed-herd average data is now redeployed using our predictive DMI models for each trail phase and combined (reference section 17 above). Note that since we started from the "clean_data_no" data frame we have to convert

DMI_R from lb to kg. Similar to section 17 we plot and report adjusted R2 and mean bias for our new estimates using the smoothed data.

```
#herd_smoothed

# 0) Convert DMI_R from lb/day to kg/day
smoothed_all <- smoothed_all %>%
  mutate(
    DMI_R = DMI_R * 0.453592   # now in kg/day
  )

# Split smoothed data into groups
herd_g1 <- smoothed_all %>% filter(Trial_Period == "G1_Collect")
herd_g2 <- smoothed_all %>% filter(Trial_Period == "G2_Collect_2")
herd_combined <-smoothed_all %>%
  filter(Trial_Period %in% c("G1_Collect", "G2_Collect_2"))

# Step 3: Fit best models on smoothed herd-level data
model_g1_herd <- lm(DMI_R ~ CH4_R, data = herd_g1)
model_g2_herd <- lm(DMI_R ~ CO2_R, data = herd_g2)

model_combined_herd <- lm(DMI_R ~ CH4_R, data = herd_combined)

# Step 4: Get Adjusted R² and summary
summary_g1 <- summary(model_g1_herd)
summary_g2 <- summary(model_g2_herd)
summary_combined <- summary(model_combined_herd)

cat("Adjusted R² - G1_Collect:\n", summary_g1$adj.r.squared, "\n")
```

```
## Adjusted R² - G1_Collect:
##  0.06583638
```

```
cat("Adjusted R² - G2_Collect_2:\n", summary_g2$adj.r.squared, "\n")
```

```
## Adjusted R² - G2_Collect_2:
##  -0.0018253
```

```
cat("Adjusted R² - Combined:\n", summary_combined$adj.r.squared, "\n")
```

```
## Adjusted R² - Combined:
##  0.7682564
```

```
# Step 5: Predict DMI. I.e., Deploy the model using data.
herd_g1 <- herd_g1 %>%
  mutate(DMI_pred = predict(model_g1_herd, newdata = herd_g1),
         Bias = DMI_pred - DMI_R)

herd_g2 <- herd_g2 %>%
  mutate(DMI_pred = predict(model_g2_herd, newdata = herd_g2),
         Bias = DMI_pred - DMI_R)

herd_combined <- herd_combined %>%
  mutate(DMI_pred = predict(model_combined_herd, newdata = herd_combined),
         Bias = DMI_pred - DMI_R)

# Step 6: Compute Mean Bias
cat("Mean Bias - G1_Collect:\n", mean(herd_g1$Bias, na.rm = TRUE), "\n")
```

```
## Mean Bias - G1_Collect:
##  -3.774758e-15
```

```
cat("Mean Bias - G2_Collect_2:\n", mean(herd_g2$Bias, na.rm = TRUE), "\n")
```

```
## Mean Bias - G2_Collect_2:
##  -1.366437e-15
```

```
cat("Mean Bias - Combined:\n", mean(herd_combined$Bias, na.rm = TRUE), "\n")
```

```
## Mean Bias - Combined:
##  -1.522576e-15
```

```r
# # Step 7: Plot observed vs predicted DMI for each group

# --- Precompute metrics & subtitle expressions ---
# G1
adjr2_g1 <- summary(model_g1_herd)$adj.r.squared
bias_g1  <- mean(herd_g1$Bias, na.rm = TRUE)
subtitle_g1 <- bquote(
  Adj~R^2 == .(round(adjr2_g1,3))
  ~ ";" ~
  Mean~Bias == .(round(bias_g1,2))~kg
)

# G2
adjr2_g2 <- summary(model_g2_herd)$adj.r.squared
bias_g2  <- mean(herd_g2$Bias, na.rm = TRUE)
subtitle_g2 <- bquote(
  Adj~R^2 == .(round(adjr2_g2,3))
  ~ ";" ~
  Mean~Bias == .(round(bias_g2,2))~kg
)

# Combined
adjr2_c <- summary(model_combined_herd)$adj.r.squared
bias_c  <- mean(herd_combined$Bias, na.rm = TRUE)
subtitle_c <- bquote(
  Adj~R^2 == .(round(adjr2_c,3))
  ~ ";" ~
  Mean~Bias == .(round(bias_c,2))~kg
)

# --- Plot for G1_Collect (DMI_R ~ CH4_R) ---
p_g1 <- ggplot(herd_g1, aes(x = CH4_R, y = DMI_R)) +
  geom_point(color = "forestgreen", size = 2) +
  geom_smooth(method = "lm", se = TRUE, color = "black") +
  labs(
    title    = "G1_Collect: Smoothed DMI vs Methane",
    subtitle = subtitle_g1,
    x        = expression(CH[4]~" (smoothed g/d)"),
    y        = "DMI (smoothed kg/d)"
  ) +
  theme_minimal(base_size = 14)

# --- Plot for G2_Collect_2 (DMI_R ~ CO2_R) ---
p_g2 <- ggplot(herd_g2, aes(x = CO2_R, y = DMI_R)) +
  geom_point(color = "darkorange", size = 2) +
  geom_smooth(method = "lm", se = TRUE, color = "black") +
  labs(
    title    = "G2_Collect_2: Smoothed DMI vs Carbon Dioxide",
    subtitle = subtitle_g2,
    x        = expression(CO[2]~" (smoothed g/d)"),
    y        = "DMI (smoothed kg/d)"
  ) +
```

```
  theme_minimal(base_size = 14)

# --- Plot for Combined (DMI_R ~ CH4_R over both phases) ---
p_combined <- ggplot(herd_combined, aes(x = CH4_R, y = DMI_R)) +
  geom_point(color = "steelblue", size = 2) +
  geom_smooth(method = "lm", se = TRUE, color = "black") +
  labs(
    title    = "Combined (G1+G2): Smoothed DMI vs Methane",
    subtitle = subtitle_c,
    x        = expression(CH[4]~" (smoothed g/d)"),
    y        = "DMI (smoothed kg/d)"
  ) +
  theme_minimal(base_size = 14)

# --- Display them ---
print(p_g1)
```

```
## `geom_smooth()` using formula = 'y ~ x'
```

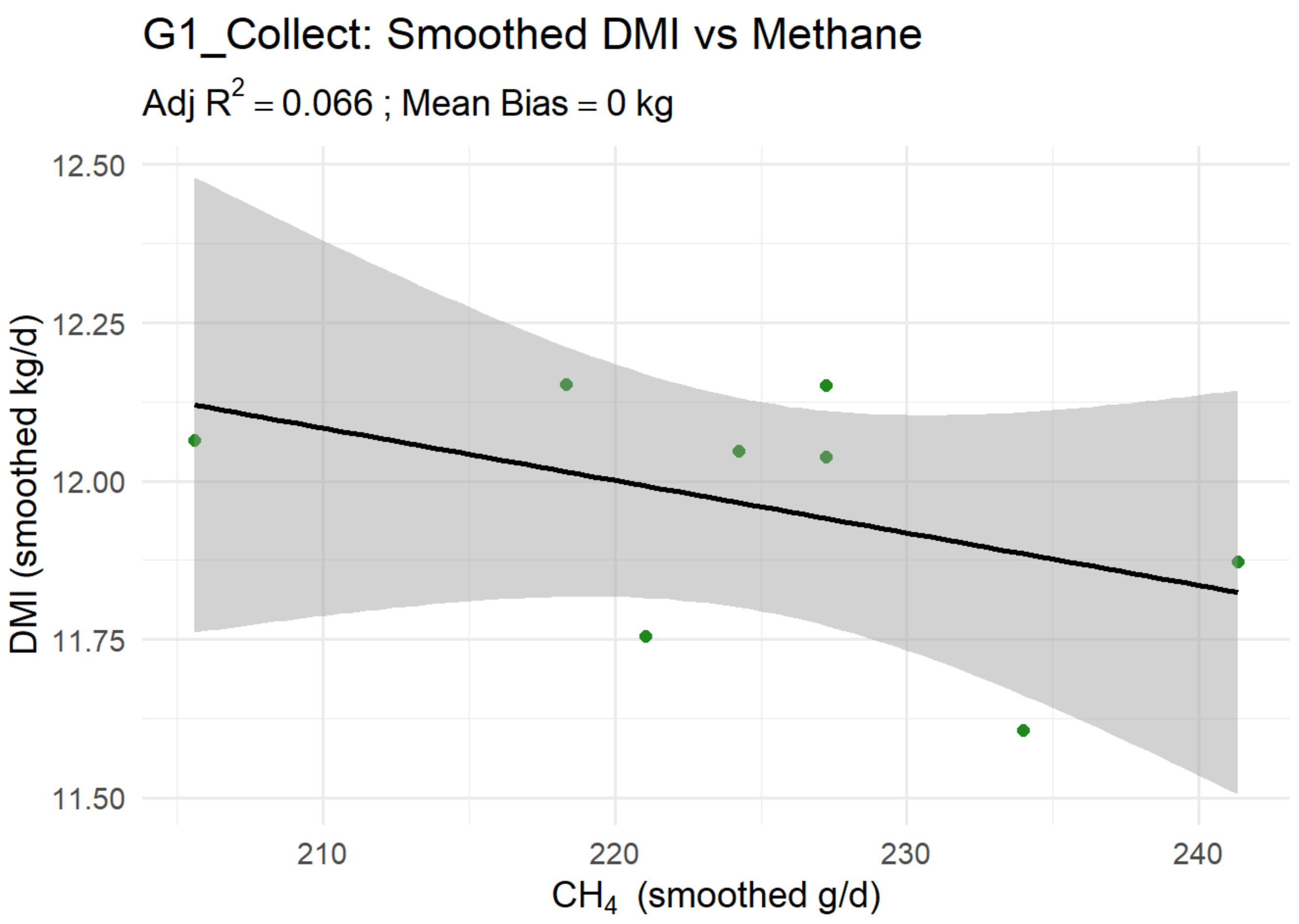


```
print(p_g2)
```

```
## `geom_smooth()` using formula = 'y ~ x'
```

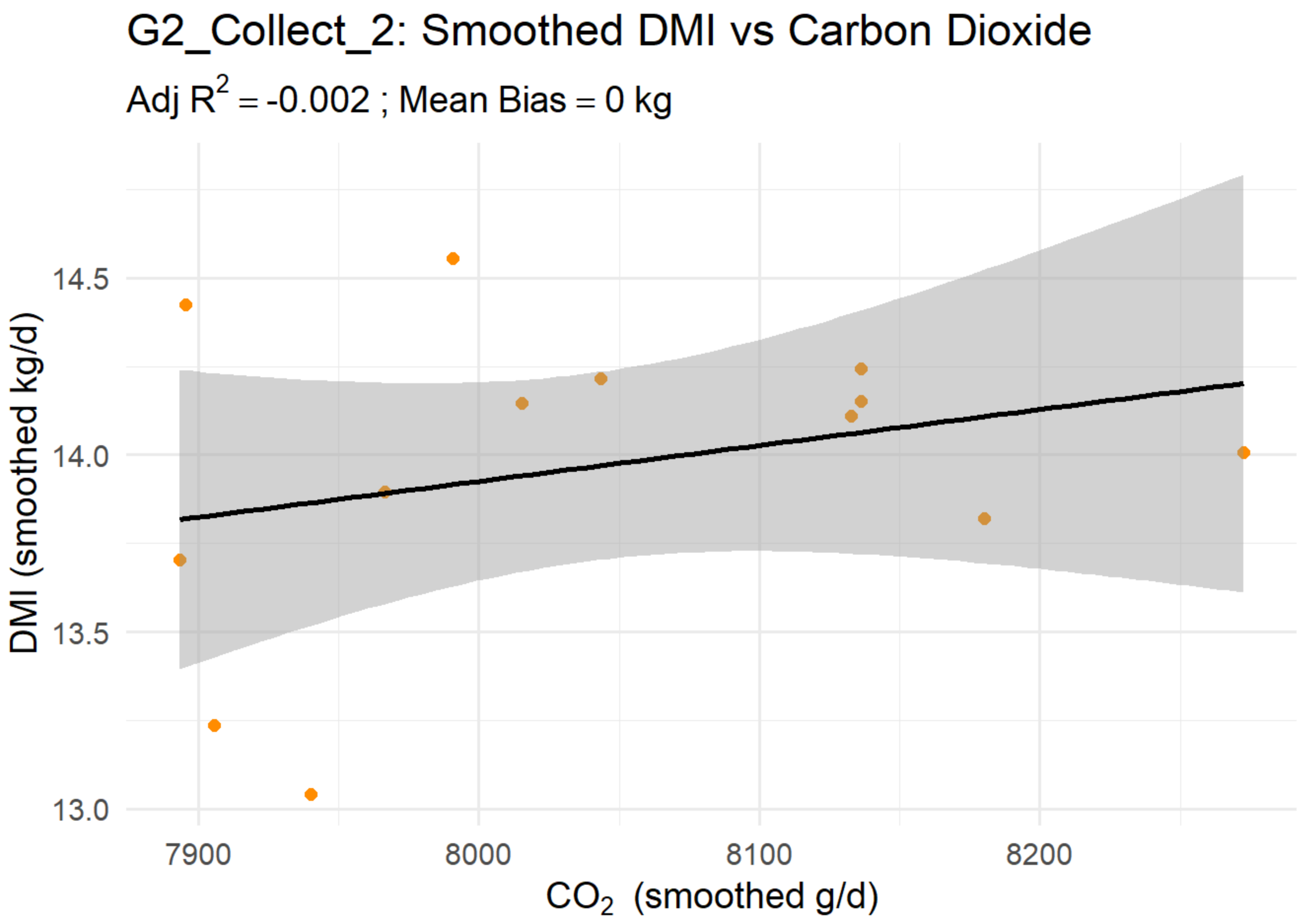


```
print(p_combined)
```

```
## `geom_smooth()` using formula = 'y ~ x'
```

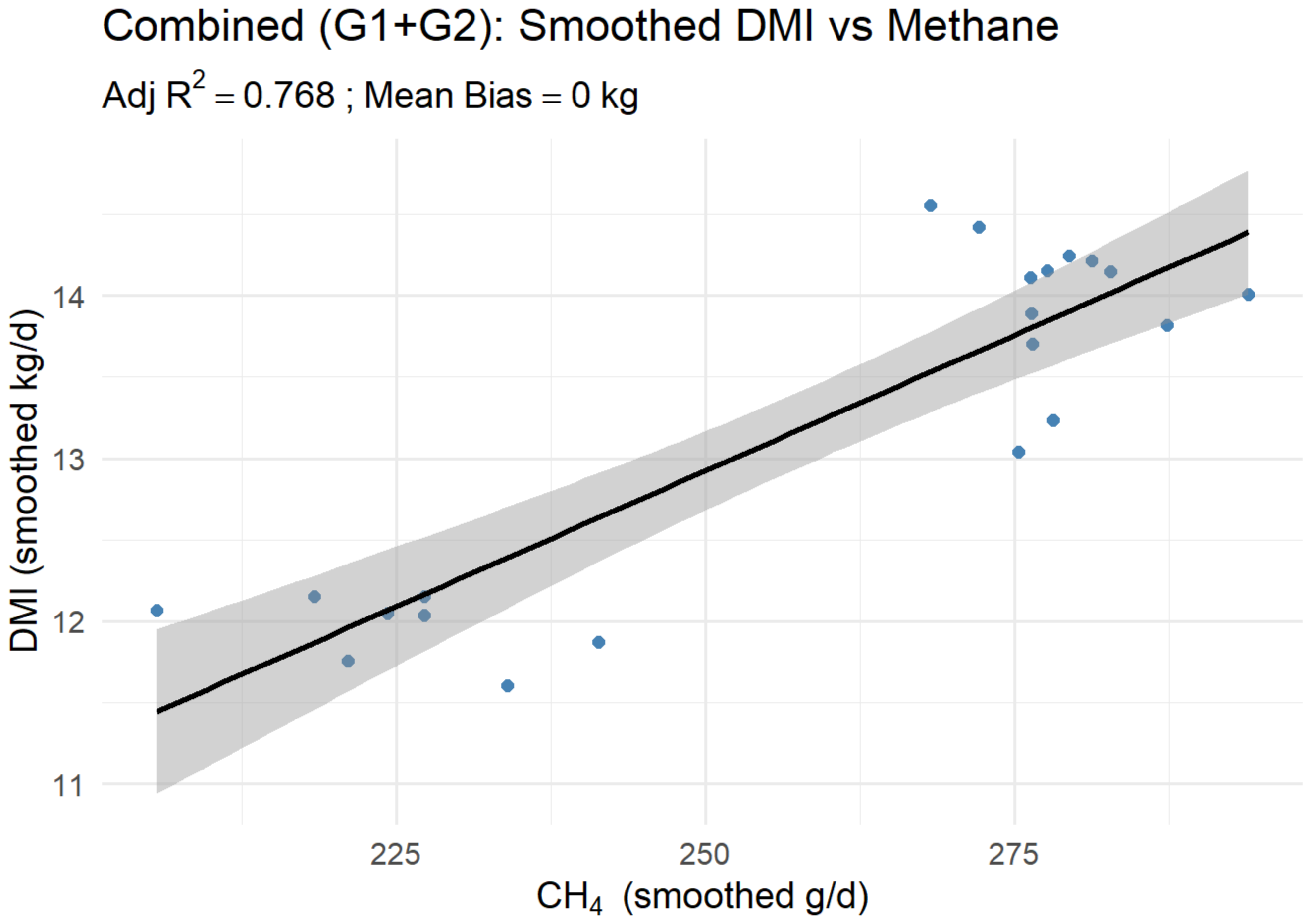

19. Calculate GreenFeed Pellet Crude Protien

This code allows for the calculation of crude protein contribution to basal diet from pellets used in the GreenFeed. The user may download individual drops per animal and adjust this code to estimate pellet CP contribution for each animal throughout the study period. The individual pellet data is not included in the current study and is available upon request.

```
# Step 1: Define input constants
feeding_periods <- 5                   # Feedings per day
drops_per_period <- 8                 # Drops per feeding
drop_mass_g <- 35                     # Mass per drop in grams
pellet_moisture_pct <- 12            # Moisture percentage
pellet_cp_pct <- 15                  # CP % of the pellet
bw_kg <- 622                         # Body weight in kg
dmi_pct_bw <- 1.8                    # % DMI as BW
basal_cp_pct <- 5.6                  # Basal diet CP %

# Step 2: Calculate pellet delivery
max_pellets_fed_g <- feeding_periods * drops_per_period * drop_mass_g  # Total grams
max_pellets_fed_kg <- max_pellets_fed_g / 1000                         # Convert to kg

# Step 3: Convert to dry matter basis
pellet_dm_kg <- max_pellets_fed_kg * (1 - pellet_moisture_pct / 100)

# Step 4: Calculate CP from pellets
pellet_cp_kg <- pellet_dm_kg * (pellet_cp_pct / 100)

# Step 5: Calculate basal diet intake and its CP
basal_dmi_kg <- bw_kg * (dmi_pct_bw / 100)
basal_cp_kg <- basal_dmi_kg * (basal_cp_pct / 100)

# Step 6: Determine pellet CP as % of basal diet CP
pellet_cp_pct_basal <- (pellet_cp_kg / basal_cp_kg) * 100

# Step 7: Print results
cat("Pellet CP Contribution Summary:\n")
```

```
## Pellet CP Contribution Summary:
```

```
cat("1. Max Pellets Fed (kg/day):", round(max_pellets_fed_kg, 3), "\n")
```

```
## 1. Max Pellets Fed (kg/day): 1.4
```

```
cat("2. Pellet DM (kg/day):", round(pellet_dm_kg, 3), "\n")
```

```
## 2. Pellet DM (kg/day): 1.232
```

```
cat("3. Pellet CP (kg/day):", round(pellet_cp_kg, 4), "\n")
```

```
## 3. Pellet CP (kg/day): 0.1848
```

```
cat("4. Basal DMI (kg/day):", round(basal_dmi_kg, 3), "\n")
```

```
## 4. Basal DMI (kg/day): 11.196
```

```
cat("5. Basal CP (kg/day):", round(basal_cp_kg, 3), "\n")
```

```
## 5. Basal CP (kg/day): 0.627
```

```
cat("6. Pellet CP % of Basal CP:", round(pellet_cp_pct_basal, 2), "%\n")
```

```
## 6. Pellet CP % of Basal CP: 29.47 %
```
